# Tubulin acetylation regulates nuclear shape fluctuations and DNA repair

**DOI:** 10.1038/s41556-026-02096-3

**Published:** 2026-10-28

**Authors:** Sam Wallis, Wai Yiu Tse, Lisa Donker, Anna Baxter, Nikita Dave, Andreas Weber, Eoin Gillespie, Baizhi Chen, Jonathan M. Burden, Anoek Friskes, René H. Medema, Guillaume Charras, Dinis P. Calado, Susana A. Godinho

**Affiliations:** 1 Centre for Cancer Cell and Molecular Biology, Barts Cancer Institute, Queen Mary University of London, London, UK; 2 London Centre for Nanotechnology, University College London, London, UK; 3 Immunity and Cancer Laboratory, Francis Crick Institute, London, UK; 4 Department of Cell Biology, Netherlands Cancer Institute, Amesterdam, the Netherlands; 5 Oncode Institute, Utrecht, the Netherlands; 6 Princess Máxima Center for Pediatric Oncology, Utrecht, the Netherlands; 7 Department of Cell and Developmental Biology, University College London, London, UK; 8 Immunity and Cancer Laboratory, Babraham Institute, Cambridge, UK; 9 Immunology Programme, Babraham Institute, Cambridge, UK

**Keywords:** Microtubules, Double-strand DNA breaks

## Abstract

Genome stability depends on the accurate repair of DNA double-strand breaks (DSBs). While chromatin-based mechanisms have been well studied, emerging evidence reveals that nuclear mechanics actively shape repair outcomes, although the mechanisms remain unclear. Here we identify microtubule acetylation as a regulator of nuclear mechanics that promotes DSB repair via homologous recombination (HR). DNA damage induces progressive tubulin acetylation, leading to displacement of the perinuclear vimentin cage. Loss of the vimentin cage softens the nucleus and renders the nuclear envelope responsive to microtubule forces, which drives transient nuclear shape fluctuations, localized DSB mobility and repair. Inhibiting tubulin acetylation abolishes these fluctuations, restricts DSB mobility and negatively impacts the homology search during HR, leaving non-homologous end joining intact. Consequently, persistent DSBs coalesce and cluster at the nuclear periphery, triggering cell-cycle arrest and senescence and increasing sensitivity to DNA-damaging agents. Removal of the vimentin cage is sufficient to rescue these effects, with restored nuclear fluctuations and repair efficiency. Thus, we propose that nuclear fluctuations act as a mechanical strategy to accelerate error-free repair. These findings reveal how cytoskeletal forces shape nuclear function and establish nuclear fluctuations as a fundamental principle for maintaining genome stability.

## Abstract (web summary)

Wallis, Tse et al. propose that DNA double-strand breaks (DSBs) induce microtubule acetylation, affecting nuclear shape and promoting DNA DSB mobility.

## Main

Genome integrity is constantly challenged by DNA double-strand breaks (DSBs), which are the most cytotoxic form of DNA damage that can lead to genomic instability, senescence or cell death^1,2^. In response to DSBs, the activation of a signalling cascade known as the DNA damage response (DDR) is crucial to pause cell-cycle progression and initiate repair. Cells have evolved sophisticated repair mechanisms to detect, process and resolve DSBs, including homologous recombination (HR) and non-homologous end joining (NHEJ), which are essential for genomic stability^3,4^. Accumulating evidence highlights that these mechanisms do not occur in isolation. Instead, they are coordinated within a dynamic nuclear landscape whose architecture and mechanics critically influence repair outcomes^5,6^.

Chromatin is highly dynamic, and its movement impacts processes such as DNA damage repair, with damaged sites exhibiting conserved increased mobility across species from yeast to humans^5,7,8^. Microtubules, which comprise αβ-tubulin heterodimers and are classically known for their roles in intracellular transport and mitosis, have emerged as key regulators of chromatin dynamics during the DDR^5,9^. In budding yeast, which contain intranuclear microtubules, microtubules play a key role in DNA damage repair (for example, by driving the kinesin-mediated transport of telomeric DSBs to the nuclear pore complex, where damage can be repaired)^10–12^. In mammals, where nuclear microtubules have not been described, cytoplasmic microtubules have been indirectly implicated in DDR. Seminal work^13^ from the de Lange laboratory demonstrated that the interaction between cytoplasmic microtubules and the nuclear envelope could impact DNA damage repair by promoting diffuse and localized DSB mobility that is independent of the actin cytoskeleton. Indeed, this type of mobility contrasts with nuclear actin-driven DSB mobility, which promotes long-range motility^14^ and is associated with the transport of DSBs to the nuclear periphery and often linked to persistent damage or challenging repair contexts such as heterochromatin^15–17^.

How microtubules respond to DNA damage remains poorly understood, but increased microtubule dynamics after IR has been shown to play an early role in DSB repair^18^. Additionally, changes in microtubule post-translational modifications downstream of DNA damage could also be involved in the DDR^9,19^. Indeed, the acetylation of α-tubulin on lysine 40 (K40) (hereafter referred to as tubulin acetylation), which is catalysed by the α-tubulin acetyltransferase αTAT1, is increased in response to various DNA-damaging agents and has been proposed to play a role in DNA damage repair^20–22^. Acetylated tubulin can facilitate kinesin-1 transport along microtubules (for example, through microtubule bundling^23^) and protects long-lived microtubules from breakage by increasing mechanical resilience; however, its role and regulation during the DDR is still elusive^24–27^.

The mechanism by which cytoplasmic microtubules regulate DSB mobility and repair has been unclear, but evidence suggests a role for the linker of nucleoskeleton and cytoskeleton (LINC) complex at the nuclear envelope, which transmits mechanical forces from the cytoplasm to chromatin^6,28^. Interaction between microtubules and the LINC complex occurs via kinesin motors, which promote the mobility of damaged DNA and the abnormal fusion of damaged telomeres or DSBs via NHEJ^13^. A more recent study showed that this interaction drives the formation of DSB-capturing nuclear envelope tubules that facilitate DSB repair^21^.

Changes in the nuclear mechanical properties also facilitate the transmission of forces to the chromatin^29^. The propensity for the nucleus to deform—and in extreme cases to rupture—depends on both the cytoskeleton forces applied to the nucleus and nuclear stiffness^6,30–33^. Interestingly, DNA damage leads to notable nuclear softening, suggesting that changes in nuclear mechanics could facilitate efficient force transmission from the LINC complex to the chromatin to potentially regulate its mobility^34^. However, the interplay between nuclear mechanics, microtubule-dependent forces and DSB mobility remains unclear.

In this Article, we demonstrate that tubulin acetylation regulates nuclear mechanics after DNA damage by promoting vimentin displacement from the nuclear periphery. This exposes the nucleus to microtubule-driven nuclear shape fluctuations and enhances localized DSB mobility, thereby promoting efficient HR. Blocking tubulin acetylation impairs DSB mobility, leading to the clustering of unrepairable DSBs, cell-cycle arrest and senescence, and renders cells more sensitive to DNA-damaging agents that target HR-mediated repair. These findings reveal a role for microtubules in coordinating nuclear mechanics and DNA repair and provide evidence that targeting tubulin acetylation could be used to enhance sensitivity to known cancer drugs that induce DNA damage.

## Results

### DNA damage-induced tubulin acetylation drives DSB mobility

**Fig. 1:**
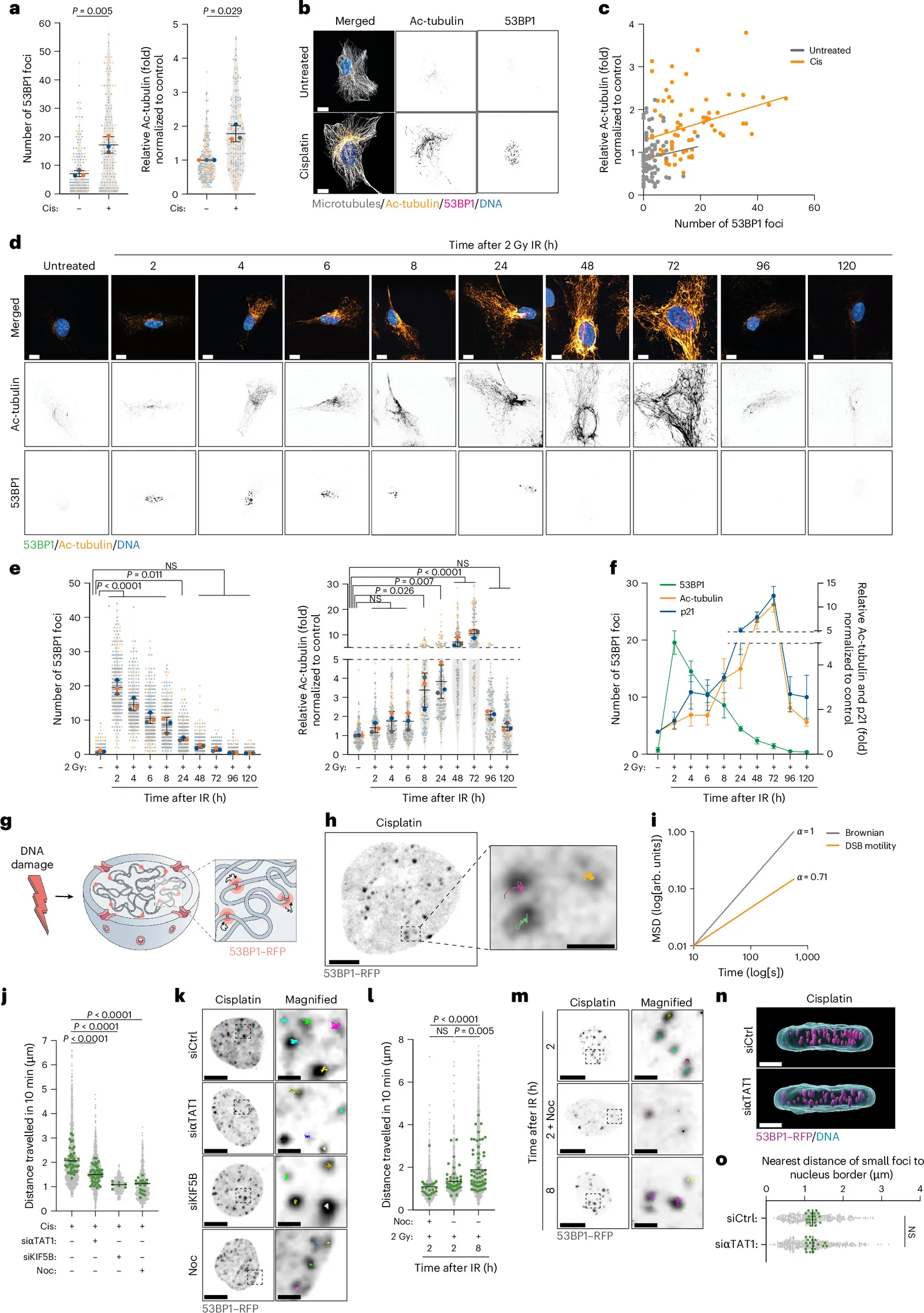
Tubulin acetylation drives DSB mobility downstream of DNA damage. **a**, Quantification of 53BP1 foci (left) and tubulin acetylation fluorescence intensity normalized to the control (right) before and after cisplatin treatment. Numbers of cells (left to right): *n* = 198 and 193. **b**, Representative images of cells with and without cisplatin treatment stained for acetylated tubulin (Ac-tubulin; orange), 53BP1 (magenta), microtubules (α-tubulin; grey) and DNA (Hoechst; cyan). Scale bars, 10 µm. **c**, Linear regression showing the correlation between acetylated tubulin levels and numbers of 53BP1 foci. Numbers of cells: *n* = 79 (untreated) and 78 (cisplatin). For untreated cells, *P* = 0.3887 and *R*^2^ = 0.009. For cisplatin-treated cells, *P* = 0.0043 and *R*^2^ = 0.102. **d**, Representative images of cells before and after IR stained for acetylated tubulin (Ac-tubulin; orange), 53BP1 (green) and DNA (Hoechst; cyan). Scale bars, 10 µm. **e**, Quantification of 53BP1 foci (left) and relative tubulin acetylation fluorescence intensity (right) 2–120 h after IR. Numbers of cells (left to right) for 53BP1: *n* = 325, 322, 290, 304, 315, 318, 277, 253, 316 and 310; and for Ac-tubulin: *n* = 172, 169, 174, 169, 181, 185, 184, 177, 155 and 155. **f**, Dynamics of 53BP1 foci, acetylated tubulin and nuclear p21 over time after IR. The data were derived from Fig. 1e and Extended Data Fig. 1f. **g**, Schematic representing the mobility of DSBs inside the nucleus. Credit: Reza Roozitalab. **h**, Still image from live-cell imaging of cells expressing 53BP1–RFP (grey) after cisplatin treatment. The lines show movement tracks of individual 53BP1 foci. Main scale bar, 5 µm. Inset scale bar, 1 µm. **i**, Anomalous diffusion coefficient of 53BP1 foci movement over time, showing sub-diffusive behaviour (*α* < 1). **j**, Total distance travelled in 10 min by 53BP1 foci in cisplatin-treated cells also treated with nocodazole (Noc) or depleted for αTAT1 or KIF5B. Numbers of cells (left to right): *n* = 43, 45, 7 and 20; and numbers of foci (left to right): *n* = 1,444, 1,265, 220 and 734. **k**, Representative still images from live-cell imaging of cells expressing 53BP1–RFP (grey) after receiving the same treatments described in **j**. The lines show the movement tracks of individual 53BP1 foci. Main scale bars, 10 µm. Inset scale bars, 2 µm. **l**, Total distance travelled by 53BP1 foci in 10 min at 2 and 8 h after IR with or without nocodazole treatment. Numbers of cells (left to right): *n* = 25, 50 and 59; and numbers of foci (left to right): *n* = 650, 1,158 and 1,134. **m**, Representative still images from live-cell imaging of cells expressing 53BP1–RFP (grey) after IR. The lines show the movement tracks of individual 53BP1 foci. Main scale bars, 10 µm. Inset scale bars, 2 µm. **n**, 3D surface rendering of nuclei (cyan) in live control and αTAT1-depleted cells expressing 53BP1–RFP (magenta) after cisplatin treatment. Scale bars, 5 µm. **o**, Quantification of the nearest distance of small foci to the nucleus border in cells treated with cisplatin and depleted of αTAT1. Numbers of cells (left to right): *n* = 20 and 20; and numbers of foci (left to right): *n* = 428 and 444. In **a** and **e**, the data represent means ± s.d. from three independent experiments, the small dots represent all cells analysed and the large dots represent the average of cells per experiment. In **f**, the dots represent means ± s.d. In **j**, **l** and **o**, the black bars represent the average movement of 53BP1 foci (grey dots), analysed per cell (green dots), from three independent experiments. *P* values are provided in the graphs. Statistical significance was determined by two-tailed unpaired *t*-test (**a**(left)), two-tailed one-sample *t*-test and Wilcoxon test (**a**(right)), simple linear regression (**c**), one-way analysis of variance (ANOVA) (**e**), one-way ANOVA with Kruskal–Wallis test (**j** and **i**) or two-tailed Mann–Whitney *U* test (**o**). NS, not statistically significant (*P* > 0.05). Source data

**Extended Data Fig. 1:**
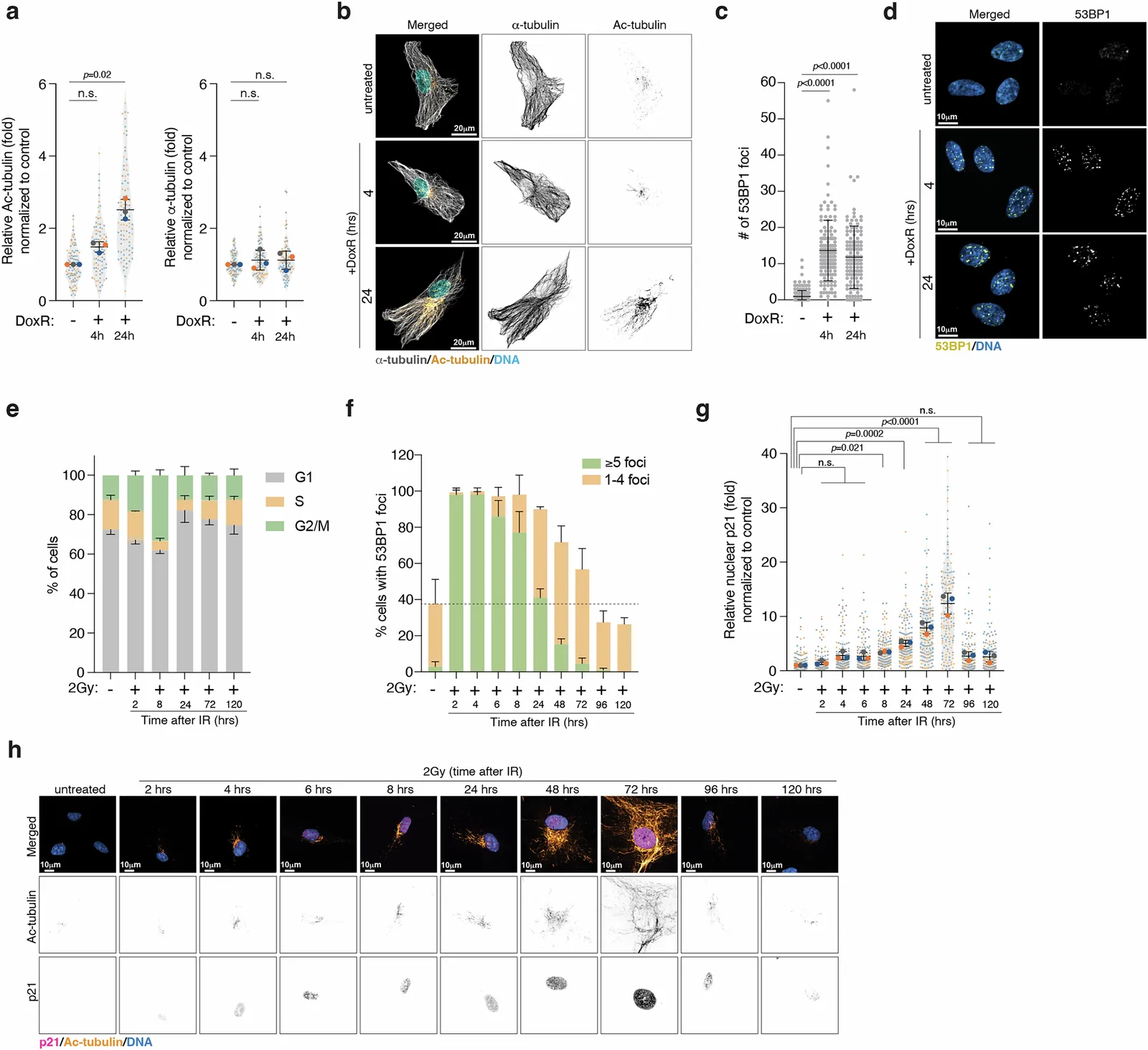
Tubulin acetylation is induced upon DNA damage. **a**, Quantification of normalized tubulin acetylation fluorescence intensity (left) and total α-tubulin fluorescence intensity (right) after doxorubicin treatment. Number of cells: n = 90/each condition. **b**, Representative images of cells with and without doxorubicin treatment stained for acetylated tubulin (Ac-tubulin, orange), microtubules (α-tubulin, grey), and DNA (Hoechst, cyan). Scale bar: 20 µm. **c**, Quantification of 53BP1 foci after doxorubicin. Number of cells: n = 171, 149, 124 (left to right). **d**, Representative images of cells after doxorubicin treatment stained for 53BP1 (yellow) and DNA (Hoechst, cyan). Scale bar: 10 µm. **e**, Percentage of cells at different phases of cell cycle after IR quantified by FACS. **f**, Percentage of cells with 53BP1 foci after IR. Number of cells: n = 325, 322, 290, 304, 315, 318, 277, 253, 316, 310. **g**, Quantification of nuclear p21 fluorescence intensity 2-120 h after IR. Number of cells: 230, 141, 176, 158, 178, 180, 184, 150, 178, 192. **h**, Representative images of cells before and after IR stained for acetylated tubulin (Ac-tubulin, orange), p21 (magenta), and DNA (Hoechst, cyan). Scale bar: 10 µm. **a,c,e,f,g** Error bars represent mean ± SD from three independent experiments. **a,g** Small dots: all cells analysed per experiment; Large dots: average of cells per experiment. *p* values are described in the graphs and calculated using one-way ANOVA with Kruskal-Wallis test for **a**,**c** and one-way ANOVA for **g**. All *P* < 0.05 and n.s. = not significant (*P* > 0.05). Source data

To study the dynamics and heterogeneity of microtubule acetylation in response to DNA damage, we employed single-cell image analyses of non-transformed RPE-1 cells treated with different genotoxic agents (details in Methods). Cisplatin treatment for 24 h led to an increase in tubulin acetylation of around twofold, which correlated with the level of DNA damage as assessed by p53-binding protein 1 (53BP1) foci, a marker of DSBs (Fig. 1a–c). Doxorubicin induced a similar number of DSBs at 4 and 24 h of treatment, but only the 24 h treatment caused a significant (~2.5-fold) increase in tubulin acetylation compared with the no-treatment control (at 4 h, the increase was only ~1.4-fold), highlighting a time-dependent response (Extended Data Fig. 1a–d). This phenotype is not a consequence of increased total microtubule mass, which remains unchanged in these conditions (Extended Data Fig. 1a). To better time the DDR response, we used ionizing radiation (IR), which leads to immediate DSBs, allowing repair dynamics to be monitored over time. Cells exposed to 2 Gy displayed a maximum number of DSBs at 2 h after IR; however, increased tubulin acetylation was not observed until 4–6 h, achieving a significant increase at 8 h (around threefold) and reaching a peak at 72 h (around tenfold) (Fig. 1d–f). Tubulin acetylation dramatically decreases after 96 h after IR, returning to control levels at 120 h, at a time when the number of DSB foci and cell-cycle profile of irradiated cells have returned to pre-irradiation levels (Fig. 1d–f and Extended Data Fig. 1e,f). Moreover, the dynamics of tubulin acetylation mirror the levels of nuclear p21, a late marker of DDR and cell-cycle arrest that can persist for several days after damage is induced^35,36^, suggesting that the decrease of tubulin acetylation at 96 h coincides with DDR termination (Fig. 1f and Extended Data Fig. 1g,h). These results demonstrate that tubulin acetylation is dynamically regulated and not an acute response to DNA damage, suggesting an association with later stages of DNA repair.

**Extended Data Fig. 2:**
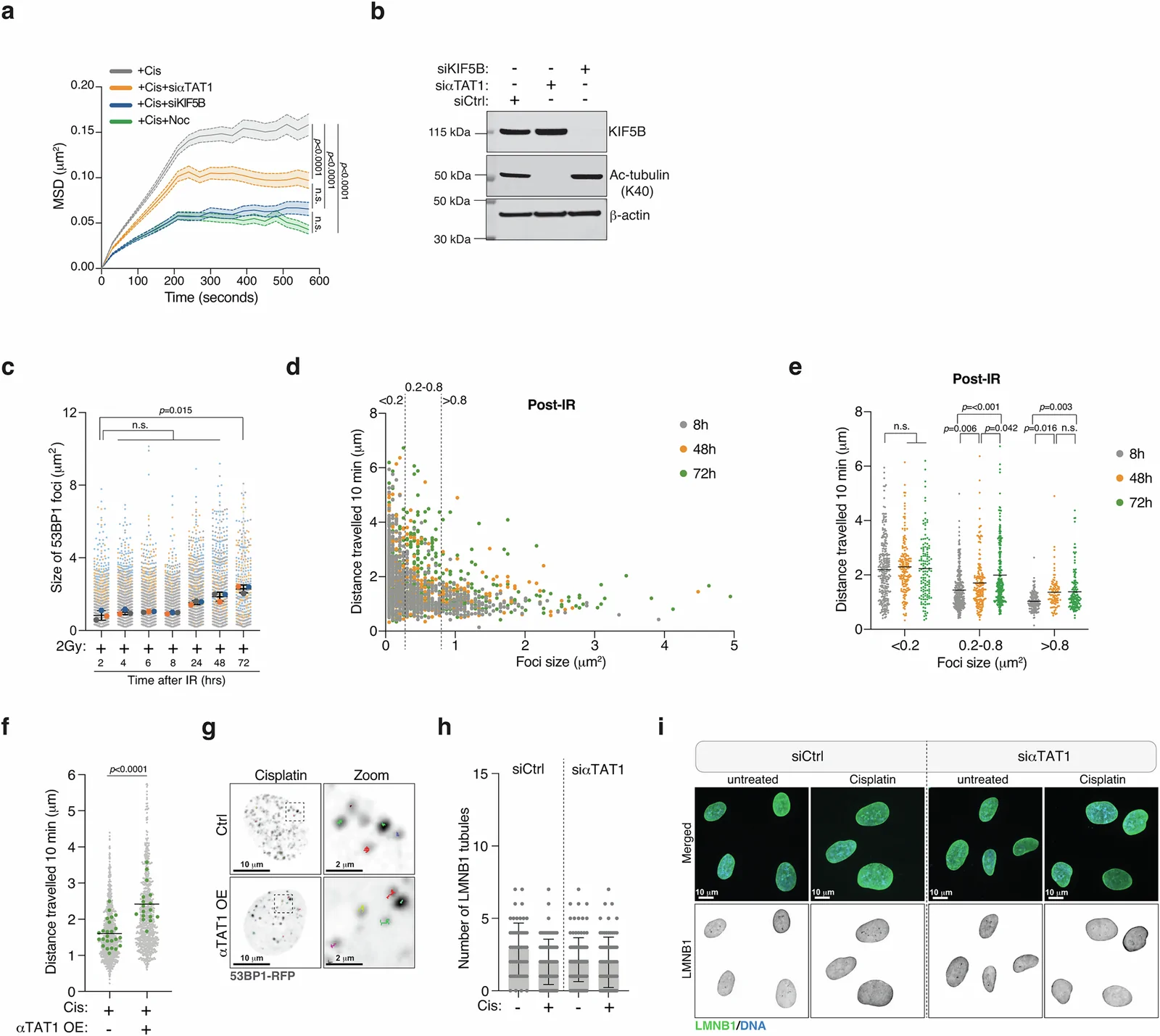
High levels of tubulin acetylation associated are associated with persistent DSBs and increased foci size. **a**, MSD analyses of 53BP1 foci mobility over time after cisplatin treatment and αTAT1, KIF5B depletion and nocodazole treatment. Number of cells/foci: n = 43/1444, 45/1265, 7/220, 20/734 (top to bottom). **b**, Western blot analyses with the indicated antibodies to confirm efficient depletion of KIF5B and αTAT1 (loss of tubulin acetylation). **c**, Size of 53BP1 foci after IR. Number of foci: n = 5130, 4365, 3229, 2703, 1415, 707, 334. **d**, Correlation between 53BP1 foci distance travelled during 10 min and foci size at 8, 48 and 72 hrs after IR. Number of foci: n = 743, 436, 519. **e**, 53BP1 foci distance travelled during 10 min separated by foci size after IR. **f**, 53BP1 foci distance travelled during 10 min in cells overexpressing αTAT1 and treated with cisplatin for 24 h. Number of cells/foci= 24/845, 17/808 (left to right). **g**, Representative still images from live-cell imaging of cells expressing 53BP1-RFP (grey) after cisplatin treatment; lines show movement tracks of individual 53BP1 foci. Scale bar: 10 µm; inset scale bar: 2 µm. **h**, Quantification of the number of lamin B1 (LMNB1) tubules as a surrogate for DSB-capturing nuclear envelope tubules after cisplatin treatment and αTAT1 depletion. Number of cells: n = 54, 81, 113, 80 (left to right). **i**, Representative images of cells with and without cisplatin treatment stained for lamin B1 (LMNB1, green) and DNA (Hoechst, cyan). Scale bar: 20 µm. **a** Data represents mean and SEM from three independent experiments. **c,h** Error bars represent mean ± SD. **c** Small dots: all cells analysed per experiment; Large dots: average of cells per experiment from three independent experiments. **h** Small dots: all nuclei analysed in one experiment. **e**, Horizontal bars represent average of 53BP1 foci movement for each foci from three independent experiments. **f**, Horizontal bars represent average of 53BP1 foci movement (grey dots) analysed per cell (small dots) from three independent experiments. **d** Line represents mean and dashed lines SEM. *p* values are described in the graphs and calculated using one-way ANOVA with Kruskal-Wallis test for **a,c**; two-way ANOVA for **e**; and two-tailed Mann-Whitney U test for **f**. All *P* < 0.05 and n.s. = not significant (*P* > 0.05). Source data

Because cytoplasmic microtubules can promote DSB mobility^13^, we asked whether tubulin acetylation could be the missing link between the DDR and DSB mobility. To test this, we performed live-cell imaging of RPE-1 cells expressing 53BP1 fused to red fluorescent protein (53BP1–RFP), to track DSBs^13^ (Fig. 1g,h and Supplementary Video 1). DSB mobility upon cisplatin treatment was very localized and exhibited random sub-diffusive motility, as indicated by mean squared displacement (MSD) analyses showing an anomalous diffusion coefficient (*α*) of <1 (where 1 represents Brownian diffusion) (Fig. 1i and Extended Data Fig. 2a). This type of random mobility, akin to a wiggly movement, was unlikely to have been driven by directed transport, consistent with other studies^8,10,13^.

αTAT1 depletion, to block tubulin acetylation^25^, significantly decreased DSB mobility, whereas loss of the kinesin-1 protein KIF5B (a microtubule motor protein previously implicated in this process^13^) or complete microtubule depolymerization by nocodazole treatment abrogated all movement (Fig. 1j,k, Extended Data Fig. 2b and Supplementary Video 2). These data were further validated by MSD analyses (Extended Data Fig. 2a). In irradiated cells, increased DSB mobility was observed at 8 h, but not at 2 h after IR exposure, consistent with the dynamic behaviour of tubulin acetylation (Fig. 1l,m and Supplementary Video 3). We further hypothesize that the increase in tubulin acetylation at later time points after IR could promote the DSB mobility of persistent breaks, which tend to be larger in size^37^ (Extended Data Fig. 2c). To do so, we assessed DSB mobility at 8, 48 and 72 h after IR (Extended Data Fig. 2d). We found that small foci (<0.2 μm^2^) moved faster and with similar mobility at all time points. However, for foci of increased size, DSB mobility was significantly higher at later time points, when high levels of tubulin acetylation were present (48 and 72 h), compared with that of foci of the same size at 8 h, suggesting that the further increase in tubulin acetylation could enable the mobility of larger DSB foci (Extended Data Fig. 2e). Overexpression of αTAT1 to further increase tubulin acetylation^25^ intensified DSB mobility in cisplatin-treated cells (Extended Data Fig. 2f,g and Supplementary Video 4). Acetylated microtubules have previously been implicated in the formation of DSB-capturing nuclear envelope tubules that facilitate repair by capturing DSBs at the nuclear envelope^21^. However, in cisplatin-treated cells, nuclear tubules (as quantified by lamin B1 staining) were not observed and remained unchanged upon the depletion of αTAT1 (Extended Data Fig. 2h,i). Consistently, three-dimensional (3D) reconstruction of nuclei expressing 53BP1–mCherry showed that αTAT1 depletion does not alter the spatial distribution of DSBs, most of which remain away from the nuclear periphery, pointing to an alternative role for acetylated microtubules in the DDR (Fig. 1n,o and Supplementary Videos 5 and 6). These results demonstrate that acetylated microtubules regulate localized DSB mobility and show that this mobility is temporally controlled downstream of DNA damage.

### Acetylated microtubules modulate nuclear mechanics during the DDR

**Fig. 2:**
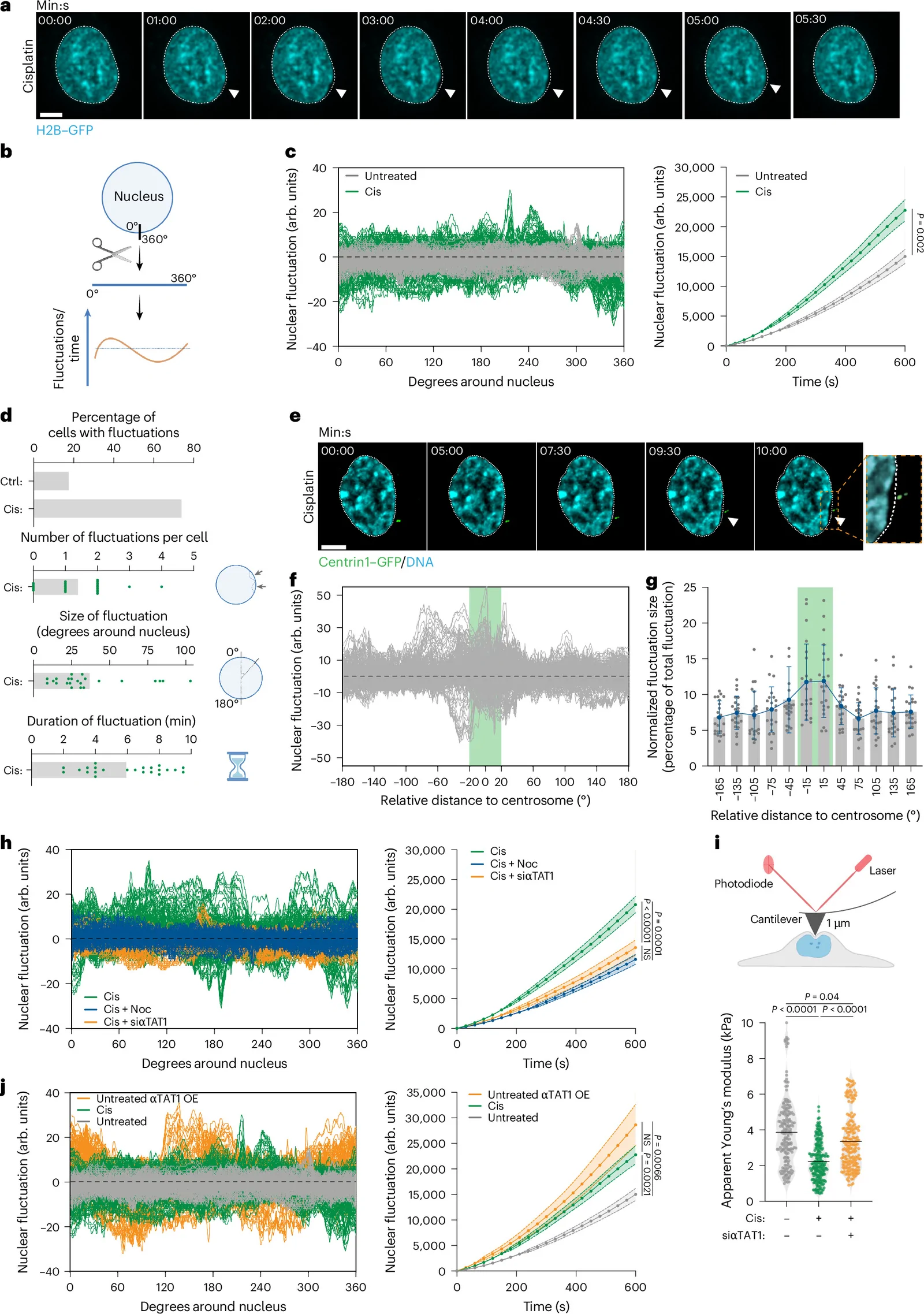
Tubulin acetylation regulates nuclear mechanics. **a**, Representative still images from live-cell imaging of cells expressing H2B–GFP (cyan) after cisplatin treatment. The dashed white line represents the initial nucleus outline and the white arrow marks a transient nuclear fluctuation. Scale bar, 5 µm. **b**, Schematic of polar transformation of the nucleus. **c**, Nuclear fluctuations across the nucleus (left) and cumulative nuclear fluctuations quantified over time (right) with and without cisplatin treatment. Numbers of cells: *n* = 29 (cisplatin) and 26 (untreated). **d**, Quantitative analyses of different nuclear fluctuation parameters with and without cisplatin treatment. Number of cells: *n* = 23. **e**, Representative still images from live-cell imaging of cells expressing centrin1–GFP (green) and DNA stained with Hoechst (cyan) after cisplatin treatment. The dashed white line represents the initial nucleus outline and the white arrow marks a transient nuclear fluctuation. Scale bar, 5 µm. **f**, Nuclear fluctuations across the nucleus, normalized to the initial centrosome position (0°), after cisplatin treatment. The −20° to 20° green area represents the average centrosome position over time. Number of cells: *n* = 24. **g**, Histogram of nuclear fluctuations around the nucleus relative to the centrosome position (0°). Each cell was normalized to their total cumulative fluctuations. Histogram bins were set to 30°. The green area represents the average centrosome position over time. Number of cells: *n* = 22. **h**, Nuclear fluctuations across the nucleus (left) and cumulative nuclear fluctuations quantified over time (right) after treatment with cisplatin only (*n* = 39), cisplatin + nocodazole (*n* = 21) or cisplatin plus αTAT1 depletion (*n* = 35). **i**, Schematic of AFM (top) and quantification of the apparent Young’s modulus in cells treated (or not) with cisplatin and depleted (or not) of αTAT1 (bottom). Number of measurements (left to right): *n* = 194, 227 and 194. **j**, Nuclear fluctuations across the nucleus (left) and cumulative nuclear fluctuations quantified over time (right) in cells overexpressing αTAT1 (untreated αTAT1 OE; *n* = 22), cells with regular expression of αTAT1 treated with cisplatin (*n* = 26) and cells with regular expression of αTAT1 left untreated (*n* = 29). The values for untreated and cisplatin are shared with **c**. In the left panels of **c**, **f**, **h** and **j**, the lines represent individual nuclei at all time points. In the right panels of **c**, **f**, **h** and **j**, the solid lines represent means and the dashed lines and shading represent s.e.m. from three independent experiments. In **g**, the data represent means ± s.d. and the small dots represent individual values per cell. In **i**, the horizontal lines represent the average per nucleus. *P* values are provided in the graphs. Statistical significance in **c** and **h**–**j** was determined by one-way ANOVA with Kruskal–Wallis test. Schematics in **b** and **d** created in BioRender; Godinho, S. https://biorender.com/x1kc4pa (2026). Source data

The interaction between cytoplasmic microtubules and the LINC complex at the nuclear envelope has been shown to be important for DSB mobility^13^. This is consistent with the idea that forces applied to the nucleus could drive localized chromatin mobility. To determine whether microtubules could generate sufficient force at the nuclear envelope, we performed live-cell imaging of nuclei of untreated and cisplatin-treated cells to monitor nuclear deformations. Strikingly, cisplatin-treated cells undergo transient and localized nuclear fluctuations, consistent with nuclear deformations (Fig. 2a and Supplementary Video 7). We developed a computational method to quantify nuclear fluctuations by transforming the circular nuclear outline from Cartesian coordinates (*x*, *y*) into polar coordinates (*r*, *θ*) and normalizing to the initial frame. This approach effectively unwraps the circular nucleus into a linear representation of nuclear shape change over time for easier visualization and analysis (Fig. 2b; details in Methods). Using this approach, we uncovered that DNA damage leads to changes in nuclear shape represented by the linear representation of nuclear outlines, as well as a cumulative total change in nuclear shape (hereby referred to as cumulative nuclear fluctuations) over time (Fig. 2c). Approximately 70% of cisplatin-treated cells display nuclear fluctuations, which are consistent in size and number (one or two) but vary in duration (2–10 min) (Fig. 2d). The localized nature of these nuclear oscillations is consistent with force generation driven by microtubule nucleation near the nuclear envelope. We postulated that microtubules nucleated by the centrosome (the main microtubule organizing centre, located adjacent to the nucleus) would be ideally positioned to generate localized force that deforms the nuclear envelop. Live-cell imaging of cells expressing the centriole marker centrin1 fused to green fluorescent protein (centrin1–GFP) allowed us to position nuclear fluctuations relative to the centrosome by normalizing the average centrosome position across the duration of the video to 0°. We found that the average centrosome position (in the −20 to 20° range; details in Methods) was strongly associated with areas of larger nuclear oscillations. By grouping nuclear areas according to the distance to the centrosome, we were able to map the larger fluctuations to areas closer to the centrosome (Fig. 2e–g and Supplementary Video 8).

**Extended Data Fig. 3:**
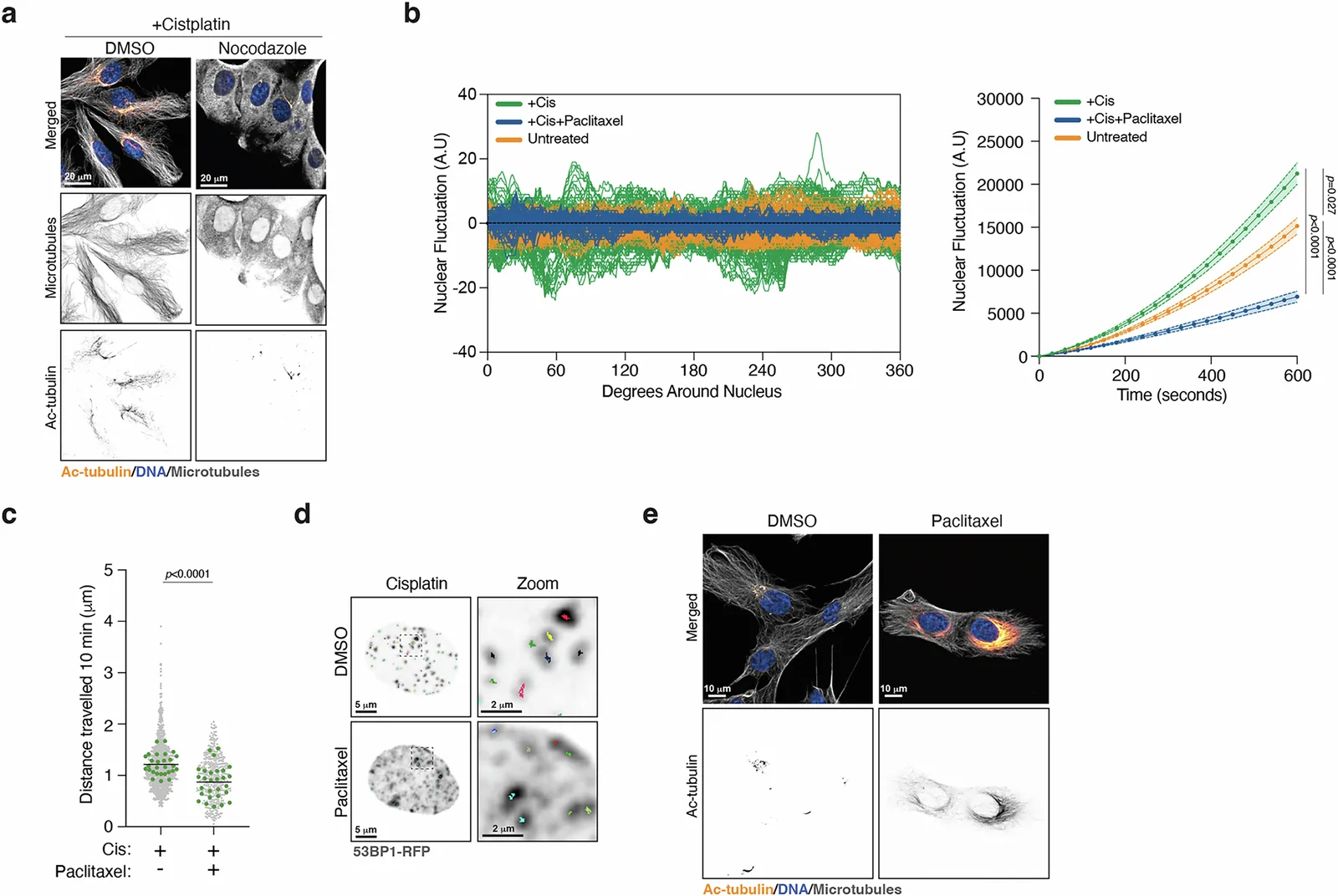
Inhibition of microtubule dynamics with paclitaxel prevents DSB mobility and nuclear fluctuations. **a**, Representative images of cisplatin treated cells after nocodazole treatment to depolymerize microtubules stained for acetylated tubulin (Ac-tubulin, orange), microtubules (α-tubulin, grey) and DNA (Hoechst, cyan). Scale bar: 20 µm. **b**, Nuclear fluctuations across the nucleus (left) and cumulative nuclear fluctuations quantified over time (right) after cisplatin treatment. Number of cells: n = 28, 28 and 27 (top to bottom). Left graphs: lines represent individual nuclei at all time points; Right graphs: line represents mean and dashed lines SEM. **c**, 53BP1 foci distance travelled during 10 min in cells treated with cisplatin and paclitaxel (number of cells/foci=28/981, 28/404) (left to right). **d**, Representative still images from live-cell imaging of cells expressing 53BP1-RFP (grey) after cisplatin treatment; lines show movement tracks of individual 53BP1 foci. Scale bar: 5 µm; inset scale bar: 2 µm. **e**, Representative images of cells treated with paclitaxel to stabilize microtubules stained for acetylated tubulin (Ac-tubulin, orange), microtubules (α-tubulin, grey) and DNA (Hoechst, cyan). Scale bar: 10 µm. **b** Line represents mean and dashed lines SEM from three independent experiments. **c** Horizontal bars represent average of 53BP1 foci movement (grey dots) analysed per cell (green dots) from three independent experiments. *p* values are described in the graphs and calculated using one-way ANOVA for **b** and two-tailed Welch’s t-test for **c**. All *P* < 0.05. Source data

Microtubule depolymerization by nocodazole abrogated nuclear fluctuations, demonstrating that centrosomal microtubules can indeed apply sufficient force to deform the nucleus. Moreover, specific loss of acetylated microtubules was sufficient to prevent nuclear fluctuations upon DNA damage, mirroring complete microtubule depolymerization (Fig. 2h and Extended Data Fig. 3a). Paclitaxel treatment to stabilize microtubules also abrogates nuclear fluctuations (Extended Data Fig. 3b) and, as was previously reported^13^, DSB mobility (Extended Data Fig. 3c,d and Supplementary Video 9). Thus, even in the presence of increased levels of tubulin acetylation promoted by paclitaxel^38^ (Extended Data Fig. 3e), dynamic microtubules are required to promote nuclear fluctuations.

We next tested whether nuclear fluctuations were associated with changes in nuclear mechanics. Using atomic force microscopy (AFM) to measure the viscoelastic properties of the nucleus^39^, we observed that nuclear stiffness decreased in response to DNA damage, as was previously shown^34^, but interestingly, depletion of αTAT1 was sufficient to significantly prevent nuclear softening in cells with DNA damage (Fig. 2i). Thus, nuclear fluctuations are associated with decreased nuclear stiffness downstream of DNA damage, a process regulated by tubulin acetylation.

**Extended Data Fig. 4:**
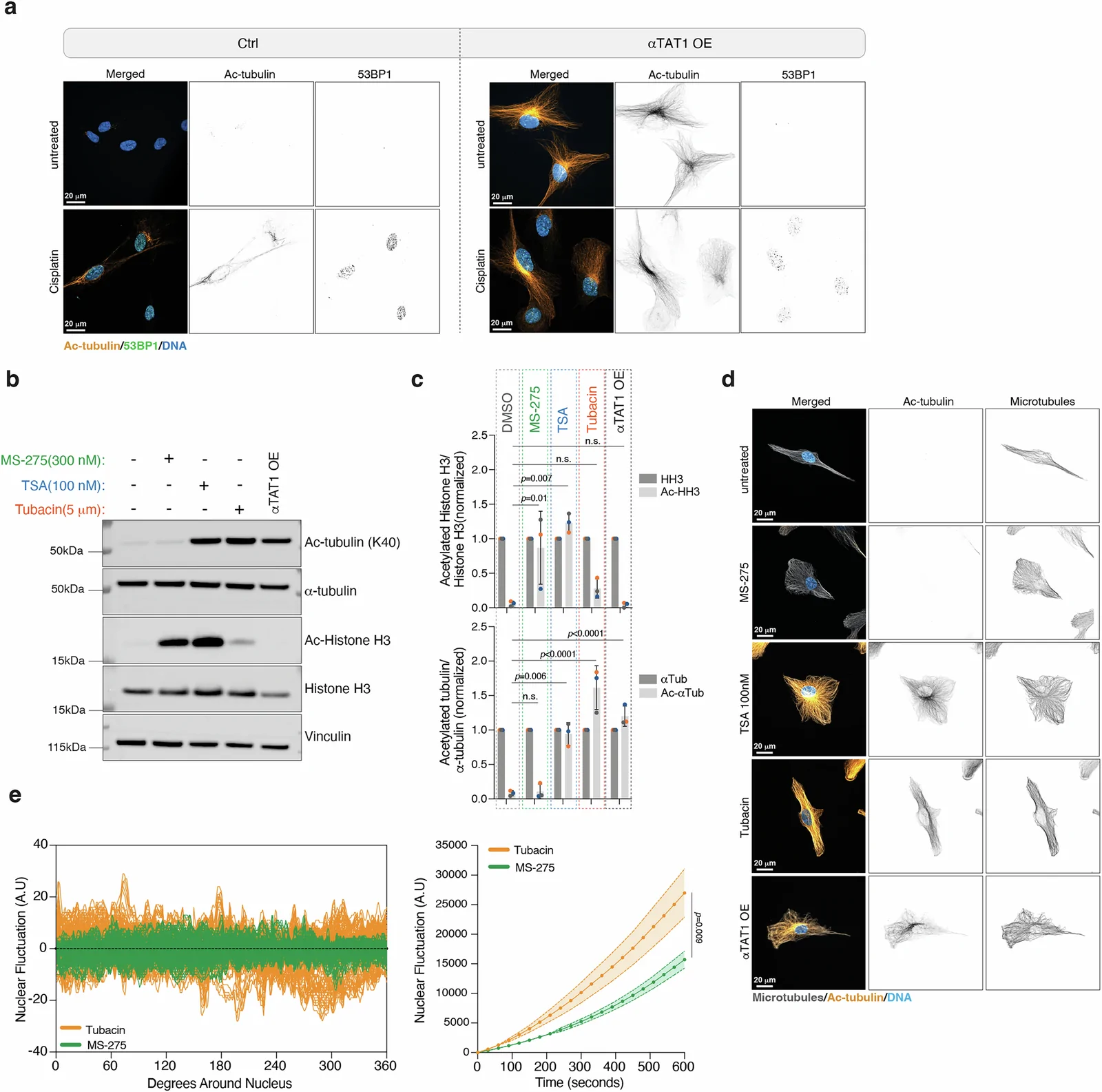
Tubulin acetylation, but not histone acetylation, promotes nuclear fluctuations. **a**, Representative images of control and αTAT1 overexpressing cells with and without cisplatin treatment stained for acetylated tubulin (Ac-tubulin, orange), 53BP1 (green), and DNA (Hoechst, cyan). Scale bar: 20 µm. **b**, Western blot analyses with the indicated antibodies after treatment with HDAC inhibitors (MS-275, trichostatin A, tubacin) and αTAT1 overexpression. **c**, Quantification of the Western blot data showing the levels of histone H3 and α-tubulin acetylation, normalized to total histone H3 and total tubulin, respectively, upon the different conditions/treatments. Error bars represent mean ± SD from three independent experiments and large dots average values for each experiment. **d**, Representative images of cells, matching the Western blot in **b**, stained for acetylated tubulin (Ac-tubulin, orange), microtubules (α-tubulin, grey), and DNA (Hoechst, cyan). Scale bar: 20 µm. **e**, Nuclear fluctuations across the nucleus (left) and cumulative nuclear fluctuations quantified over time (right) after cisplatin treatment. Number of cells: n = 11 and 14 (top to bottom). Left graphs: lines represent individual nuclei at all time points; Right graphs: line represents mean and dashed lines SEM from three independent experiments. *p* values are described in the graphs and calculated using one-way ANOVA for **c** and two-tailed unpaired t-test for **e**. All *P* < 0.05 and n.s. = not significant (*P* > 0.05). Source data

**Extended Data Fig. 5:**
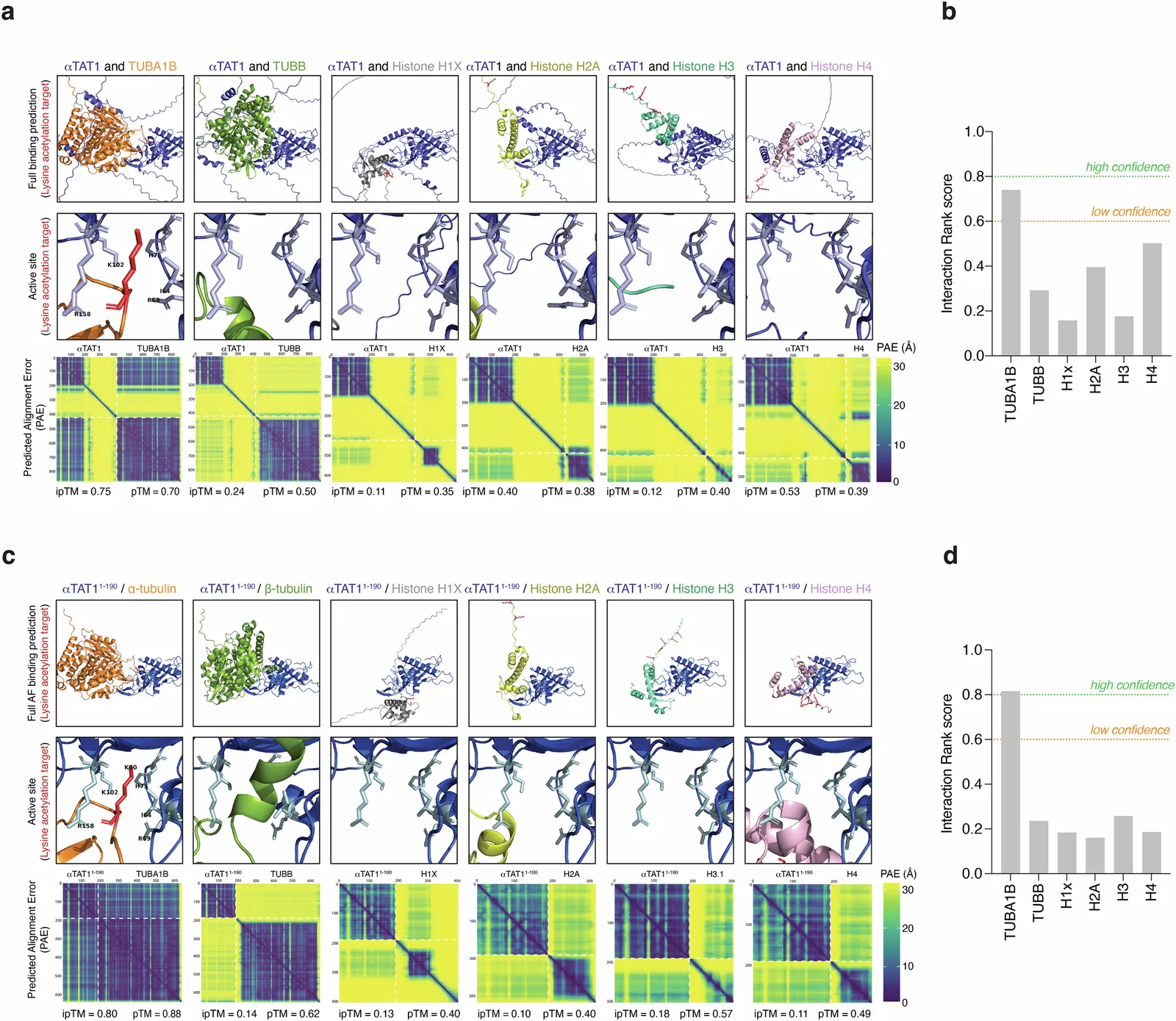
Alphafold3 structural prediction supports αTAT1 specificity for α-tubulin. **a**, PyMol cartoon diagrams depicting the structure of Alphafold3 (AF3) predicted interactions between αTAT1 and α-tubulin, β-tubulin, and Histones H1x, H2A, H3, and H4 (top). Known lysine acetylation sites are shown as red sticks. The active site of αTAT1 aligns with known acetylation site K40 of α-tubulin (middle). Predicted alignment error (PAE) matrices show the confidence in relative positions of residue pairs (bottom). **b**, Interaction Rank score. Horizontal lines represent confidence for predicted interactions. **c**, PyMol cartoon diagrams depicting the structure of Alphafold3 (AF3) predicted interactions between truncated acetyltransferase domain-only αTAT1^1–190^ and α-tubulin, β-tubulin, and Histones H1x, H2A, H3, and H4. **d**, Interaction Rank score. Horizontal lines represent confidence for predicted interactions. Source data

Nuclear mechanics during DDR can be impacted by changes in chromatin organization or activation of the kinases ATR and ATM^34,40–43^. To assess the specificity of tubulin acetylation in the regulation of nuclear mechanics, we overexpressed αTAT1 to induce high levels of tubulin acetylation independent of DNA damage. Indeed, this was sufficient to drive nuclear fluctuations in cells without DNA damage (~50% of cells; Fig. 2j and Extended Data Fig. 4a). In addition, cells were treated with different histone deacetylase (HDAC) inhibitors to examine the impact of chromatin versus tubulin acetylation (Extended Data Fig. 4b–d). Among the different inhibitors tested, we selected tubacin, a HDAC6 inhibitor that promotes efficient tubulin acetylation but not histone H3 acetylation, and MS-275, a class I HDAC inhibitor, which promotes histone H3 acetylation but has no impact on tubulin acetylation (Extended Data Fig. 4b–d). Similarly to αTAT1 overexpression, tubacin treatment was sufficient to promote nuclear fluctuations in the absence of DNA damage, but MS-275 was not (Extended Data Fig. 4e). Moreover, and supporting previous works suggesting that αTAT1 does not acetylate histones^44,45^, AlphaFold3 modelling^46^ predicted a strong interaction between αTAT1 and α-tubulin, as expected, but not between αTAT1 and β-tubulin or histones (H1X, H2A, H3 and H4) (Extended Data Fig. 5a,b). The interface predicted template modelling (ipTM) score between αTAT1 and α-tubulin further increases by removing the intrinsically disordered carboxy-terminal region and using only the *N*-acetyltransferase domain of αTAT1 (amino acids 1–190) (Extended Data Fig. 5c,d)^47^. Mapping of this interaction was also consistent with the high specificity of αTAT1 targeting K40 on α-tubulin (Extended Data Fig. 5a,c). Hence, it is unlikely that αTAT1 interacts with and acetylates histones. Altogether, these data demonstrate that acetylated microtubules are the main regulators of the mechanical properties of the nucleus during the DDR.

### Vimentin displacement from the nucleus promotes nuclear fluctuations and DSB mobility

**Fig. 3:**
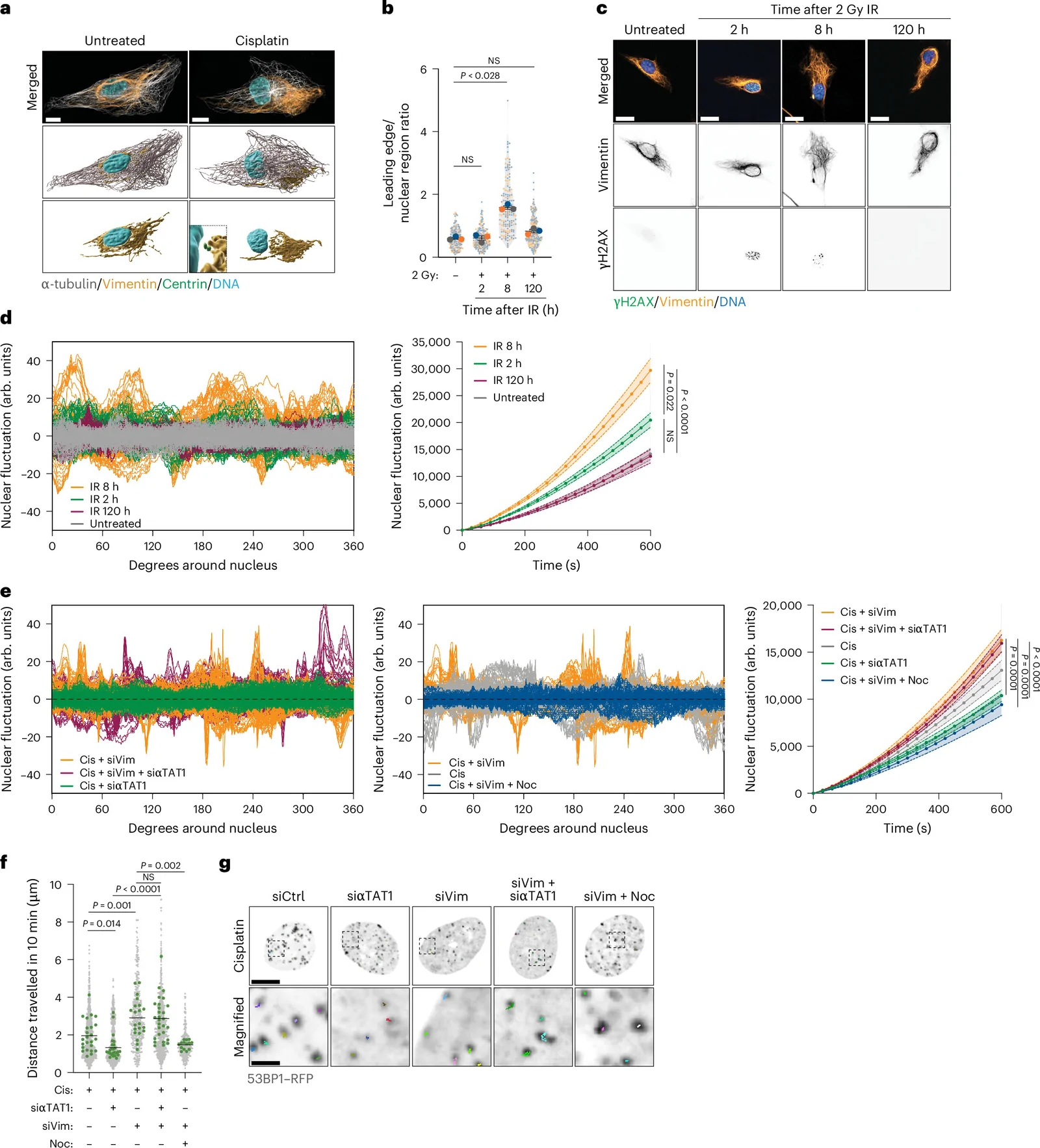
Vimentin displacement promotes nuclear fluctuations. **a**, Representative images of cells with and without cisplatin treatment stained for vimentin (orange), microtubules (α-tubulin; grey) and DNA (Hoechst; cyan), and 3D surface rendering. Scale bars, 10 µm. **b**, Quantification of the vimentin leading edge/nuclear ratio after irradiation. Numbers of cells (left to right): *n* = 148, 154, 154 and 162. **c**, Representative images of cells after IR stained for vimentin (orange), γH2AX (green) and DNA (Hoechst; cyan). Scale bars, 20 µm. **d**, Nuclear fluctuations across the nucleus (left) and cumulative nuclear fluctuations quantified over time (right) at 2 h (*n* = 38), 8 h (*n* = 47) and 120 h after IR (*n* = 27) and in untreated cells (*n* = 27). **e**, Nuclear fluctuations across the nucleus (left and middle) and cumulative nuclear fluctuations quantified over time (right) in cisplatin-treated cells with or without vimentin depletion, αTAT1 depletion or nocodazole treatment. Numbers of cells (top to bottom in rightmost panel): *n* = 26, 38, 25, 28 and 15. **f**, Total distance travelled by 53BP1 foci in 10 min after cisplatin treatment with or without αTAT1 depletion, vimentin depletion or nocodazole treatment. Numbers of cells (left to right): *n* = 24, 25, 21, 28 and 14; and numbers of foci (left to right): *n* = 905, 844, 447, 705 and 312. **g**, Representative still images from live-cell imaging of cells expressing 53BP1–RFP (grey) after cisplatin treatment. The lines show the movement tracks of individual 53BP1 foci. Main scale bar, 10 µm. Inset scale bar, 2 µm. In **b**, the data represent means ± s.d. from three independent experiments, the small dots represent all cells analysed and the large dots represent the average of cells per experiment. In **d**(left) and **e**(middle and left), the lines represent individual nuclei at all time points. In **d**(right) and **e**(right), the solid lines represent means and the dashed lines and shaded areas represent s.e.m. from three independent experiments. In **f**, the horizontal bars represent the average movement of 53BP1 foci (grey dots) analysed per cell (green dots) from three independent experiments. *P* values are provided in the graphs. Statistical significance in **b** and **d**–**f** was determined by one-way ANOVA with Kruskal–Wallis test. Source data

**Extended Data Fig. 6:**
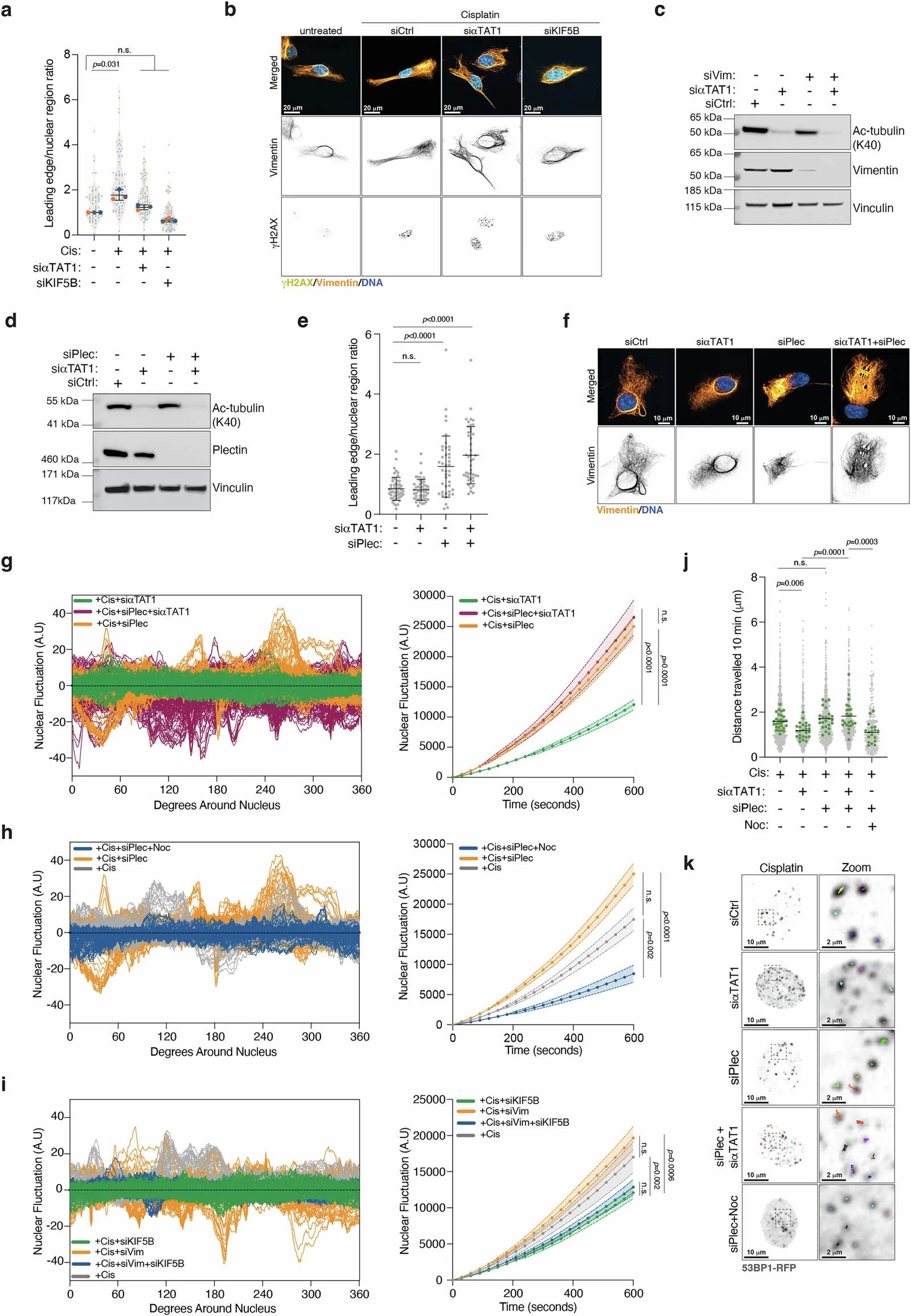
Vimentin displacement away from the nucleus promotes nuclear fluctuations and DSB mobility. **a**, Quantification of vimentin leading edge/nuclear ratio after cisplatin and depletion of αTAT1 or KIF5B. Number of cells: n = 67, 81, 80, 76 (left to right). **b**, Representative images of cells stained for vimentin (orange), γH2AX (yellow) and DNA (Hoechst, cyan). Scale bar: 20 µm. **c**, Western blot analyses with the indicated antibodies to confirm efficient depletion of vimentin and αTAT1. **d**, Western blot analyses with the indicated antibodies to confirm efficient depletion of plectin and αTAT1. **e**, Quantification of vimentin leading edge/nuclear ratio after cisplatin and depleted of αTAT1 and plectin. Number of cells: n = 51, 51, 42, 44 (left to right). **f**, Representative images of cells stained for vimentin (orange) and DNA (Hoechst, cyan) after depletion of αTAT1 or plectin. Scale bar: 10 µm. **g**, Nuclear fluctuations across the nucleus (left) and cumulative nuclear fluctuations quantified over time (right) after cisplatin treatment and depletion of αTAT1 and plectin. Number of cells: n = 22, 18, 23 (top to bottom). **h**, Nuclear fluctuations across the nucleus (left) and cumulative nuclear fluctuations quantified over time (right) after cisplatin treatment and plectin depletion and nocodazole treatment. Number of cells: n = 23, 23, 24 (top to bottom). **i**, Nuclear fluctuations across the nucleus (left) and cumulative nuclear fluctuations quantified over time (right) after cisplatin treatment and KIF5B depletion. Number of cells: n = 22, 33, 27, 22 (top to bottom). **j**, 53BP1 foci distance travelled during 10 min in cells treated with cisplatin and depleted of αTAT1 and plectin or treated with nocodazole (number of cells/foci=28/1180, 25/741, 24/943, 24/645, 18/352) (left to right). **k**, Representative still images from live-cell imaging of cells expressing 53BP1-RFP (grey) after cisplatin treatment; lines show movement tracks of individual 53BP1 foci. Scale bar: 10 µm; inset scale bar: 2 µm. **a,e** Error bars represent mean ± SD from three independent experiments. **a**, Small dots: all cells analysed per experiment; Large dots: average of cells per experiment. **g,h,i** Left graphs: lines represent individual nuclei at all time points; Right graphs: line represents mean and dashed lines SEM from three independent experiments. **j** Horizontal bars represent average of 53BP1 foci movement (grey dots) analysed per cell (green dots). Data from three independent experiments. *p* values are described in the graphs and calculated using one-way ANOVA for **a**, and one-way ANOVA with Kruskal-Wallis test for **e,g,h,i,j**. All *P* < 0.05 and n.s. = not significant (*P* > 0.05). Source data

Vimentin, a type III intermediate filament, forms a cage around the nucleus to enhance stiffness and protect against mechanical stress^48,49^. However, this vimentin cage can be displaced through directed transport along acetylated microtubules by kinesin-1 motors^25,50–52^. With this in mind, we asked whether vimentin displacement away from the nucleus could explain the decrease in nuclear stiffness in cells with DNA damage, thereby promoting nuclear fluctuations. We first confirmed that DNA damage led to vimentin displacement away from the nucleus, exposing the nucleus to centrosomal microtubules (Fig. 3a), a process that requires tubulin acetylation and KIF5B (Extended Data Fig. 6a,b). In irradiated cells, vimentin displacement and nuclear fluctuations followed the dynamic pattern of tubulin acetylation occurring at 8 h, but not at 2 h. At 120 h, when the levels of tubulin acetylation were restored to pre-DNA-damage levels, the nuclear vimentin cage was re-established and fluctuations were no longer observed (Fig. 3b–d). These data support an association between loss of the vimentin cage and nuclear fluctuations.

We hypothesize that if displacement of the vimentin cage were essential for nuclear fluctuations, depletion of vimentin in cells that lack tubulin acetylation should restore nuclear fluctuations in cisplatin-treated cells. Indeed, co-depletion of vimentin and αTAT1 rescued nuclear fluctuations, indicating that acetylated microtubules promote nuclear dynamics by displacing vimentin (Fig. 3e and Extended Data Fig. 6c). To further confirm these results independent of vimentin loss, vimentin displacement was induced through depletion of the cytoskeletal cross-linking protein plectin (Extended Data Fig. 6d–f), as was previously shown^53^. Co-depletion of plectin and αTAT1 was also sufficient to restore nuclear fluctuations in cisplatin-treated cells (Extended Data Fig. 6g). Neither vimentin nor plectin depletion restored nuclear fluctuations in the absence of microtubules (nocodazole treatment), demonstrating that microtubules are essential to apply forces that deform the nuclear envelope, even in the absence of the nuclear vimentin cage (Fig. 3e and Extended Data Fig. 6h). Similarly, and in contrast with αTAT1, depletion of KIF5B decreased nuclear fluctuations even in the absence of vimentin, supporting an additional role for kinesin-1 in force generation at the nuclear envelope to drive nuclear fluctuations (Extended Data Fig. 6i). Vimentin or plectin depletion was also sufficient to rescue DSB mobility in αTAT1-depleted cells, but not in cells treated with nocodazole (Fig. 3f,g, Extended Data Fig. 6j,k and Supplementary Videos 10 and 11). Taken together, these data establish that microtubule acetylation in response to DNA damage regulates vimentin displacement, allowing microtubule-driven nuclear fluctuations and promoting DSB mobility.

### DSB mobility promotes efficient HR-mediated DNA repair

**Fig. 4:**
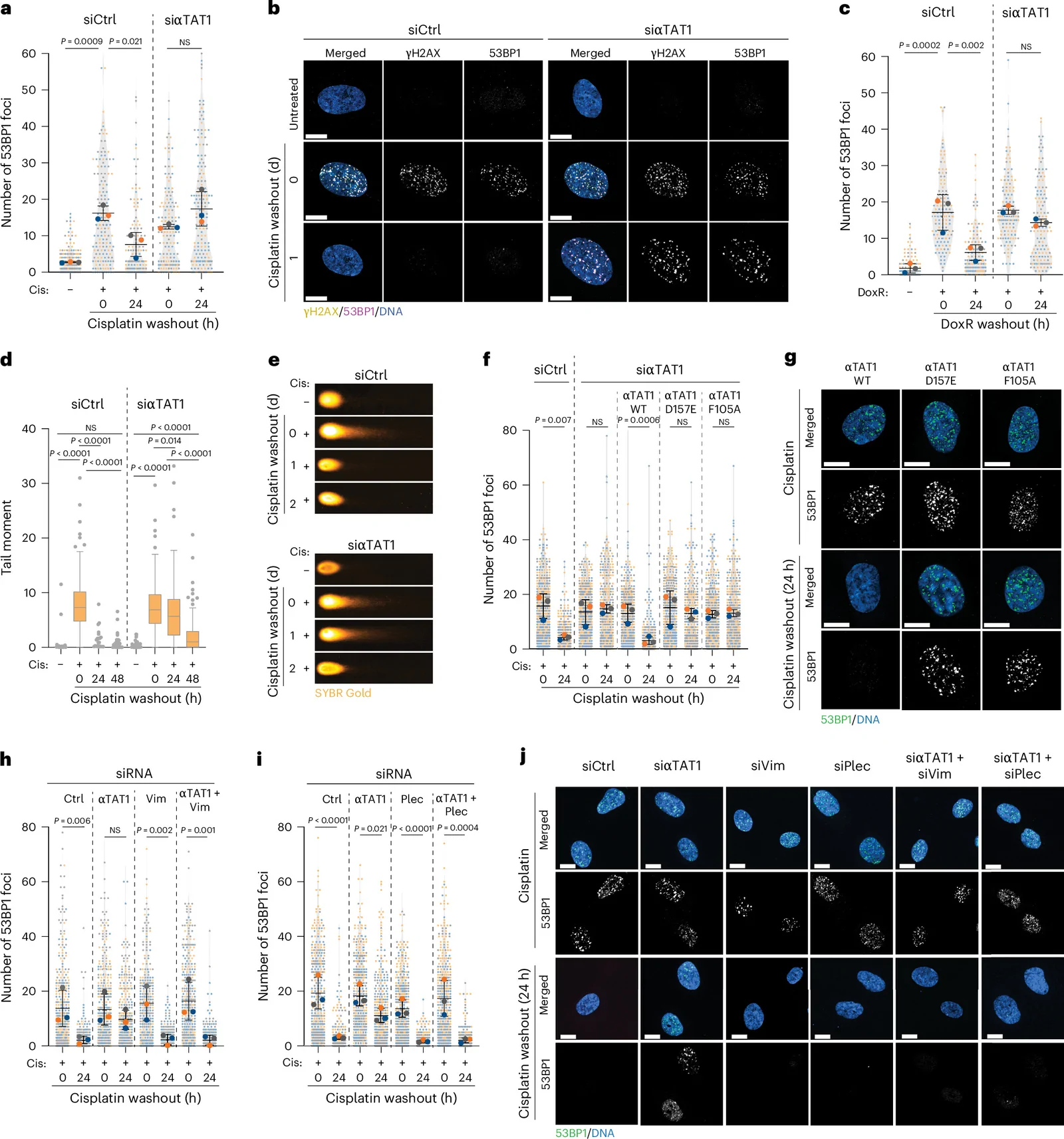
Restoring DSB mobility rescues DNA repair in αTAT1-depleted cells. **a**, Quantification of 53BP1 foci after cisplatin washout and αTAT1 depletion. Numbers of cells (left to right): *n* = 195, 156, 129, 137 and 163. **b**, Representative images of cells after cisplatin washout stained for γH2AX (yellow), 53BP1 (magenta) and DNA (Hoechst; cyan). Scale bars, 10 µm. **c**, Quantification of 53BP1 foci after doxorubicin (DoxR) washout and αTAT1 depletion. Numbers of cells (left to right): *n* = 136, 120, 120, 126 and 128. **d**, Tail moment quantification after cisplatin washout and αTAT1 depletion. Numbers of nuclei (left to right): *n* = 185, 162, 192, 145, 133, 187, 220 and 145. **e**, Representative images of comet assay nuclei stained for SYBR Gold after cisplatin washout. **f**, Quantification of 53BP1 foci after cisplatin washout in wild-type αTAT1, αTAT1 D157E and αTAT1 F105A cells upon αTAT1 depletion. Numbers of cells (left to right): *n* = 338, 365, 343, 294, 378, 413, 359, 293, 363 and 326. **g**, Representative images of cells after cisplatin washout stained for 53BP1 (green) and DNA (Hoechst; cyan). Scale bars, 10 µm. **h**, Quantification of 53BP1 foci after cisplatin washout and vimentin and αTAT1 depletion. Numbers of cells (left to right): *n* = 398, 310, 379, 346, 268, 292, 321 and 349. **i**, Quantification of 53BP1 foci after cisplatin washout and plectin and αTAT1 depletion. Numbers of cells (left to right): *n* = 305, 307, 308, 321, 306, 331, 342 and 304. **j**, Representative images of cells after cisplatin washout stained for 53BP1 (green) and DNA (Hoechst; cyan). Scale bars, 10 µm. In **a**, **c**, **f**, **h** and **i**, the data represent means ± s.d. from three independent experiments, the small dots represent all cells analysed and the large dots represent the average of cells per experiment. In **d**, for the box plot with Tukey whiskers, the centre line depicts the median, the box edges represent the 25th and 75th percentiles and whiskers depict the most extreme values within 1.5 × interquartile (Tukey). Grey dots are cells beyond the whiskers. Data were obtained from three independent experiments. *P* values are provided in the graphs. Statistical significance was determined by one-way ANOVA (**a** and **c**), one-way ANOVA with Kruskal–Wallis test (**d**) or two-way ANOVA (**f**, **h** and **i**). Source data

**Extended Data Fig. 7:**
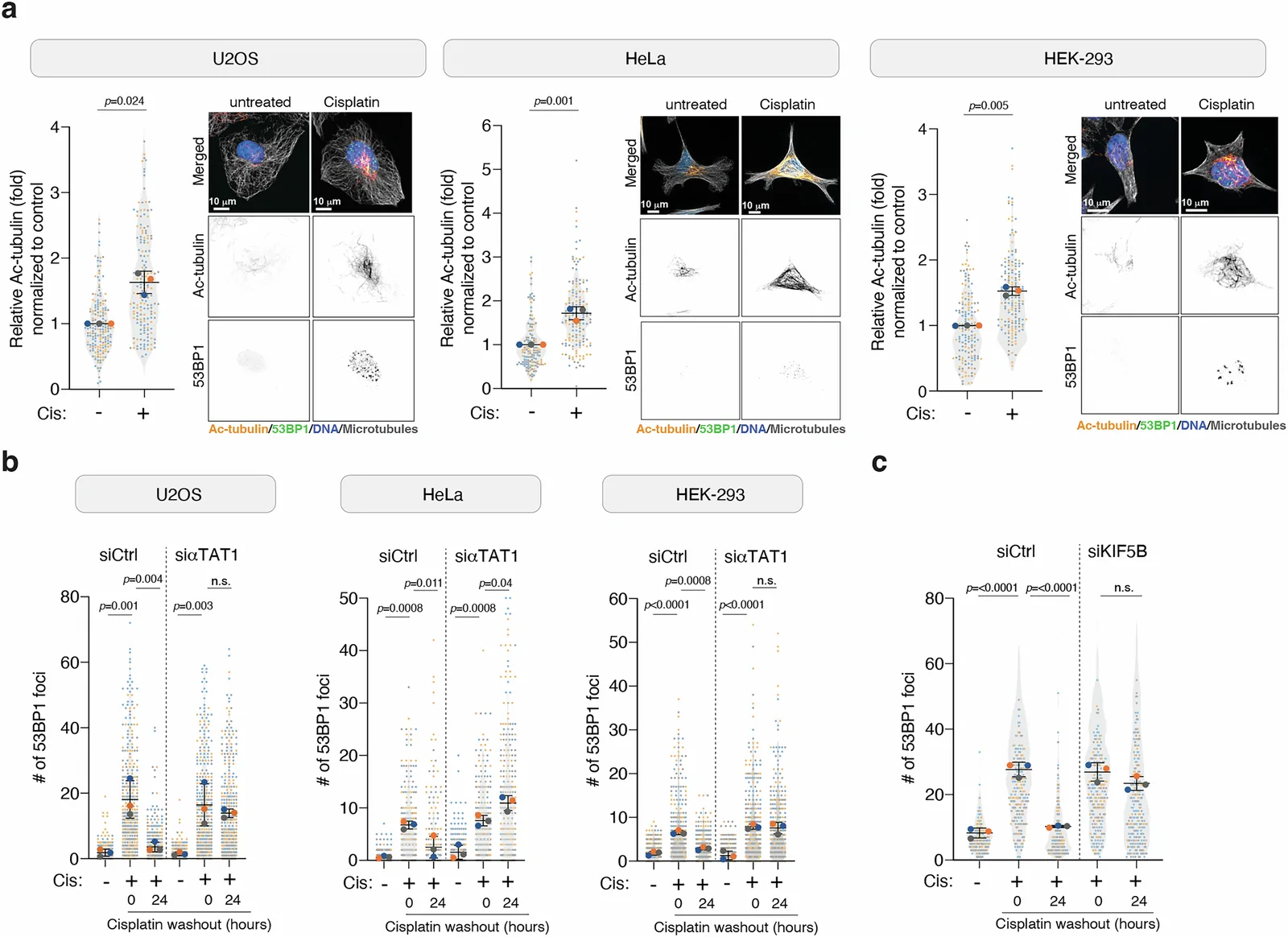
Depletion of αTAT1 prevents efficient DNA repair across multiple cell lines. **a**, Quantification of normalized tubulin acetylation fluorescence intensity to control (right) after cisplatin treatment. Number of cells: U2OS: 178, 172; HeLa: 136, 149; HEK-293: 94, 98 (left to right). Representative images of cells after cisplatin treatment stained for acetylated tubulin (Ac-tubulin, orange), microtubules (α-tubulin, grey), 53BP1 (magenta) and DNA (Hoechst, cyan). Scale bar: 10 µm. **b**, Quantification of 53BP1 foci after cisplatin treatment. Number of cells: U2OS: 337, 326, 320, 328, 333, 325; HeLa: 290, 241, 300, 390, 239, 288; HEK-293: 321, 332, 323, 326, 319, 326 (left to right). **c**, Quantification 53BP1 foci in RPE-1 cells after cisplatin washout upon depletion of KIF5B. Number of cells: n = 197, 197, 207, 208, 221 (left to right). **a,b,c** Error bars represent mean ± SD from three independent experiments. Small dots: all cells analysed per experiment; Large dots: average of cells per experiment. *p* values are described in the graphs and calculated using two-tailed one-sample *t* and Wilcoxon test **a**, and one-way ANOVA for **b**,**c**. All *P* < 0.05 and n.s. = not significant (*P* > 0.05). Source data

In higher eukaryotes, directed movement of DSBs to the nuclear envelope has been shown to play a key role in DNA repair^16^. However, how microtubule-driven localized DSB mobility impacts repair is still unclear. We found that decreasing DSB mobility by blocking tubulin acetylation (using a small interfering RNA (siRNA) designed to silence *αTAT1* expression (siαTAT1)) prevented efficient DNA damage repair, as measured by 53BP1 foci, with no recovery observed 24 h after drug washout in either cisplatin- or doxorubicin-treated cells (Fig. 4a–c). These results were confirmed using a neutral comet assay to more directly assess DSBs (Fig. 4d,e) and validated in additional non-cancer (HEK-293) and cancer cell lines (U2OS and HeLa) (Extended Data Fig. 7a,b). To further assess whether the αTAT1 role in DDR is mediated through tubulin acetylation, we generated two siRNA-resistant αTAT1 variants, one with reduced binding to microtubule protofilaments (αTAT1 F105A) and a catalytically inactive variant (αTAT1 D157E)^44,45^. Whereas siRNA-resistant wild-type αTAT1 expression could rescue the DDR defects in the absence of endogenous αTAT1, neither variant was able to rescue DNA repair induced by αTAT1 depletion (Fig. 4f,g). Thus, both αTAT1 catalytic activity and its ability to bind microtubules are required for efficient DDR.

A key question is whether the link between DSB mobility and DNA repair reflects a direct causal relationship or merely a correlation. To address this, we reasoned that if DSB mobility is required for efficient repair, restoring it through an independent means should be sufficient to rescue DDR in αTAT1-depleted cells. We therefore co-depleted vimentin or plectin alongside αTAT1, which was shown above to restore nuclear fluctuations and DSB mobility independent of tubulin acetylation. Remarkably, this was sufficient to fully rescue DNA damage repair following cisplatin treatment (Fig. 4h–j). These results suggest that tubulin acetylation promotes repair through its role in nuclear fluctuations and DSB mobility. Supporting this conclusion, depletion of KIF5B, required for DSB mobility and nuclear fluctuations, produced comparable DNA repair defects (Extended Data Fig. 7c).

**Fig. 5:**
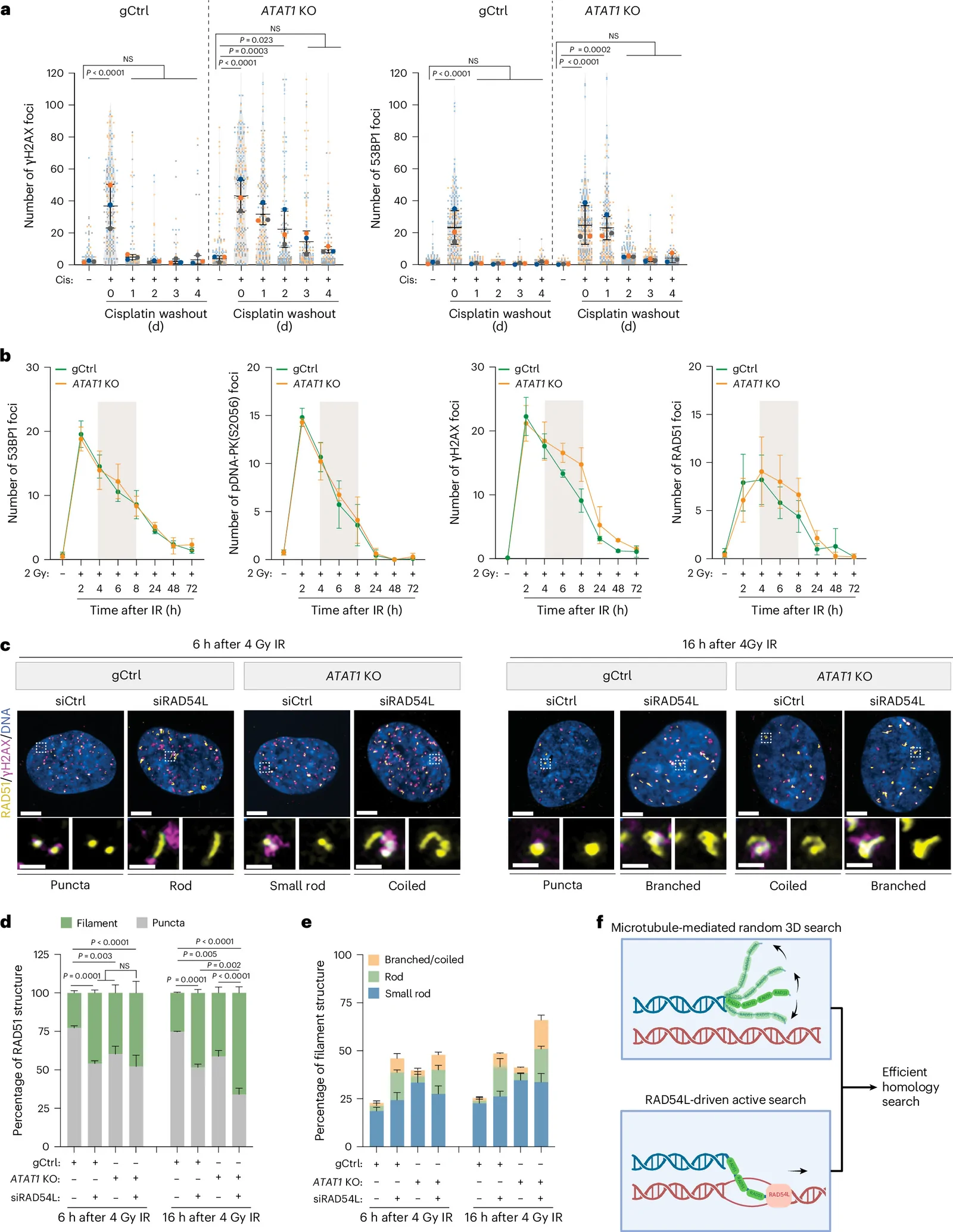
*ATAT1* KO impairs efficient HR. **a**, Quantification of γH2AX foci (left) and 53BP1 foci (right) after cisplatin washout. Numbers of cells (left to right): *n* = 278, 228, 211, 175, 158, 145, 309, 246, 203, 210, 167 and 153. **b**, Dynamics of 53BP1 foci, phospho-DNA-PK foci, γH2AX foci and RAD51 foci after IR. The grey areas represent the time frame when tubulin acetylation and DSB mobility increase. The data are from Extended Data Fig. 8c. **c**, Representative images at 6 h (left) and 16 h (right) after IR in cells stained for RAD51 (yellow), γH2AX (magenta) and DNA (Hoechst; cyan). Main scale bars, 5 µm. Inset scale bars, 1 µm. **d**, Quantification of RAD51 structures (puncta (grey bars) and filaments (green bars)). Numbers of cells (left to right): *n* = 64, 64, 64, 69, 63, 64, 61 and 63; and numbers of foci (left to right): *n* = 2,190, 1,899, 1,672, 2,055, 1,862, 1,830, 1,776 and 1,494. **e**, Distribution of types of RAD51 filament after IR. The data were derived from Fig. 5d. **f**, Schematic showing how the two types of homology search synergize to promote an efficient homology search. In **a**, **b**, **d** and **e**, the data represent means ± s.d. from three independent experiments. In **a**, the small dots represent all cells analysed per experiment and the large dots represent the average of cells per experiment. *P* values are provided in the graphs. Statistical significance in **a** and **d** was determined by two-way ANOVA. Schematic in **f** created in BioRender; Godinho, S. https://biorender.com/dbhhnhy (2026). Source data

**Extended Data Fig. 8:**
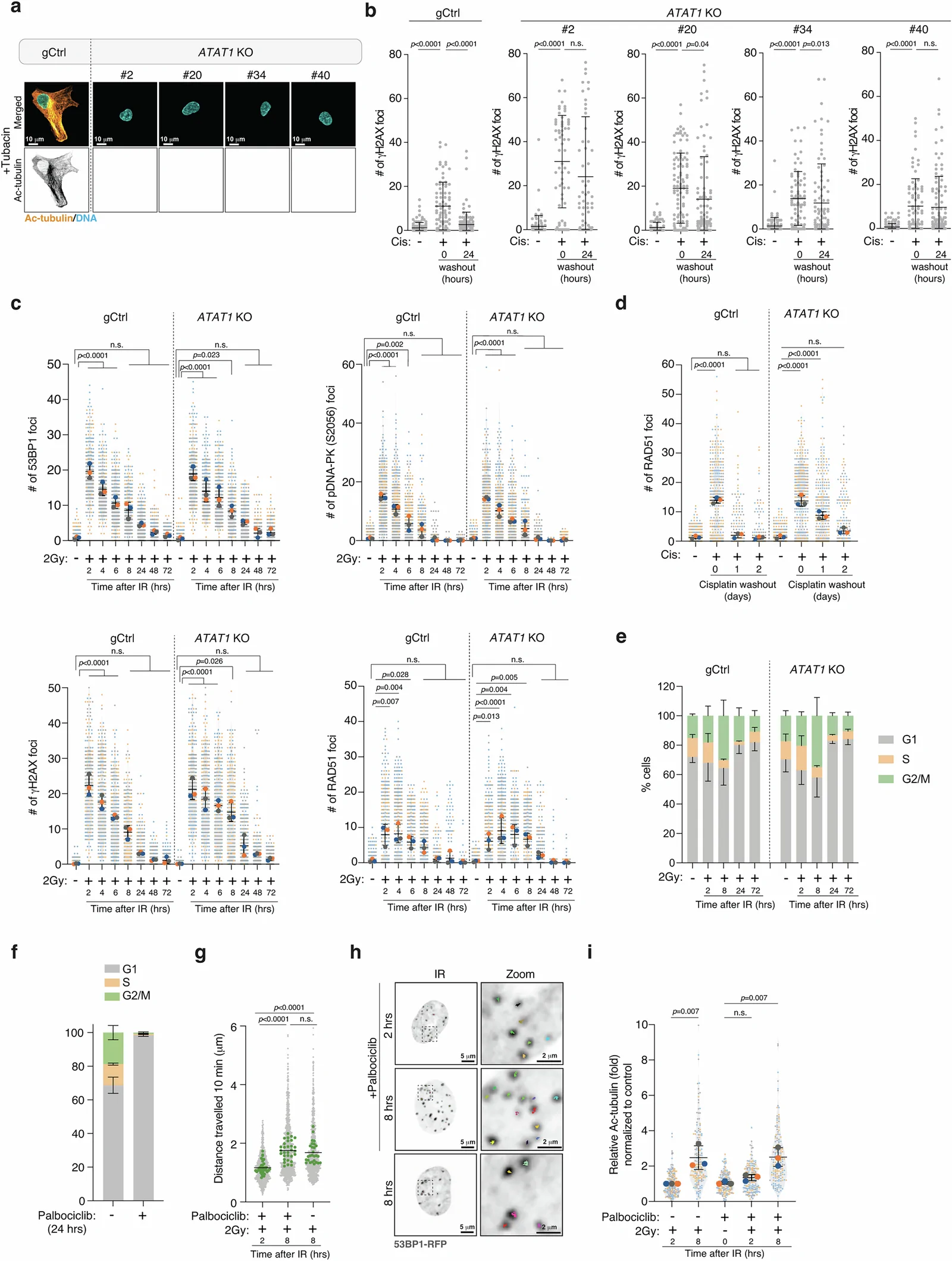
*ATAT1* KO cells display defects in DNA damage repair but not in NHEJ. **a**, Representative images of cells control and *ATAT1* KO clones treated with tubacin and stained for acetylated tubulin (orange) and DNA (Hoechst, cyan). Scale bar: 10 µm. **b**, Quantification of γH2AX foci after cisplatin washout in control and *ATAT1* KO clones. Number of cells gCtrl: n = 103, 84, 112 (left to right), KO#2 n = 89, 65, 63 (left to right), KO#20: n = 92, 105, 93 (left to right), KO#34: n = 99, 81, 99 (left to right), KO#40: n = 119, 84, 106 (left to right). **c**, Quantification of 53BP1 foci (top-left), pDNA-PK(S2056) foci (top-right), γH2AX foci (bottom-letf) and RAD51 foci (bottom-right) after IR. Number of cells (53BP1): n = 325, 322, 290, 304, 315, 318, 277, 253, 358, 345, 319, 355, 325, 331, 308, 309 (left to right); Number of cells (pDNA-PK): n = 389, 351, 347, 388, 331, 336, 348, 334, 358, 338, 332, 352, 321, 335, 343, 326;Number of cells (γH2AX/RAD51): n = 348, 344, 370, 315, 320, 308, 369, 280, 328, 336, 345, 318, 338, 317, 304, 282 (left to right). **d**, Quantification of RAD51 foci after cisplatin washout. Number of cells: n = 377, 356, 372, 387, 341, 378, 372, 357 (left to right). **e**, Percentage of cells at different phases of cell cycle after IR quantified by FACS. **f**, Percentage of cells at different phases of cell cycle after palbociclib quantified by FACS. **g**, 53BP1 foci distance travelled during 10 min in cells after IR and palbociclib-induced G1 arrest. Number of cells/foci=36/812, 30/910, 27/837) (left to right). **h**, Representative still images from live-cell imaging of cells expressing 53BP1-RFP (grey) after IR and palbociclib treatment; lines show movement tracks of individual 53BP1 foci. Scale bar: 5 µm; inset scale bar: 2 µm. **i**, Quantification of normalized tubulin acetylation fluorescence intensity after IR and palbociclib treatment. Number of cells: n = 175, 154, 153, 157, 161 (left to right). **b,c,d,e,f,i** Error bars represent mean ± SD from three independent experiments. **c,d,i** Small dots: all cells analysed per experiment; Large dots: average of cells per experiment. **g** Horizontal bars represent average of 53BP1 foci movement (grey dots) analysed per cell (green dots). *p* values are described in the graphs and calculated using one-way ANOVA with Kruskal-Wallis test for **b,g,h**; one-way ANOVA for **i** and two-way ANOVA for **c,d**. All *P* < 0.05 and n.s. = not significant (*P* > 0.05). Source data

To understand the long-term impact of mobility loss, we generated a CRISPR–Cas9 *ATAT1* knockout (KO) cell line comprising four single KO clones (*ATAT1* KO) that lack K40 α-tubulin acetylation, even after tubacin treatment (Extended Data Fig. 8a). These single-cell clones are also defective in DNA damage repair (Extended Data Fig. 8b). *ATAT1* KO cells showed a marked delay in DSB repair one day after cisplatin washout as assessed by γH2AX and 53BP1, with most 53BP1 foci repaired after two days, whereas the repair of γH2AX foci was slower, with visible persistent damage observed after four days (Fig. 5a). Using IR, we followed DSB repair over time using RAD51 (which labels single-stranded DNA filaments and is a specific marker for HR^1^) and phospho-DNA-PK(S2056) (the phosphorylated form of the DNA-dependent protein kinase (DNA-PK) catalytic subunit at serine residue 2,056, and a marker of active DNA-PK signalling and NHEJ at DSBs^54^), in addition to γH2AX and 53BP1. *ATAT1* KO cells displayed a delay in the repair of γH2AX and RAD51 foci between 6 and 8 h (Fig. 5b and Extended Data Fig. 8c), consistent with the timing of tubulin acetylation and DSB mobility increase under control conditions (Fig. 1f,l). No delay in the resolution of 53BP1 and phospho-DNA-PK(S2056) foci was observed, consistent with these breaks being repaired with faster kinetics by NHEJ^55^ (Fig. 5b and Extended Data Fig. 8c). A significant delay in RAD51 foci resolution was also observed in cisplatin-treated *ATAT1* KO cells (Extended Data Fig. 8d). Together, these data suggest that αTAT1 could play a role in HR rather than NHEJ, which is known to act within the first 6 h after IR^56^, when tubulin acetylation levels are still low. Importantly, these differences are not due to changes in the cell cycle in *ATAT1* KO cells (Extended Data Fig. 8e). Furthermore, DSB mobility is not regulated by the cell-cycle phase. Cells treated with the CDK4/6 inhibitor palbociclib for 24 h^57^ to induce G1 arrest were exposed to IR to induce DSBs (Extended Data Fig. 8f). We found that DSB mobility was low 2 h after IR; however, it increased significantly by 8 h, comparable to that of asynchronous cells, corresponding to the time when increased tubulin acetylation is also observed (Extended Data Fig. 8g–i and Supplementary Video 12). Therefore, tubulin acetylation and DSB mobility appear to be regulated by the timing after DNA damage, rather than the cell-cycle phase.

**Extended Data Fig. 9:**
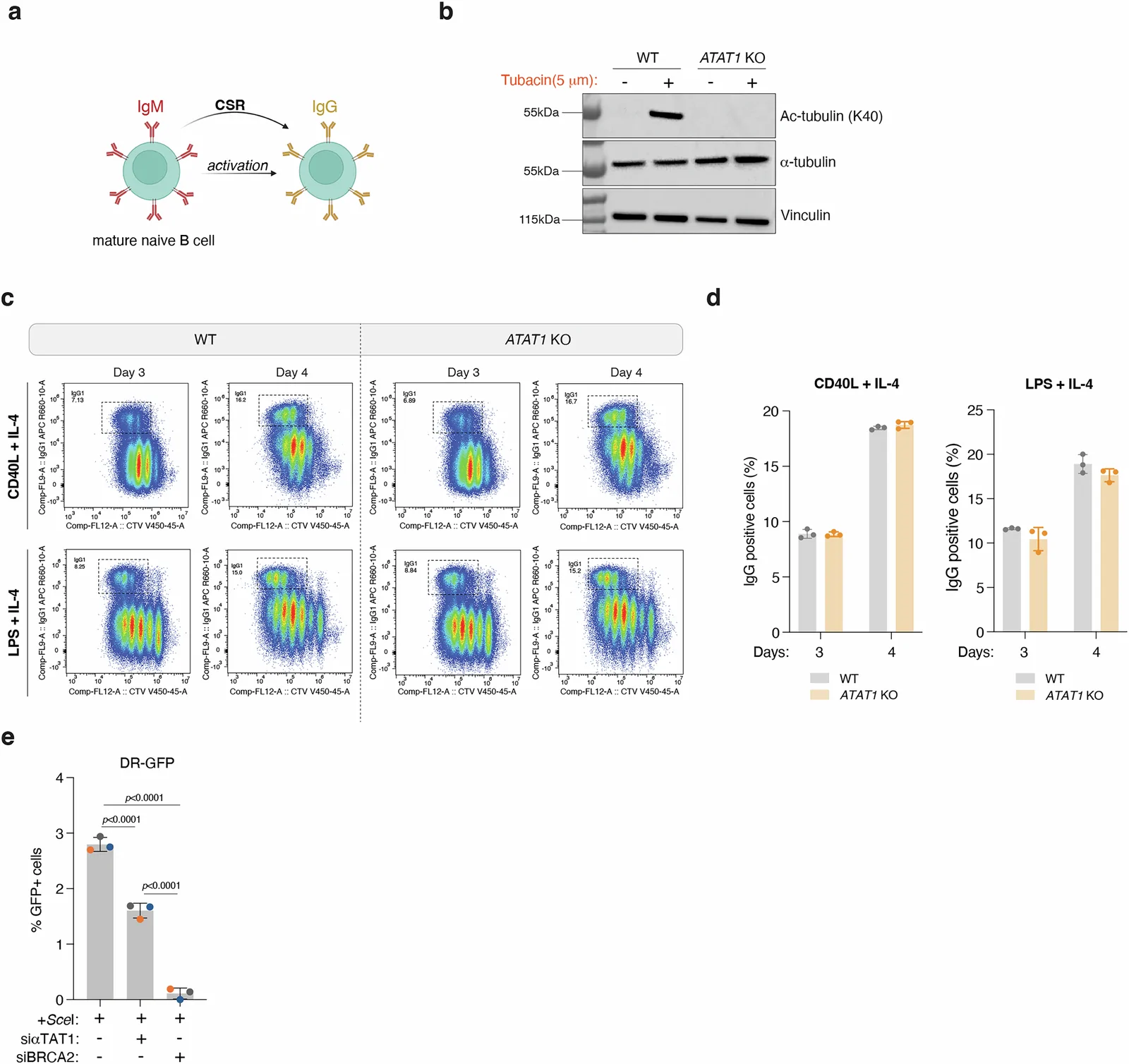
*ATAT1* KO does not impair NHEJ-driven CSR. **a**, Scheme representing CSR in B cells. **b**, Western blot analyses of wild-type and *ATAT1* KO mouse splenocytes with the indicated antibodies with and without tubacin treatment. **c**, FACS analyses of wild-type and *ATAT1* KO mouse splenic B cells after activation with CD40L + IL-4 or LPS + IL-4. Dashed area marks the IgG positive cells. **d**, FACS data quantification of the percentage of IgG positive cells in wild-type and *ATAT1* KO mouse B-cells after activation obtained from three technical replicates. **e**, Percentage of GFP positive cells expressing the HR reporter construct DR-GFP after transfection with *Sce*-I encoding plasmid obtained from three independent experiments. **d,e** Error bars represent mean ± SD. *p* values are described in the graphs and calculated using one-way ANOVA for **e**. All *P* < 0.05. Schematic in **a** created in BioRender; Godinho, S. https://biorender.com/oi2yzml (2026). Source data

To further test the role of αTAT1 in repair pathway specificity, we used well-established assays to monitor HR and NHEJ repair efficiency. For NHEJ, we used class-switch recombination (CSR) in B cells, which changes the antibody isotype (for example, from immunoglobulin M (IgM) to IgG) (Extended Data Fig. 9a)^58–60^. We first confirmed that splenic cells isolated from *ATAT1* KO mice completely lacked K40 α-tubulin acetylation by treating them with tubacin (Extended Data Fig. 9b). Primary B cells isolated from wild-type and *ATAT1* KO mice were stimulated in vitro with either CD40 ligand plus interleukin 4 (IL-4) or lipopolysaccharide (LPS) plus IL-4, to induce CSR, and the expression of switched IgG isotype was determined by fluorescence-activated cell sorting (FACS) (Extended Data Fig. 9c). Quantification of the percentage of B cells expressing IgG at 3 and 4 days after activation revealed that *ATAT1* KO B cells did not display defects in CSR, and thus had efficient NHEJ (Extended Data Fig. 9d). For HR, we used the direct-repeat GFP (DR–GFP) reporter assay to quantify the percentage of GFP^+^ cells following SceI-induced DSBs, for which GFP fluorescence is only restored when the break is accurately repaired by HR using a downstream GFP template^61^. Using HEK-293 cells, due to high transfection efficiency, and BRCA2 depletion as a positive control^62^, we found that, whereas loss of BRCA2 completely abrogates HR, loss of αTAT1 leads to a significant (~40%) reduction in HR (Extended Data Fig. 9e). This is consistent with a role for αTAT1 in HR efficiency rather than activation, as is also supported by the ability of *ATAT1* KO cells to recruit RAD51 to DSBs.

How local DSB mobility promotes efficient HR is unknown. A key difference between HR and NHEJ is that HR requires a search for the homology template, a key rate-limiting step to promote error-free DNA repair^1^. Homology search has been shown to be a highly dynamic process both in yeast and human cells, enabling single-stranded DNA filaments to explore the nuclear space^63^. Using single-molecule imaging, it has been shown that homology search is driven by two types of mobility: random 3D search and active search driven by the ATPase motor RAD54L, which is responsible for strand invasion^64^. We postulated that tubulin acetylation could regulate the random 3D search wiggly movement, thus cooperating with RAD54L to drive efficient homology search. To test this idea, we assessed RAD51 foci morphology, particularly the presence of filamentous structures, as this has been shown to correlate with defects in homology search in both yeast and human cells^65,66^. In human cells treated with IR, increased RAD51 filaments (rod or small rod) are associated with delayed homology search, whereas complex structures (coiled or branched) are associated with severe defects caused by RAD54L loss (Fig. 5c). Compared with RAD54L depletion alone, we observed that *ATAT1* KO cells displayed a similar increase in the percentage of RAD51 filaments at both 6 and 16 h after IR (~45–50% compared with ~25% for the control; Fig. 5d). Interestingly, when we examined the different types of RAD51 structures, *ATAT1* KO predominantly led to rod or small rod shapes compared with the more complex branched and coiled shapes generated by RAD54L depletion (Fig. 5c–e). Importantly, upon the loss of both RAD54L and αTAT1 16 h after IR, when defects due to homology search delays are most notable^66^, we observed both an increase in overall RAD51 filaments (~70%) and significantly increased complex branched or coiled structures (Fig. 5c–e). Collectively, these findings indicate that αTAT1 facilitates DSB repair via HR through promoting random 3D search that cooperates with RAD54L-driven homology search (Fig. 5f).

### Unrepaired DSBs form large clusters and promote permanent cell-cycle arrest

**Fig. 6:**
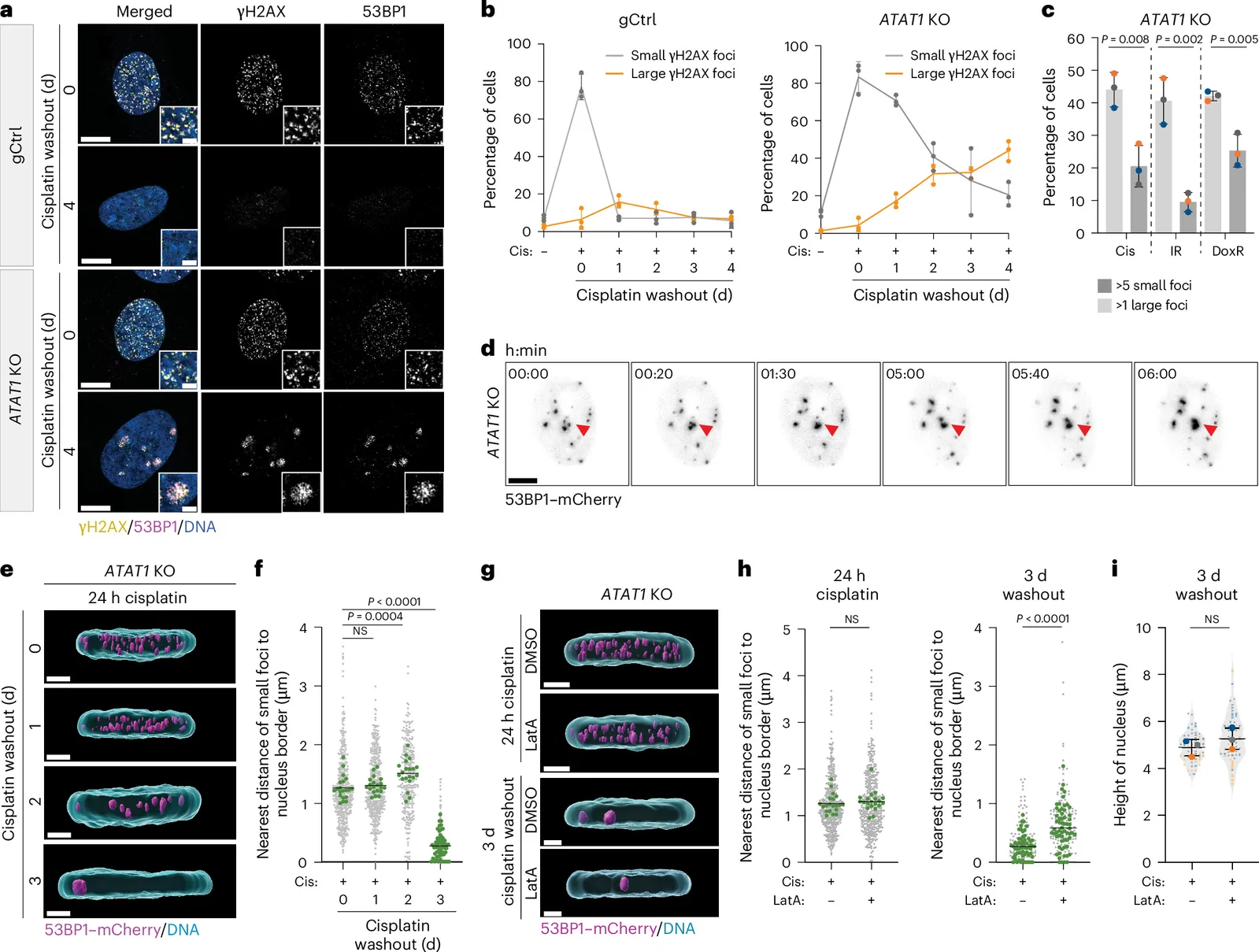
Unrepairable DSBs form large clusters and promote cell-cycle arrest in *ATAT1* KO cells. **a**, Representative images of cells after cisplatin washout stained for 53BP1 (magenta), γH2AX (yellow) and DNA (Hoechst; cyan). Main scale bars, 10 µm. Inset scale bars, 2 µm. **b**, Percentages of cells with small and large γH2AX foci in control and *ATAT1* KO cells after cisplatin washout. Numbers of cells (left to right) for gCtrl: *n* = 294, 235, 225, 175, 165 and 152; and numbers of cells (left to right) for *ATAT1* KO: *n* = 313, 259, 196, 195, 166 and 150. **c**, Percentages of *ATAT1* KO cells with small and large foci 4 days after cisplatin and doxorubicin washout and 3 days after IR. Numbers of cells (left to right): *n* = 150, 246 and 382. **d**, Representative still images from live-cell imaging of *ATAT1* KO cells expressing 53BP1–mCherry 2 days after cisplatin washout. The red arrows mark the clustering of 53BP1 foci. Scale bar, 10 µm. **e**, 3D surface rendering of nuclei (cyan) in live *ATAT1* KO cells expressing 53BP1–mCherry (magenta) after cisplatin treatment and washout. Scale bars, 5 µm. **f**, Quantification of the nearest distance of small foci to the nucleus border in *ATAT1* KO cells treated with cisplatin and after washout. Numbers of cells (left to right): *n* = 20, 20, 20 and 61; and numbers of foci (left to right)**:** *n* = 439, 439, 295 and 152. **g**, 3D surface rendering of nuclei (cyan) in live *ATAT1* KO cells expressing 53BP1–mCherry (magenta) after treatment with cisplatin and latrunculin A (LatA). Scale bars, 5 µm. **h**, Quantification of the nearest distance of small foci to the nucleus border in *ATAT1* KO cells treated with cisplatin and LatA before washout (left) and 3 days after washout (right). In the left graph, numbers of cells (left to right): *n* = 20 and 20; and numbers of foci (left to right): *n* = 439 and 411. In the right graph, numbers of cells (left to right): *n* = 20 and 20; and numbers of foci (left to right) *n* = 439 and 295. **i**, Quantification of nucleus height in cells treated with cisplatin and LatA after washout. Number of cells: *n* = 62 per condition. In **b**, **c** and **i**, the data represent means ± s.d. from three independent experiments and the dots represent averages per experiment. In **f** and **h**, the horizontal bars represent the average of 53BP1 foci (grey dots) analysed per cell (green dots) from three independent experiments. In **i**, the small dots represent all cells analysed per experiment and the large dots represent the average of cells per experiment. *P* values are provided in the graphs. Statistical significance was determined by two-tailed unpaired *t*-test (**c** (treatments were preformed independently), **h**(right) and **i**), one-way ANOVA (**f**) or two-tailed Mann–Whitney *U* test (**h**(left)). Source data

Monitoring DNA damage repair over longer periods of time revealed that, whereas small DSB foci decreased over time, a higher proportion of *ATAT1* KO cells accumulated large foci over time (size cut-off: >1.75 μm^2^; considerably bigger than small foci: ~0.2–0.5 μm^2^), reaching ~40% of cells at day 4 after cisplatin washout (Fig. 6a,b). This phenotype was consistent across different genotoxic agents (Fig. 6c). Live-cell imaging of *ATAT1* KO cells expressing 53BP1–mCherry showed that persistent DSBs can cluster and coalesce, forming large foci of unrepaired breaks (Fig. 6d and Supplementary Video 13). When assessing the spatial distribution of foci within the nucleus in *ATAT1* KO cells, we found that the overall distribution of foci did not differ from that of control cells after cisplatin treatment (Fig. 1n,o) or one day after washout (Fig. 6e,f and Supplementary Videos 14 and 15), when no significant repair was observed in the *ATAT1* KO cells (Fig. 5a). However, a significant shift occurred by day 2, at a point when a subset of foci had been repaired, and the remaining persistent foci were located further from the nuclear envelope (Fig. 6e,f and Supplementary Video 16). Strikingly, and consistent with previous findings in yeast^67,68^, large foci containing unrepaired DSBs (3–4 days after cisplatin washout) preferentially localized near the nuclear envelope, with an average distance of ~0.2 µm between the foci border and the nuclear periphery (Fig. 6e,f and Supplementary Video 17). This re-localization does not require acetylated microtubules and is probably a result of directed transport inside the nucleus, which is driven by actin and associated myosin motors^15,69^. In agreement, actin depolymerization with latrunculin A prevented efficient re-localization of large foci to the nuclear envelope, without affecting the overall distribution of small DSBs immediately after cisplatin treatment, or nuclei height (Fig.6g–i and Supplementary Videos 18–21). Thus, localized DSB mobility during repair is mainly promoted by cytoplasmic microtubules and requires tubulin acetylation, whereas the directed transport of large unrepaired DSB foci is driven by the actin cytoskeleton.

**Fig. 7:**
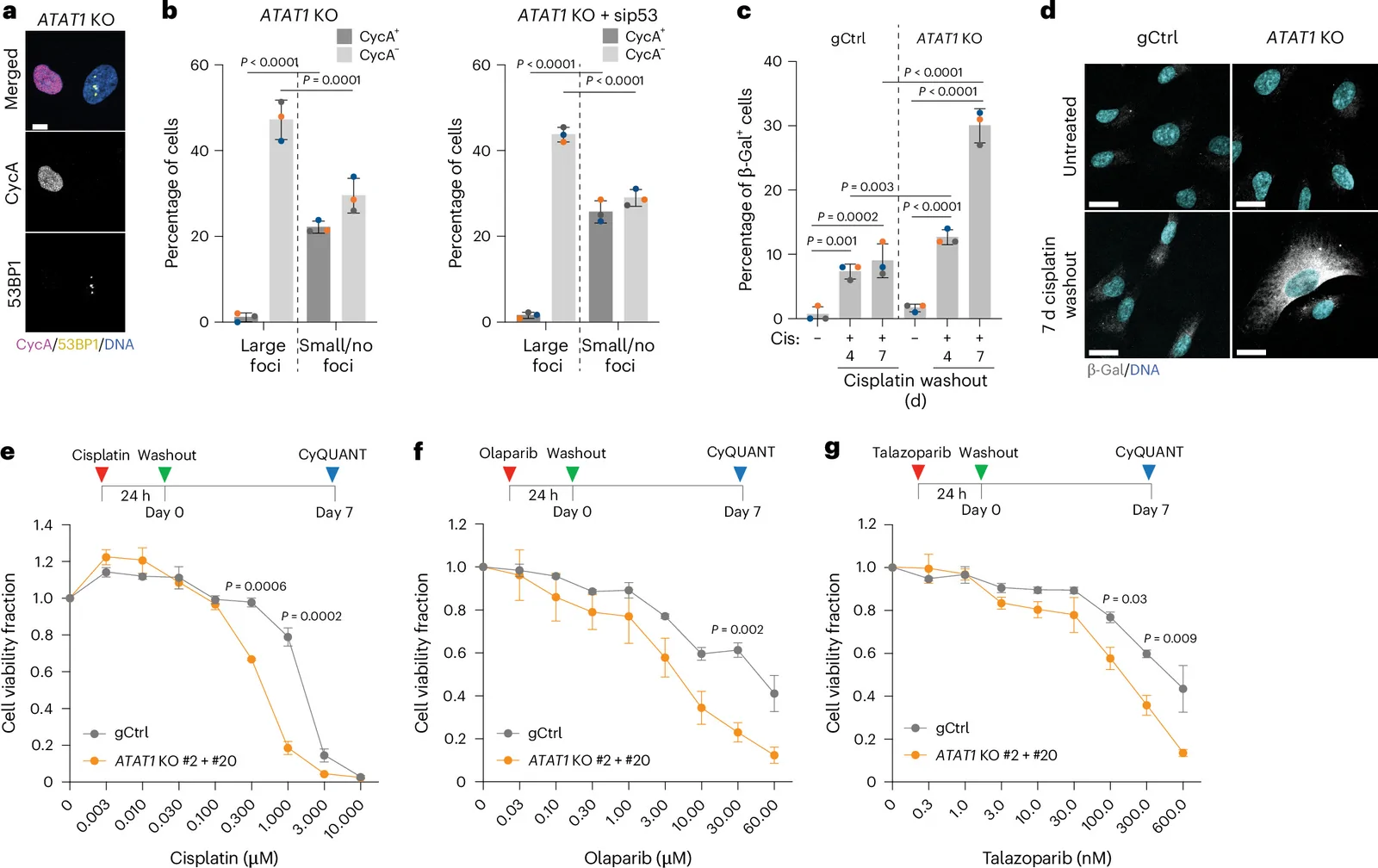
DDR defects in *ATAT1* KO cells lead to cell-cycle arrest, senescence and loss of viability. **a**, Representative images of cells stained for cyclin A (CycA; magenta), 53BP1 (yellow) and DNA (Hoechst; cyan). Scale bar, 10 µm. **b**, Percentage of CycA^+^ and CycA^−^ *ATAT1* KO cells 4 days after cisplatin washout. Number of cells: *n* = 412 for *ATAT1* KO; and *n* = 370 for *ATAT1* KO + sip53. **c**, Percentages of senescence cells (β-galactosidase (β-gal) positive) in *ATAT1* KO cells 4 and 7 days after cisplatin washout. Number of cells: *n* = 300 per condition. **d**, Representative images of live cells stained for β-galactosidase (grey) and DNA (Hoechst; cyan). Scale bars, 20 µm. **e**–**g**, Quantification of the cell viability fraction 7 days after cisplatin (**e**), olaparib (**f**) or talazoparib treatment (**g**). In **b** and **c**, the data represent means ± s.d. from three independent experiments and the dots represent averages per experiment. In **e**–**g**, the dots represent means ± s.d. from three independent experiments. *P* values are provided in the graphs. Statistical significance was determined by two-way ANOVA (**b** and **c**) or two-tailed unpaired *t*-test for (**e–g**). Source data

The formation of large foci in *ATAT1* KO cells compromised cell proliferation. Using immunofluorescence of cyclin A as a marker of proliferation we found that 4 days after cisplatin washout *ATAT1* KO cells containing large foci (usually one to four) were arrested in the G1 phase (that is, they were cyclin A negative), which was not affected by p53 loss, whereas cells with small or no foci could still proliferate (cyclin A positive) (Fig. 7a,b). Moreover, 7 days after cisplatin treatment, *ATAT1* KO cells displayed a significant increase in senescence induction (~30%), as quantified using β-galactosidase staining (Fig. 7c,d).

**Extended Data Fig. 10:**
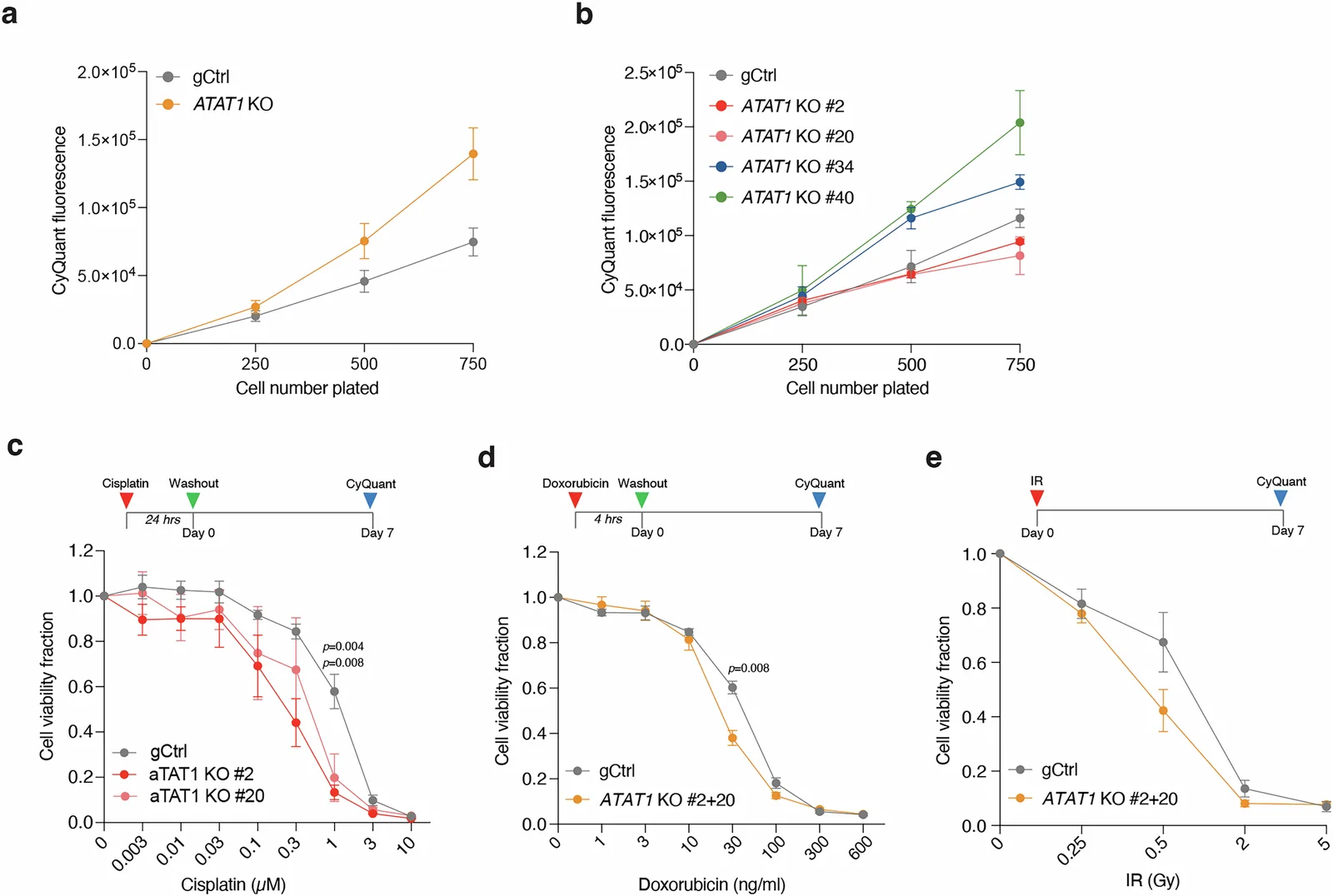
*ATAT1* KO cells are more sensitive to DNA damage drugs. **a**, CyQUANT fluorescent intensity analyses of gCtrl and *ATAT1* KO cells plated at different densities and grown for 4 days. **b**, CyQUANT fluorescent intensity analyses of gCtrl and deconvolved four *ATAT1* KO clones plated at different densities and grown for 4 days. **c**, Quantification of the cell viability fraction 7 days after cisplatin treatment and washout. **d**, Quantification of the cell viability fraction 7 days after Doxorubicin treatment and washout. **e**, Quantification of the cell viability fraction 7 days after IR. For all graphics Dots represent mean ± SD obtained from three independent experiments. *p* values are described in the graphs and calculated using two-tailed unpaired t-test for **c,d,e**. All *P* < 0.05. Source data

To test whether impaired HR efficiency in *ATAT1* KO cells increases sensitivity to DNA-damaging agents, we used the CyQUANT DNA content-based assay as a surrogate measure of cell number, which is ideally suited when the expected outcome is prolonged cell-cycle arrest rather than cell death^70^. Because the original mixed *ATAT1* KO population grew faster than non-targeting control cells (gCtrl) cells (Extended Data Fig. 10a), masking drug-induced proliferation defects, we generated a new population comprising only clones #2 and #20, which displayed growth rates comparable to those of gCtrl cells (Extended Data Fig. 10b). Cells were treated with single doses of DNA-damaging agents and proliferation was monitored at day 7 following drug washout. Agents for which cytotoxicity depends on HR, such as the poly-ADP ribose polymerase inhibitors olaparib and talazoparib, and cisplatin^71–73^, caused the greatest differential sensitivity, reducing proliferation by ~40 and ~30%, respectively, in *ATAT1* KO cells (Fig. 7e–g and Extended Data Fig. 10c). Doxorubicin, which generates DSBs that can be repaired by both HR and NHEJ^74^, had a milder effect (~20% reduction), and IR, whose DSBs rely predominantly on NHEJ for repair^75^, showed a more subtle trend towards increased sensitivity at 0.5 Gy (Extended Data Fig. 10d,e). Changes in viability were only observed at day 7, consistent with increased senescence at this time point. These results are consistent with a partial defect in *ATAT1* KO cells and support the conclusion that tubulin acetylation-mediated DSB mobility contributes to efficient HR repair.

## Discussion

**Fig. 8:**
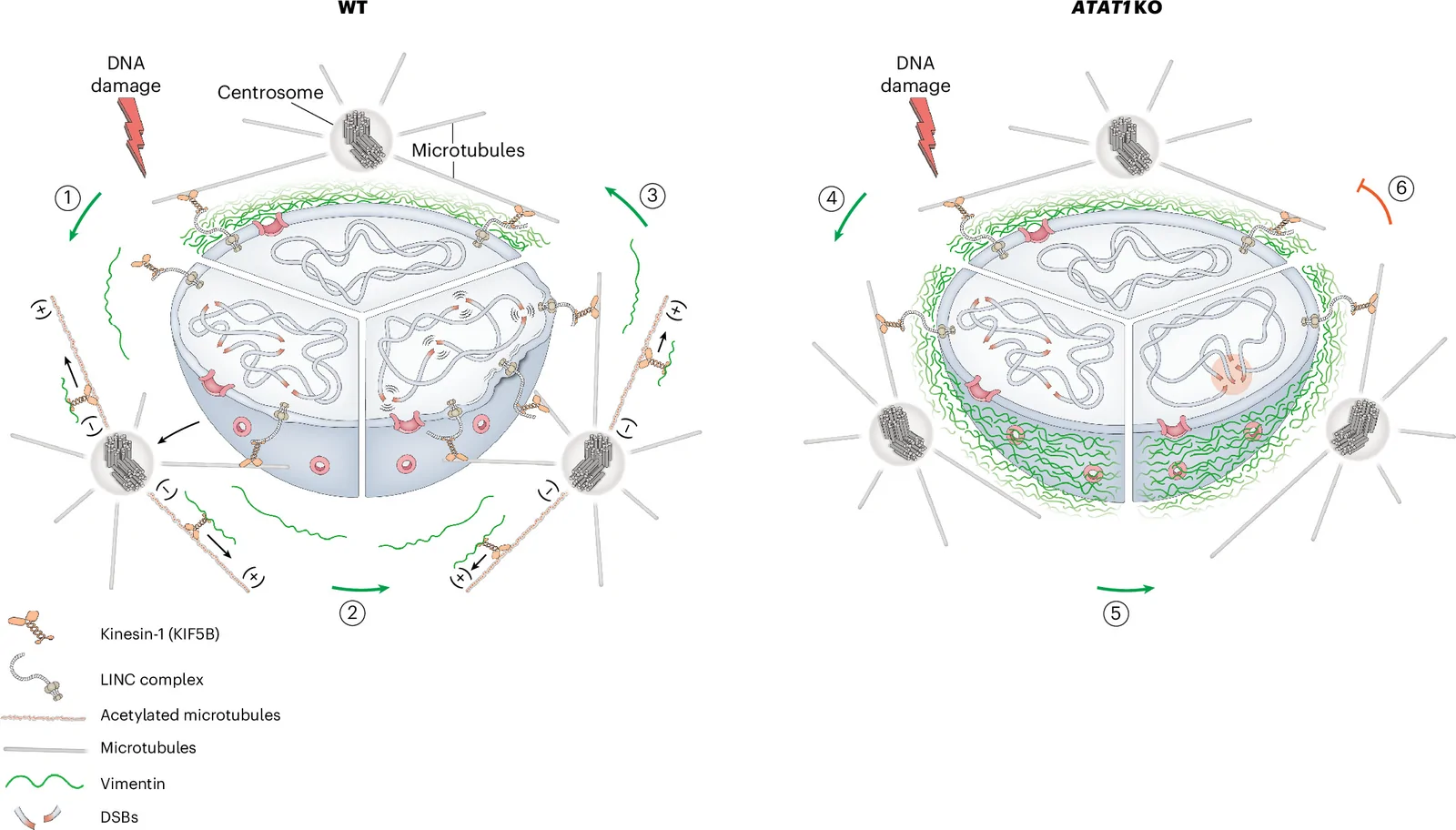
Model of how tubulin acetylation promotes DNA repair. In wild-type cells (left): (1) DNA damage increases microtubule acetylation, which promotes the transport of vimentin away from the nuclear envelop via KIF5B; (2) this leads to a softer nucleus, microtubule-driven nuclear fluctuations and DSB mobility; and (3) DSB mobility ensures efficient HR-mediated DNA repair, after which cells return to a pre-damage state. In the absence of αTAT1 and tubulin acetylation (*ATAT1* KO; right): (4) vimentin displacement is prevented; (5) this blocks microtubule-driven nuclear fluctuations and DSB mobility; and (6) outcomes of this include defects in DNA repair, the formation of DSB clusters and cell-cycle arrest. Credit: Reza Roozitalab.

Our findings show that nuclear shape fluctuations represent a controlled mechanical response during DNA DSB repair. We show that DNA damage induces α-tubulin acetylation via αTAT1, which displaces the perinuclear vimentin cage, exposing the nucleus to forces mediated by centrosome-nucleated microtubules. These forces deform the nuclear envelope and generate transient fluctuations (Fig. 8). We propose that these changes in nuclear mechanics enhance the local sub-diffusive mobility of DSBs, increasing the nuclear space that DSBs explore during homology search and thereby promoting efficient HR. Although our data define a mechanism by which microtubule forces drive nuclear envelope deformation, how these forces propagate to chromatin and modulate DSB mobility remains an important open question.

Changes in nuclear mechanical properties to allow microtubules to deform the nucleus probably depend on cellular context. Mitotic slippage produces tetraploid cells with softer nuclei than diploid counterparts, permitting microtubule-driven invaginations^76^. Nuclear deformability also varies with differentiation state^77^ and thus could influence how cells respond to microtubule forces. Indeed, during haematopoietic stem cell differentiation, microtubules drive nuclear invaginations in myeloid progenitors, promoting local heterochromatin loss and supporting lineage-specific gene expression programs^78^. Here we propose that the reversible displacement of vimentin leads to nuclear softening during the DDR, enabling microtubule-driven nuclear fluctuations—a mechanism that could allow cells to adapt to different cellular contexts. This is particularly relevant in the context of cancer metastasis, where vimentin reorganization is a hallmark of epithelial-to-mesenchymal transition and associated with increased metastatic potential of cancer cells^79,80^. Although vimentin displacement promotes nuclear softness and could facilitate invasion through dense extracellular matrices, as suggested by our previous work^25^, a stiffer nucleus enclosed by a vimentin cage could protect metastatic cells against shear stress in the bloodstream^81^. Moreover, vimentin loss leads to DNA damage during confined migration, chromatin remodelling and altered gene expression^49,82,83^. Thus, vimentin displacement could allow the tuning of nuclear mechanics without the long-term consequences of vimentin loss.

The mechanisms that lead to increased tubulin acetylation in response to DNA damage remain unclear. One possibility is that DNA-damaging agents act directly on microtubules, as was previously observed for cisplatin^84^ and doxorubicin^85^. Although we did not observe a loss of microtubule polymer under our conditions, we cannot rule out that changes in microtubule dynamics or lattice damage, such as those caused by elevated reactive oxygen species^86^, could play a direct role in driving elevated tubulin acetylation.

Different types of DSB mobility have been described, including microtubule-driven localized 3D mobility and actin-mediated directed transport inside the nucleus^5,8^. Our work shows that different types of mobility can co-exist during the DDR and are temporally regulated. Microtubule-driven wiggly motion emerges several hours after damage (∼8 h after IR) and is regulated by the rise in tubulin acetylation. This temporal regulation could play an important role in repair. NHEJ promotes repair at earlier times after IR^56^ and relies on proximity-based end bridging, thus excessive mobility could disrupt repair by promoting aberrant fusions. This is consistent with previous work showing that NHEJ-mediated aberrant chromosome fusions require DSB mobility^13^. In parallel, and similarly to previous observations^16^, a distinct actin-dependent pathway mobilizes clusters of persistent breaks in αTAT1-deficient cells to the nuclear periphery. However, unlike heterochromatin breaks^15^, this re-localization fails to promote repair and instead drives cell-cycle exit and senescence, even in the absence of p53.

How does microtubule-driven localized DSB mobility facilitate repair? We show that although NHEJ occurs normally in cells with reduced DSB mobility, HR is partly compromised, suggesting a role in HR efficiency, rather than activation. Indeed, efficient HR relies on homology search and thus benefits from enhanced local exploration of nuclear space^66^. Motivated by previous work^64^, we found that microtubule-driven local DSB mobility cooperates with RAD54L-driven active search to promote efficient HR. Simultaneous loss of both αTAT1 and RAD54L caused a synergistic increase in branched and coiled RAD51 filaments, indicative of a severely defective homology search^66^. These data support a direct role for localized DSB mobility in repair efficiency. This is further supported by rescue experiments demonstrating that co-depletion of vimentin or plectin in cells without acetylated tubulin to restore nuclear fluctuations and DSB mobility was sufficient to rescue the DNA repair defects observed in these cells.

Impairing DSB mobility prevents the repair of a subset of DSBs, suggesting that not all breaks rely equally on mobility for resolution. Our data indicate that these persistent foci are initially positioned further away from the nuclear envelope, before clustering and moving towards the nuclear periphery. Although we do not know the basis of this positional shift, it raises the possibility that the genomic context of these breaks influences both their repairability and their reliance on mobility. One possibility is that DSB position can directly impact repair pathway choice. Indeed, DSBs in lamina-associated domains are repaired by alternative end joining rather than HR^87^. Additionally, chromatin conformation and local compaction state, which are increasingly recognized as important determinants of repair kinetics and pathway choice^88^, could also explain this positional shift, with more open euchromatin associated with the nuclear interior preferentially being repaired by HR and therefore likely to require DSB mobility^89,90^. Alternatively, DSBs that are not resolved when mobility is impaired could arise in genomic regions that are inherently more difficult to repair, such as highly transcribed loci. These loci tend to occupy more central nuclear positions and can present challenges for HR due to the need to displace the transcriptional machinery and resolve R-loops^91^. Determining which genomic features distinguish the foci that persist in the absence of αTAT1-driven mobility will be key to understanding how genomic context dictates the spatial requirements for DSB repair.

Our findings that αTAT1 loss sensitizes cells to HR-dependent DNA-damaging agents raise the possibility that modulating tubulin acetylation or DSB mobility could enhance the efficacy of existing chemotherapies. As targeted agents that selectively inhibit DSB mobility become tractable, our framework integrating cytoskeleton mechanisms into DNA repair may open new opportunities to exploit this pathway in cancer treatment.

## Methods

### Cell culture

RPE-1 (human retinal epithelial) cells were cultured in a combination of Dulbecco’s modified Eagle’s medium (DMEM) and Ham’s Nutrient Mixture F-12 (Sigma–Aldrich) supplemented with 10% foetal bovine serum (FBS; Gibco) and 100 U ml^−1^ penicillin–streptomycin (Gibco). The RPE-1 cell line was obtained from the Pellman laboratory at the Dana-Farber Cancer Institute and short tandem repeat profiled by us. U2OS (human osteosarcoma epithelial), HeLa (human cervical carcinoma epithelial) and HEK-293M (human embryonic kidney) cells were grown in DMEM (Sigma–Aldrich) supplemented with 10% FBS and 100 U ml^−1^ penicillin–streptomycin. The U2OS and HeLa cell lines were obtained from the laboratories of S. Martin and R. Bellelli, respectively, both at the Barts Cancer Institute at the Queen Mary University of London. The HEK-293M cell was obtained from the Pellman laboratory at the Dana-Farber Cancer Institute. The U2OS, HeLa and HEK-293M cell lines were not authenticated. All cells were maintained at 37 °C under a 5% CO_2_ atmosphere. All cells used were routinely inspected for *Mycoplasma* contamination. The FBS was heat inactivated in a 56 °C water bath for 30 min before use.

### Generation of *ATAT1* CRISPR–Cas9 KO cells

RPE-1 gCtrl and RPE-1 *ATAT1* KO cell lines were generated using the Dharmacon Edit-R CRISPR–Cas9 Gene Engineering System. Briefly, RPE-1 cells were co-transfected with enhanced GFP (EGFP) Cas9 nuclease messenger RNA (CAS11860; Dharmacon), Edit-R *trans*-activating CRISPR RNA (U-002005-05; Horizon Discovery) and one of two CRISPR RNAs designed to target *ATAT1* (CM-014510-01-0002 or CM-014510-02-0002; Dharmacon), or a non-targeting control CRISPR RNA (CM-014510-01-0002; Dharmacon) using Lipofectamine 2000 (Thermo Fisher Scientific). Cells were expanded and subsequently seeded as single cells on a 96-well plate by serial dilution (0.5 cells per well). *ATAT1* KO clones were verified by immunofluorescence of acetylated tubulin following treatment with the HDAC6 inhibitor tubacin (5 µM; 5 h) to promote microtubule acetylation. Four individual *ATAT1* KO clones (from two independent guides) were pooled to establish a polyclonal *ATAT1* KO cell line.

### Plasmids and cell lines

RPE-1 cells expressing 53BP1–RFP and H2B–GFP were a kind gift from the group of L. Stojic (Barts Cancer Institute), originally made by the group of R. Basto (Institut Curie)^92^. RPE-1 αTAT1-overexpressing cell lines were generated using pLV–EGFP–αTAT1 plasmid and selected by FACS^25^. pLV–EGFP–αTAT1 was prepared from pEF5B–FRT–GFP–αTAT1 (27099; Addgene)^22^ by PCR with BamHI and SalI restriction sites on the 5′ and 3′ end, respectively, and cloned into pLV–EGFP (36083; Addgene). RPE-1 centrin1–GFP cells were previously generated^93^. The RPE-1 gCtrl 53BP1–mCherry and *ATAT1* KO 53BP1–mCherry cell lines were generated using a pLenti6–mCherry–53BP1c lentiviral vector^94^ (a kind gift from G. Hewitt at King’s College London) and selected by FACS.

To generate RPE-1 αTAT1 siRNA-resistant cell lines, we first generated two overlapping PCR fragments containing the siRNA-resistant mutation site, using pLV–EGFP–αTAT1 as a template and Q5 High-Fidelity DNA Polymerase (New England Biolabs). The first fragment was amplified with BamHI–EGFP forward and siRNA-resistant αTAT1 wild-type reverse primers; the second was amplified with siRNA-resistant αTAT1 wild-type forward and SalI-αTat reverse primers. Equal amounts of each purified fragment were combined and assembled by overlap–extension PCR using BamHI–EGFP forward and SalI-αTat reverse primers. The purified product was cloned into pCR-Blunt II-TOPO (Zero Blunt TOPO PCR Cloning Kit; Thermo Fisher Scientific) per the manufacturer’s protocol, and positive clones were confirmed by Sanger sequencing. The verified product was then cloned into pLV–EGFP with BamHI or SalI digestion (New England Biolabs) and T4 DNA ligation (New England Biolabs), yielding siRNA-resistant pLV–EGFP–αTAT1. The ligation product was transformed into One Shot Stbl3 Chemically Competent *E. coli* (Thermo Fisher Scientific).

To generate the RPE-1 αTAT1 F105A and D157E siRNA-resistant cell lines, site-directed mutagenesis was performed using the siRNA-resistant pLV–EGFP–αTAT1 plasmid as a template. Mutations were introduced using primers specific for αTAT1 F105A (αTAT1 F105A) and αTAT1 D157E (si9-Res-aTat-D157E), respectively, following the same PCR-based strategy described above (Supplementary Table 1).

### Mice

*Atat1*^tm1(KOMP)Vlcg^ (*ATAT1* KO) C57BL6 mice (Research Resource Identifier (RRID): MMRRC_046712-UCD) were obtained from the Mutant Mouse Resource and Research Center at the University of California, Davis (UC Davis)—a strain repository funded by the National Institutes of Health—and was donated to the Mutant Mouse Resource and Research Center by the KOMP Repository at UC Davis, originating from K. Lloyd at the UC Davis Mouse Biology Program^95–97^. All mice were bred and maintained under specific-pathogen-free conditions at the Francis Crick Institute’s Biological Research Facility, in accordance with UK Home Office guidelines, and approved by the Francis Crick Institute’s ethical review panel. Animals were housed at 22 ± 2 °C and under 55 ± 10% relative humidity on a cycle of 12 h light and 12 h dark, with the light intensity at 70% and a 15-min gradual ramp up and down at each transition.

### Lentiviral generation and transduction

To generate lentivirus, HEK-293M cells were seeded in a six-well plate with antibiotic-free growth media until they reached ~80% confluency. Transfection of 2 µg of the appropriate lentiviral plasmid in combination with 1 µg Gag-Pol (psPAX2; plasmid 12260; Addgene) and 0.5 µg vesicular stomatitis virus G protein (pMD2.G; plasmid 12259; Addgene) was performed using Lipofectamine 2000, per the manufacturer’s specifications. For each well, the transfection solution was prepared as follows: 10 µl Lipofectamine 2000 Transfection Reagent was diluted in 250 µl Reduced Serum Medium Opti-MEM (Thermo Fisher Scientific) in a sterile 1.5-ml microcentrifuge tube, then 2 µg of the appropriate lentiviral plasmid DNA, 1 µg Gag-Pol and 0.5 µg vesicular stomatitis virus G protein were diluted in 250 µl Opti-MEM in a separate sterile 1.5-ml microcentrifuge tube. Tubes were then incubated at room temperature for 5 min for equilibration. Opti-MEM solution containing plasmid DNA was then added dropwise onto the tube containing the Lipofectamine 2000 solution and incubated for 20 min at room temperature to allow liposome formation. The solution was then added dropwise onto the six-well plate and incubated for 6 h. After 6 h, the transfection mixture was removed and 1.5 ml growth media was added. The resultant medium containing lentivirus was harvested 24 and 48 h after infection and centrifuged at 270*g* for 3 min. The supernatant was filtered through a 0.45-µm syringe filter and stored in cryovials at −80 °C. For infection, RPE-1 cells were seeded in a six-well plate at ~60% confluency. Before infection, the growth medium was replaced with 1 ml fresh growth medium without antibiotics. Then, 1 ml of the appropriate lentivirus was mixed with 10 μg ml^−1^ Polybrene Infection/Transfection Reagent (Sigma–Aldrich) before this was added to the cells in a dropwise fashion. Infection was repeated the following day. Antibiotic selection started 24 h after final infection, or cells were selected by FACS with BD Biosciences FACSAria II or Fusion 7 days after the final infection.

### Chemicals and irradiation

Chemical and irradiation treatments were performed as follows. We added either 2 µM cisplatin (Sigma–Aldrich) for 24 h (to induce DSBs indirectly through the formation of intra-strand and inter-strand crosslinks) or 200 ng ml^−1^ doxorubicin hydrochloride (Sigma–Aldrich) for 4 or 24 h (to induce DSBs more directly via the inhibition of topoisomerase II). Where indicated, drug washout was performed after 24 h cisplatin or doxorubicin treatment by washing cells four times with culture media. Irradiation to induce fast and direct DSBs was performed with an X-ray biological irradiator at the Barts Cancer Institute. We also treated cells with: 0.1 µM latrunculin A (Sigma–Aldrich) for 24 h to depolymerize F-actin; 2 µM nocodazole (Sigma–Aldrich) for 4 h to completely depolymerize microtubules; 100 nM paclitaxel (Cambridge Bioscience) for 6 h to stabilize microtubules; 5 mM tubacin (Sigma–Aldrich) for 4 h to inhibit HDAC6; 300 nM MS-275 (Antibodies Online) for 24 h to inhibit HDAC1/3; 100 nM trichostatin A (Sigma–Aldrich) for 2 h for broad-spectrum HDAC inhibition; or 1 µg ml^−1^ palbociclib for 24 h to arrest cells in G1.

For the viability assays, cells were treated with the indicated concentrations of cisplatin (0.003, 0.010, 0.030, 0.100, 0.300, 1.000, 3.000 or 10.000 µM), doxorubicin (1, 3, 10, 30, 100, 300 or 600 ng ml^−1^), olaparib (0.03, 0.10, 0.30, 1.00, 3.00, 10.00, 30.00 or 60.00 µM; Santa Cruz Biotechnology) or talazoparib (0.3, 1.0, 3.0, 10.0, 30.0, 100.0, 300.0 or 600.0 nM; Selleck Chemicals). Drug washout was performed after 24 h of cisplatin, olaparib or talazoparib treatment, whereas doxorubicin washout was performed after 4 h of treatment. For IR, cells were irradiated with 0.25, 0.5, 2.0 or 5.0 Gy doses.

### siRNA transfection

siRNA transfection was performed in antibiotic-free growth medium using Lipofectamine RNAiMAX (Thermo Fisher Scientific), per the manufacturer’s instructions. Briefly, cells were grown in a six-well plate until they reached ~60% confluency. Before transfection, the growth medium was replaced with 2 ml fresh growth medium without antibiotics. For each well, the transfection solution was prepared as follows: 10 µl Lipofectamine RNAiMAX Transfection Reagent was diluted in 250 µl Reduced Serum Medium Opti-MEM (Thermo Fisher Scientific) in a sterile 1.5-ml microcentrifuge tube, and 5 µl 20 µM stock siRNA (to a final concentration of 40 nM) was diluted in 250 µl Opti-MEM in a separate sterile 1.5-ml microcentrifuge tube. The tubes were then incubated at room temperature for 5 min for equilibration. Opti-MEM solution containing siRNA was then added dropwise onto the tube containing the Lipofectamine RNAiMAX solution and incubated for 20 min at room temperature to allow liposome formation. The solution was then added dropwise onto the six-well and incubated for 6 h. After 6 h the media was removed and fresh growth media was added. Cells were analysed 72 h after transfection. The siRNAs used in this study are listed in Supplementary Table 2.

### Indirect immunofluorescence

Between 1 × 10^4^ and 2 × 10^4^ cells were seeded in a final volume of 50–80 µl on an 18-mm-diameter glass #1.5 coverslips in a 12-well plate. The plate was then incubated at 37 °C for 30 min to allow cell attachment. Once the cells were attached, 1 ml of the growth medium was added and the plate was incubated at 37 °C overnight. On the following day, the growth medium was aspirated and the coverslips were washed with phosphate-buffered saline (PBS) once and fixed immediately with either 4% paraformaldehyde + PBS at room temperature for 15 min or 99.9% ice-cold methanol at −20 °C for 10 min. After fixation, all of the steps were carried out at room temperature. Cells were incubated with permeabilization buffer (PBS + 0.2% Triton X-100) for 5 min. After permeabilization, cells were blocked with 1 ml blocking buffer (PBS, 5% BSA and 0.1% Triton X-100) for 30 min. Then, 30 µl of the diluted primary antibodies were added onto the coverslip and incubated for 30 min, after which another 30 µl was added for another 30 min to avoid the coverslips drying. Next, the coverslips were washed twice with PBS and secondary antibodies (Alexa Fluor conjugated) and incubation was performed in the same way as for the primary antibodies in the dark. Hoechst 33342 solution (Thermo Fisher Scientific) was used at a dilution of 1:10,000 to stain DNA in the dark. Coverslips were washed once with PBS and mounted on microscope slides with a drop of ProLong Diamond Antifade reagent (Thermo Fisher Scientific) on a microscope slide. The primary and secondary antibodies used are listed in Supplementary Table 3.

### Quantification of indirect immunofluorescence images

For the quantification of acetylated tubulin, image files containing *z*-stack images were opened directly in Fiji software using the Bio-Formats Importer. *Z*-stacks were converted to 2D using Sum Slices *z*-projection. To quantify the total fluorescence intensity of single cells, the boundaries of single cells within an image were outlined using the freehand selection tool in the Fiji software and measured for raw integrated density (RID), mean intensity (Mean) and area (AreaCell). After that, a region without fluorescence outside the cell (background) was outlined and measured to obtain the mean background fluorescence (MBg). The background-corrected total fluorescence intensity of a single cell was determined using the formula:

$$\mathrm{Intensity}=\mathrm{RID}-\left(\mathrm{AreaCell}\times \mathrm{MBg}\right)$$

At least 50 cells were analysed per condition for each experiment.

For the quantification of vimentin displacement, cells were stained for vimentin and microtubules and the vimentin fluorescence intensity was determined as previously described^25^. Briefly, the vimentin fluorescence intensity was calculated as the ratio between the fluorescence intensity at the leading edge (15 µm from the leading edge) and that in a 20-µm-radius perinuclear region (ratio < 1 = vimentin associated with the nucleus; ratio > 1 = vimentin displaced towards the cell’s leading edge). At least 50 cells were analysed per condition for each experiment.

For the quantification of 53BP1, γH2AX, RAD51 and phospho-DNA-PK(S2056) foci, cells were stained for DNA (Hoechst), RAD51 and γH2AX or DNA (Hoechst) and 53BP1 and numbers of foci were quantified by automation using CellProfiler. Briefly, maximum-intensity projection was applied to obtain a 2D image from *z* stacks and split into single channels. Individual channel tif files of each image were input into CellProfiler. Nuclei were identified within the image using the IdentifyPrimaryObjects command. 53BP1, RAD51, γH2AX and phospho-DNA-PK(S2056) foci were identified in each nucleus using the IdentifyPrimaryObjects command. Numbers of respective foci in each nucleus were measured using CellProfiler and exported to an Excel file. At least 55 cells were analysed per condition for each experiment.

For the quantification of nuclear p21, image files containing *z*-stack images were directly opened in the Fiji software using Bio-Formats Importer. *Z* stacks were converted to 2D using Sum Slices *z* projection. To quantify the total fluorescence intensity of a nucleus, the threshold tool was used to create a binary mask for each nucleus in the DAPI channel. Using Analyze Particles, a region of interest was generated for each nucleus and subsequently measured for RID, mean intensity and area (AreaNucleus) using the channel containing p21. After that, a region without fluorescence outside the cell (background) was outlined and measured to obtain MBg. The background-corrected total fluorescence intensity of a nucleus was determined using the formula:

$$\mathrm{{Intensity}}=\mathrm{{RID}}-\left(\mathrm{{AreaNucleus}}\times {\mathrm{MBg}}\right)$$

At least 50 cells were analysed per condition for each experiment.

To determine the percentage of cells with large foci, cells were stained for γH2AX and DNA (Hoechst) and manually counted using ImageJ software. Large foci were easily identified as their sizes were >1.75 μm^2^, whereas small foci were ~0.2–0.8 μm^2^. At least 50 cells were analysed per condition for each experiment.

For the quantification of cyclin A-positive cells, cells were stained for cyclin A, 53BP1 and DNA (Hoechst) and manually counted on an Axioplan inverted fluorescence microscope using a Plan-Neofluar oil objective with 63× magnification and a numerical aperture of 1.3. At least 100 cells were counted per condition for each experiment.

All images were acquired using an Eclipse Ti-E inverted microscope (Nikon) equipped with a CSU-X1 spinning disk (Yokogawa), an Andor Zyla 4.2-Plus camera (6.5-μm pixel size), a precision motorized stage, a Piezo Z-drive (Mad City Labs) and the Nikon Perfect Focus system, all controlled by NIS-Elements Advanced Research V5.2 software (Nikon) using a CFI Super Fluor oil objective with 40× magnification and a numerical aperture of 1.3 or a CFI Plan Apochromat Lambda D oil objective with 60× magnification and a numerical aperture of 1.42. Illumination of the samples was done using a Nikon LU-NV laser bed equipped with diode lasers at 405, 488, 561 and 647 nm. The step size was calculated with the following formula ${z}_{\mathrm{step}}=\frac{1.4\lambda \eta }{{(\mathrm{NA})}^{2}}$, where *λ* is the excitation wavelength, *η* is the refractive index of the immersion medium and NA is the numerical aperture of the objective used. Files were saved in the .nd2 file format. The images in Figs. 1b,n, 3a, 4b,g, 5c, 6a,e,g and 7a were acquired using an Evident FV4000 confocal microscope equipped with six SilVIR detectors, solid-state diode lasers (Coherent OBIS) at 405, 488, 561 and 647 nm and a UPlan Apochromat oil high resolution objective with 60× magnification and a numerical aperture of 1.5. Emission detection windows were optimized in the cellSens FluoView software, which was also used to control the system. Files were saved in the .oir file format.

3D reconstruction of cells (Figs. 1n, 3a and 6e,g) was performed with Imaris 9.9 using the Surfaces tool to generate 3D objects of the indicated markers.

### Quantification of RAD51 structure and object area

For the quantification of RAD51 structure, RPE-1 gCtrl and RPE-1 *ATAT1* KO cells were transfected with siCtrl or siRAD54L, as described above. Cells were seeded on coverslips 24 h after siRNA transfection and irradiated at 4 Gy with the X-ray biological irradiator, then fixed at the indicated timepoint (6 or 16 h)^66^. Cells were stained with DNA dye (Hoechst), RAD51 and γH2AX, and images were acquired using an Evident FV4000 confocal microscope (as above) using solid-state diode lasers at 405, 488 and 561 nm and a UPlan Apochromat oil high resolution objective with 60× magnification and a numerical aperture of 1.5. Emission detection windows were optimized in the cellSens FluoView software, which was also used to control the system. *Z*-stack images were captured with a step size of 0.3 μm. Files were saved in the .oir file format. Files containing *z*-stack images were directly opened in the Fiji software using Bio-Formats Importer. RAD51 filament structure was determined as previously described^66^ and quantified manually. At least 20 cells were analysed per condition for each experiment.

### Western blotting

Cells were lysed in 100 µl RIPA buffer (Thermo Fisher Scientific) with added protease inhibitors (Roche; one tablet per 10 ml RIPA) and centrifuged for 20 min at 16,000*g*. For splenocytes, 8- to 9-week-old mice were used (three wild-type males and three *ATAT1* KO males) without enrichment for B cells. Splenocytes were treated with 5 μM tubacin or dimethyl sulfoxide at 4 million per ml in B cell medium. After 2 h of treatment, the splenocytes were washed with ice-cold PBS twice before lysis in RIPA buffer as above. Protein concentration was quantified using Pierce BCA Protein Assay Kits (Thermo Fisher Scientific). Protein samples were normalized by protein concentration. Protein samples were prepared per well by mixing with NuPAGE LDS Sample Buffer (4X) (Invitrogen) and NuPAGE Sample Reducing Agent (10X) (Invitrogen) and boiled for 5 min at 95 °C. Then, 10–20 µg of the protein samples were loaded into NuPAGE Bis-Tris Mini Protein Gels (Invitrogen) per well. Proteins were transferred onto polyvinylidene difluoride membranes. Membranes were blocked in 5% skimmed milk in 1× Tris-buffered saline containing 0.1% Tween-20 (TBS-T) for 1 h at room temperature, then incubated with the respective primary antibodies diluted in 5% skimmed milk in 1× TBS-T overnight at 4 °C. Membranes were washed with 1× TBS-T three times, then incubated with the respective secondary antibodies diluted in 5% skimmed milk in 1× TBS-T. Membranes were washed with 1× TBS-T three times. Membranes were developed with SuperSignal West Pico PLUS Chemiluminescent Substrate (Thermo Fisher Scientific) and imaged with an Amersham Imager 600 (GE Healthcare). The western blot in Extended Data Fig. 4b was analysed using FIJI. Band intensities for the proteins of interest (histone H3, acetylated histone H3, α-tubulin and acetylated tubulin) were measured in the same samples. The intensity of each acetylated protein was measured as described and normalized to the corresponding total protein intensity within the same sample. The antibodies used for western blot analysis are listed in Supplementary Table 4.

### Cell-cycle analysis

A total of 1 × 10^6^ RPE-1, RPE-1 gCtrl and RPE-1 *ATAT1* KO cells were irradiated at 2 Gy with the X-ray biological irradiator. Cells were harvested at the indicated time points and washed with cold PBS. Cells were then fixed immediately in cold 70% ethanol overnight at 4 °C. Cells were washed twice with PBS. Cells were incubated with 50 µg ml^−1^ RNase A (Thermo Fisher Scientific) in PBS at 37 °C for 30 min, followed by incubation in 20 µg ml^−1^ propidium iodide solution (Sigma–Aldrich) on ice in the dark for at least 30 min. Cell-cycle distribution data were acquired using a Thermo Fisher Attune NxT and analysed using FlowJo 10. The percentages of cells in the G1, S and G2/M phases were calculated. At least 10,000 cells were analysed per condition in each experiment.

### Live-cell imaging

All live imaging experiments were performed with an Eclipse Ti-E inverted microscope (Nikon) equipped with a CSU-X1 spinning disk (Yokogawa), an Andor Zyla 4.2-Plus camera (6.5-μm pixel size), a precision motorized stage, a Piezo z-drive (Mad City Labs) and a Nikon Perfect Focus system, all controlled by NIS-Elements Advanced Research V5.2 Software (Nikon) using a CFI Super Fluor oil objective with 40× magnification and a numerical aperture of 1.3 or a CFI Plan Apochromat Lambda D oil objective with 60× magnification and a numerical aperture of 1.42. The microscope was enclosed in a temperature- and CO_2_-controlled environment (Okolab) that maintained an atmosphere of 37 °C under 5% humidified CO_2_ for live-cell imaging. Illumination of the samples was done using the Nikon LU-NV laser bed equipped with diode lasers at 405, 488, 561 and 647 nm. *Z*-stack images were captured with the step size calculated as described above. All files were saved in the .nd2 file format.

To visualize nuclear deformations and DSB mobility, RPE-1 53BP1–RFP H2B–GFP cells were seeded overnight in eight-well glass-bottom chambers (ibidi) at a seeding density of 7.5 × 10^4^ cells per well (for IR treatment) or 4 × 10^4^ cells per well (for cisplatin treatment). Cisplatin treatment (2 µM; 24 h) and irradiation (2 Gy) were performed the following day, and cells were imaged 24 h later (cisplatin) or at the indicated time points following irradiation. Where indicated, we added: palbociclib (1 µM) 24 h before irradiation, paclitaxel (100 nM) 6 h before imaging or nocodazole 4 h before imaging. Images were taken every 30 s over a period of 10 min (*t* = 20 frames) at 2 × 2 binning on the camera.

To examine the localization of centrosomes during the DDR, RPE-1 centrin1–GFP cells were seeded overnight in eight-well glass-bottom chambers (ibidi) at a seeding density of 4 × 10^4^ cells per well. Cisplatin treatment (2 µM; 24 h) was performed the following day, and cells were imaged 24 h later. At 30 min before imaging, cells were washed once with PBS, and media containing Hoechst 33342 (1:15,000) was added for 10 min at 37 °C. Cells were washed once with PBS, and fresh medium (pre-warmed at 37 °C) was added before the cells were imaged. Images were taken every 30 s over a period of 10 min (*t* = 20 frames) at 2 × 2 binning on the camera.

To visualize the clustering of DNA damage foci in *ATAT1* KO cells, *ATAT1* KO 53BP1–mCherry cells were treated with cisplatin (2 µM). At 24 h after cisplatin treatment, the drug was washed out (as described above). At 24 h after cisplatin washout, cells were seeded in eight-well glass-bottom chambers (ibidi) and left to adhere overnight. The next day (2 days after cisplatin washout), cells were imaged over a period of 24 h with a time interval of 10 min (*t* = 144 frames) at 2 × 2 binning on the camera.

### Quantification of nuclear shape fluctuations

For the quantification of the nuclear fluctuations in live cells, files containing *z*-stack images were opened in the Fiji software using Bio-Formats Importer. Single cells were cropped and registration of the images over time was performed using the HyperStackReg plugin with the Rigid Body option^98^. Analysis was performed using a custom script developed by the group, available at https://doi.org/10.5281/zenodo.22111658 (ref. ^99^). In brief, maximum-intensity projections of *z* stacks were made for the nuclear marker channel (for example, H2B–GFP) and a smaller Gaussian blur was applied to reduce noise. Nuclei were segmented using Li thresholding, and regions of interest were calculated for each frame using the Analyze Particles function, as well as the centroid of the nucleus. Outlines of the nucleus at each timepoint were then created in a separate image window before subsequent polar transformation from Cartesian coordinates to polar coordinates (using the Polar Transformer plugin). The resulting image was then binarized and skeletonized before exporting using the Save XY Coordinates option. Images of the nucleus outline and the polar-transformed nuclear outlines were also saved. To analyse the data, a custom Excel VBA macro was written (available at https://doi.org/10.5281/zenodo.22111658). In brief, the raw data were cleaned up so that every *x* coordinate only had one *y* coordinate value. The *y* coordinate value for each *x* coordinate at frame 0 (the first frame) was subtracted from every subsequent frame. This resulted in calculating the change in pixel position for each frame, which corresponded to changes in the nuclear shape and could be plotted. To calculate cumulative fluctuations, the raw fluctuation values were converted into absolute values (as fluctuations can have both positive and negative integers) and summed for each frame. These summed frames were then added cumulatively to see the total cumulative fluctuation over time. At least 20 cells were analysed per condition.

For the quantification of centrosome positioning and nuclear fluctuation correlation in live cells, polar transformation was performed as above for the nucleus channel. A second round of polar transformation was performed on the centrosome channel using the centroid *x* and *y* position of the nucleus. The average *x* position of the centrosome was then calculated for each cell, and the *x* position of the nucleus data was normalized so that the centrosome position was 0. The average range of centrosome movement across all time frames was calculated to be approximately 40° (−20 to +20° relative to the average centrosome *x* position at 0). For the histogram, each cell was normalized to its total cumulative fluctuation to account for greater variance in values when the data were binned. At least 20 cells were analysed per condition.

### Quantification of DSB mobility

Quantification of DSB foci tracking in live cells was performed as previously described^13^. Briefly, the tracking of 53BP1–RFP foci was performed with FIJI software on at least 20 cells per condition. Cells were registered by the HyperStackReg plugin using Rigid Body (Sharma et al. ^98^) and particles were tracked using the MosaicSuite Particle Tracker plugin^100^ with the following parameters for particle detection and tracking: radius=3 pixels; cutoff=0.001 pixels; link range=1; displacement=10 pixels. The *x*, *y* and *z* coordinates of each trajectory were used to calculate the total distance foci moved between each timeframe. All 53BP1–RFP foci in a cell that were continuously tracked for at least seven out of 21 frames were analysed. To calculate MSD, only *x* and *y* movements were considered. For each track, first the squared displacement was calculated between each time point. Then for each treatment, the MSD of all tracks was calculated. The diffusion coefficient was calculated as the exponent of fitting a simple power law to the ensemble MSD–time data. This analysis was done in a custom Python script.

### 3D analysis of the localization of DNA damage foci

To visualize the localization of DNA damage foci within the nucleus, cells expressing 53BP1–mCherry were seeded on eight-well glass-bottom chambers (ibidi). For experiments where latrunculin A was used, treatments were carried out simultaneously with cisplatin for 24 h to visualize small foci or for 2 days after cisplatin washout to visualize large foci. For all of the experiments, cells were incubated with the DNA dye Hoechst (1:5,000) for 30 min before imaging. Following Hoechst washout, live cells were imaged using an Evident FV4000 microscope equipped with a UPlan Apochromat oil high resolution objective with 60× magnification and a numerical aperture of 1.5. The microscope was enclosed within temperature- and CO_2_-controlled environments that maintained an atmosphere of 37 °C under 5% humidified CO_2_ for live-cell imaging (Pecon). Orthogonal maximum-intensity projections of each cell were generated using Fiji software (20 cells per condition for each experiment). To analyse the localization of small foci within the nucleus, *x–y* coordinates of the nuclear outline and *x–y* coordinates of the centroids of each individual foci were exported from Fiji. The shortest distance of each centroid to the nuclear outline (that is, the nuclear border) was calculated using Excel. For large foci, a similar analysis was performed measuring the shortest distance between the border and the nuclear outline using Fiji software.

### Quantification of senescence

To assess senescence, cells were seeded on glass coverslips and fixed with 4% paraformaldehyde for 3 min at room temperature at the indicated time points following cisplatin washout. Cells were then washed three times with Hanks’ Balanced Salt Solution (Thermo Fisher Scientific) and incubated with SPiDER-Bgal reagent (Dojindo) and the DNA dye Hoechst for 30 min at 37 °C. Cells were washed twice with Hanks’ Balanced Salt Solution and coverslips were then mounted on a drop of ProLong Diamond Antifade reagent (Thermo Fisher Scientific) on a microscope slide. The number of β-galactosidase^+^ cells was manually counted using an Axioplan inverted fluorescence microscope with a Plan Apochromat oil objective with 63× magnification and a numerical aperture of 1.4. At least 100 cells were counted per condition for each experiment.

### Quantification of nuclear stiffness by AFM

Cells transfected with siRNA were plated on 35-mm glass-bottom dishes (World Precision Instruments) at 5 × 10^4^ cells per well and treated with cisplatin (2 µM; 24 h). Before imaging, the cell medium was changed to L15 medium supplemented with 10% FBS for AFM measurements. Measurements were performed on a NanoWizard 4 atomic force microscope (Bruker) interfaced to an inverted optical microscope. Experiments were performed using MLCT-D cantilevers (nominal stiffness: 0.03 N m^−1^; calibrated: 0.015–0.045 N m^−1^). Cells were indented above the nucleus with a setpoint force of 2 nN and an approach–retract ramp speed of 4 μm s^−1^. Force–distance curves were processed for baseline correction, contact point definition and cantilever deflection correction using published algorithms implemented in custom MATLAB scripts. The apparent Young’s modulus was calculated accounting for the pyramidal tip shape, fitting the experimental curves up to an indentation depth of 1 μm. This depth was chosen to ensure deformation of the nuclear lamina. Samples were measured at least three times, with at least 20 cells per sample.

### Neutral comet assay

Cells were seeded at a density of 1 × 10^5^ cells per well in 2 ml medium in a six-well plate. Cells were then treated with 2 μM cisplatin for 24 h and washed out (as described above). Cells were harvested at the respective timepoint. The comet assay was performed according to the manufacturer’s instructions (4250-050-K; Bio-Techne). Briefly, lysis solution was cooled at 4 °C for at least 20 min. LMAgarose was melted in boiling water and then cooled down in a 37 °C water bath for at least 20 min. Cells were diluted at 1 × 10^5^ cells per ml in Ca^++^- and Mg^++^-free 1× PBS. The molten LMAgarose was combined with cells at 37 °C at a ratio of 1:10 and immediately 60 μl of this solution was pipetted onto the comet slide. Slides were placed flat at 4 °C in the dark for 10 min and then immersed and incubated in lysis solution at 4 °C for 1 h. The slides were then incubated in 50 ml 1× TBE buffer (Severn Biotech) for 15 min at 4 °C in the dark. Neutral electrophoresis was performed in 1× TBE buffer at 30 V for 30 min. Slides were washed in distilled water for 5 min, then incubated in 70% ethanol for 5 min. The samples were then dried at 37 °C for 15 min. SYBR Gold Nucleic Acid Gel Stain (Invitrogen) was diluted in TE buffer (1:30,000). Each sample was stained with 100 μl diluted SYBR Gold for 30 min at room temperature in the dark. Samples were rinsed briefly in distilled water and dried at 37 °C for 10 min. Slides were imaged with an LSM 710 point-scanning confocal microscope (Zeiss) with a Plan Apochromat objective with 10× magnification and a numerical aperture of 0.45. Illumination was achieved at 496 nm with a multiline argon laser, with the detection window of a GaAsP detector set according to ZEN 2009 (black edition) software and saved as .lsm files. The tail moment (calculated as the tail length multiplied by the percentage of DNA in the tail) was measured using CometAnalyser^101^. Briefly, image files were converted to eight-bit .tif files in the Fiji software, which were then opened as fluorescence images in CometAnalyser. Comets in the images were selected manually using the select a comet command. Tail moment measurements were exported to an Excel file using the export measurements command. At least 50 comets were analysed per condition in each experiment.

### AlphaFold modelling of protein interaction

FASTA sequences of αTAT1 and αTAT1^1–190^ (ATAT1_HUMAN), α-tubulin (TUBA1B_HUMAN), β-tubulin (TUBB_HUMAN) and the histones H1X (H1X_HUMAN), H2A (H2A2A_HUMAN), H3.1 (B4E380_HUMAN) and H4 (H4_HUMAN) were obtained from UniProt. These FASTA sequences were then input into the AlphaFold 3 (AF3) server (https://alphafoldserver.com/; Google DeepMind)^46^ to obtain predicted structures of αTAT1^1–190^, α-tubulin, β-tubulin and the histones H1X, H2A, H3 and H4. Two entities were uploaded to AF3 to predict structural interactions between αTAT1^1–190^ and the above proteins. The predicted interaction structure between the two entities and predicted alignment error information was available to download for further analysis. AF3 computes an overall pTM score for global structural confidence and an ipTM score for inter-chain interaction confidence. pTM and ipTM values of 0.8–1.0 are considered high in confidence, whereas 0.6–0.8 is considered moderate in confidence and <0.5 is considered low in confidence. The highest-ranked structural model (model_0) was used for analysis as this has the highest predicted local distance difference test score. A single interaction rank score was calculated as (0.8 × ipTM) + (0.2 × pTM). The .cif file for each prediction was uploaded to PyMOL (The PyMOL Molecular Graphics System, Version 3.1; Schrödinger) for structural analysis of the interaction folds. Structural comparisons and alignments to confirm the structural similarity between αTAT1 and αTAT1^1–190^ were carried out using TM-align^102^.

### In vitro CSR assay

Splenocyte suspensions were prepared by smashing spleens from 21-week-old naive mice of respective genotypes (one wild-type male and one *ATAT1* KO male) through 70-µm filters (Corning) in FACS buffer (PBS + 2% FBS + 2 mM EDTA), followed by red blood cell lysis with ACK buffer (Gibco). Cells were then pelleted, resuspended in 1 ml FACS buffer and filtered again with a 70-µm filter before cell counting. Splenic B cells were enriched by CD43 (Ly-48) negative selection microbeads (Miltenyi Biotec) using MACS LS columns (Miltenyi Biotec). Enriched B cells were labelled using a CellTrace Violet Cell Proliferation Kit (Invitrogen), per the manufacturer’s protocol. For CD40 stimulation, labelled B cells were stimulated with 1 µg ml^−1^ recombinant murine CD40 ligand (Bio-Techne) and 10 ng ml^−1^ murine IL-4 (PeproTech) at 0.5 million per ml in B cell medium (DMEM, High Glucose, GlutaMAX Supplement medium (Gibco) supplemented with 10% FBS (Gibco), 100 U ml^−1^ penicillin–streptomycin solution (Gibco), 1 mM sodium pyruvate (Gibco), 1% MEM Non-Essential Amino Acids Solution (Gibco), 10 mM HEPES (Gibco) and 50 µM β-mercaptoethanol). For LPS stimulation, labelled B cells were stimulated with 10 µg ml^−1^ LPS (Enzo Life Sciences) at 0.5 million per ml in B cell medium. After 3 or 4 days of stimulation, cells were harvested, washed with pre-chilled FACS buffer and stained immediately for the extracellular antibody panel (table below) on ice. A Zombie NIR kit (BioLegend) was used to analyse dead versus live cells. After extracellular staining, cells were fixed with Fixation Buffer (BioLegend) for 15 min. All samples (three technical replicates per genotype) were acquired on the cytometer on the same day. The antibodies used are listed in Supplementary Table 5.

### HR reporter assay

HR efficiency was measured using the DR–GFP reporter assay, in which the GFP^+^ cells were identified as repaired by HR^61^. DR–GFP HEK-293M cells were generated by transfecting 2 µg pDR–GFP using Lipofectamine 2000 (Thermo Fisher Scientific) as described in the section ‘Lentiviral generation and transduction’ above. Cells were treated with 2 µg ml^−1^ puromycin (Sigma–Aldrich) to undergo selection for 1 week. Some 1 × 10^6^ DR–GFP HEK-293M cells were seeded into a six-well plate. Cells were then transfected with siCtrl or siαTAT1 as described above. At 24 h after siRNA transfection, cells were transfected with 1 µg SceI (pCBASceI) and 1 µg mCherry (mCherry2–C1) using Lipofectamine 2000. Cells were then harvested 72 h after SceI and mCherry transfection. GFP^+^ cells were quantified by FACS using a BD Biosciences LSR Fortessa and analysed using FlowJo 10. For HR reporter analysis, the mCherry^+^ population was used to identify successfully transfected cells. Within the mCherry^+^ population, the percentage of GFP^+^ cells was quantified. The background GFP signal was determined from the percentage of GFP^+^ cells within the mCherry^+^ population under the SceI^−^ condition. For each experimental condition, the percentage of GFP^+^ cells was calculated and corrected by subtracting the percentage of GFP^+^ cells measured in the Scel^−^ condition. The pDR–GFP and pCBASceI plasmids was obtained via Addgene (plasmids #26475 (RRID: Addgene_26475) and #26477 (RRID: Addgene_26477))^103^. The mCherry2–C1 plasmid was also obtained from Addgene (plasmid #54563 (RRID: Addgene_54563)). At least 50,000 cells were analysed per condition in each experiment.

### CyQUANT cell viability assay

For the cell growth rate experiments, RPE-1 gCtrl and RPE-1 *ATAT1* KO pooled or individual clones (#2, #20, #34 and #40) were seeded at a density of 250, 500 or 750 cells per well in a final volume of 100 µl in 96-well optical-bottom, black-walled microplates and incubated at 37 °C for 4 days before the quantification of cellular DNA content. For the viability experiments, RPE-1 gCtrl and RPE-1 *ATAT1* KO cells (clones #2 and #20) were seeded at a density of 100 cells per well in a final volume of 100 µl in 96-well optical-bottom, black-walled microplates and incubated at 37 °C to allow cell attachment before treatment. Cells were treated with cisplatin (0.003–10.000 µM), olaparib (0.03–60.00 µM) or talazoparib (0.3–600.0 nM) for 24 h or doxorubicin (1–600 ng ml^−1^) for 4 h. For the irradiation experiments, cells were irradiated at 0.25, 0.5, 2.0 or 5.0 Gy. Cells were then maintained in growth medium for 7 days before quantification of the cellular DNA content.

The cellular DNA content was quantified using the CyQUANT Direct Cell Proliferation Assay (Invitrogen) according to the manufacturer’s instructions. A 2× CyQUANT working solution was prepared in PBS by diluting the background suppressor 1:50 and the nucleic acid stain 1:250. For each well, 100 µl of the 2× CyQUANT solution was added directly to the existing 100 µl of culture medium. Plates were incubated at 37 °C for 1 h. Fluorescence was measured using a FLUOstar Omega plate reader with excitation and emission wavelengths of 480 and 520 nm, respectively, using bottom-read optics. The instrument gain was set to 70% and adjusted using two blank wells and two untreated control wells per plate. The background fluorescence, determined from wells containing medium and CyQUANT solution without cells, was subtracted from all measurements. Fluorescence values from treated conditions were normalized to the corresponding untreated controls.

### Statistical analysis and study design

Sample sizes (per condition and per experiment) are specified in each figure caption and Methods. Sample sizes were determined empirically based on our own experience and, where relevant, previous publications. Randomization of samples and blind data collection were not performed in this study. No data were excluded from the analysis unless the experiment did not work, as assessed by the use of positive and negative controls.

Graphs and statistics were generated using Prism 10 (GraphPad Software), where the results are presented as means ± standard deviation unless otherwise stated. Statistical analyses for data that followed a normal distribution were performed using one-way analysis of variance (three or more independent groups), unpaired *t*-test (two independent groups) or one-sample *t*-test for normalized data (one independent group using a hypothetical mean of 1). For data that were not normally distributed, nonparametric statistical analyses were performed using the Kruskal–Wallis test (three or more independent groups) or Mann–Whitney *U* test (two independent groups). Data with two independent variables were analysed by two-way analysis of variance. The statistical analyses performed for each experiment are provided in Supplementary Table 6.

### Reporting summary

Further information on research design is available in the Nature Portfolio Reporting Summary linked to this article.

## Online content

Any methods, additional references, Nature Portfolio reporting summaries, source data, extended data, supplementary information, acknowledgements, peer review information; details of author contributions and competing interests; and statements of data and code availability are available at https://doi.org/10.1038/s41556-026-02096-3.

## Supplementary information

Reporting Summary

Peer Review File

Supplementary Table 1 Primer sequences for cell line generation.

Supplementary Table 2 Details of the siRNAs used.

Supplementary Table 3 Antibodies and dilutions used for immunofluorescence.

Supplementary Table 4 Antibodies and dilutions used for western blotting.

Supplementary Table 5 Antibodies and dilutions for CSR.

Supplementary Table 6 Statistical tests used.

Supplementary Table 7 Light microscopy reporting table.

Supplementary Video 1 Live-cell imaging of cells expressing 53BP1–RFP, to track DSBs after 24 h of cisplatin treatment before imaging. Images were acquired every 30 s for 10 min. Single video frames have been extracted and are presented in Fig. 1h.

Supplementary Video 2 Live-cell imaging of cells expressing 53BP1–RFP, to track DSBs after 24 h of cisplatin treatment before imaging. Images were acquired every 30 s for 10 min for: (i) siCtrl, (ii) siαTAT1, (iii) siKIF5B and (iv) nocodazole treatments. Single video frames have been extracted and are presented in Fig. 1k.

Supplementary Video 3 Live-cell imaging of cells expressing 53BP1–RFP, to track DSBs after 2-Gy irradiation before imaging. Images were acquired every 30 s for 10 min: (i) 2 h after IR, (ii) 8 h after IR and (iii) 2 h after IR and nocodazole treatment. Single video frames have been extracted and are presented in Fig. 1m.

Supplementary Video 4 Live-cell imaging of cells expressing 53BP1–RFP, to track DSBs after 24 h of cisplatin treatment before imaging. Images were acquired every 30 s for 10 min for: (i) Ctrl and (ii) αTAT1-overexpressing cells. Single video frames have been extracted and are presented in Extended Data Fig. 2g.

Supplementary Video 5 3D surface reconstruction of a live-cell nucleus (siCtrl) stained for DNA (Hoechst) and expressing 53BP1–RFP. The cell was treated with cisplatin for 24 h before imaging. A single video frame has been extracted and is presented in Fig. 1n.

Supplementary Video 6 3D surface reconstruction of a live-cell nucleus (siαTAT1) stained for DNA (Hoechst) and expressing 53BP1–RFP. The cell was treated with cisplatin for 24 h before imaging. A single video frame has been extracted and is presented in Fig. 1n.

Supplementary Video 7 Live-cell imaging of cells expressing H2B–GFP, to assess nuclear fluctuations after 24 h of cisplatin treatment. Images were acquired every 30 s for 10 min. Single video frames have been extracted and are presented in Fig. 2a.

Supplementary Video 8 Live-cell imaging of cells expressing centrin1–GFP and stained for DNA (Hoechst), to assess nuclear fluctuations relative to centrosome position after 24 h of cisplatin treatment. Images were acquired every 30 s for 10 min. Single video frames have been extracted and are presented in Fig. 2e.

Supplementary Video 9 Live-cell imaging of cells expressing 53BP1–RFP, to track DSBs after cisplatin treatment for 24 h before imaging. Images were acquired every 30 s for 10 min for: (i) dimethyl sulfoxide (DMSO) and (ii) paclitaxel treatment. Single video frames have been extracted and are presented in Extended Data Fig. 3d.

Supplementary Video 10 Live-cell imaging of cells expressing 53BP1–RFP, to track DSBs after cisplatin treatment for 24 h before imaging. Images were acquired every 30 s for 10 min for: (i) siCtrl, (ii) siαTAT1, (iii) siVim, (iv) siVim + siαTAT1 and (v) siVim and nocodazole treatment. Single video frames have been extracted and are presented in Fig. 3g.

Supplementary Video 11 Live-cell imaging of cells expressing 53BP1–RFP, to track DSBs after cisplatin treatment for 24 h before imaging. Images were acquired every 30 s for 10 min for: (i) siCtrl, (ii) siαTAT1, (iii) siPlec, (iv) siPlec + siαTAT1 and (v) siPlec and nocodazole treatment. Single video frames have been extracted and are presented in Extended Data Fig. 6k.

Supplementary Video 12 Live-cell imaging of *ATAT1* KO cells expressing 53BP1–mCherry, to track DSBs 2 days after IR. Images were acquired every 30 s for 10 min for: (i) cells arrested in G1 with palbociclib 2 h after IR, (ii) 8 h after IRand (iii) cells arrested with palbociclib 8 h after IR. Single video frames have been extracted and are presented in Extended Data Fig. 8h.

Supplementary Video 13 Live-cell imaging of *ATAT1* KO cells expressing 53BP1–mCherry, to track DSBs 2 days after cisplatin washout. Images were acquired every 10 min for 7.6 h. Single video frames have been extracted and are presented in Fig. 6d.

Supplementary Video 14 3D surface reconstruction of a live-cell nucleus from an *ATAT1* KO cell stained for DNA (Hoechst) and expressing 53BP1–RFP 24 h after cisplatin treatment. A single video frame has been extracted and is presented in Fig. 6e.

Supplementary Video 15 3D surface reconstruction of a live-cell nucleus from an *ATAT1* KO cell stained for DNA (Hoechst) and expressing 53BP1–RFP 1 day after cisplatin washout. A single video frame has been extracted and is presented in Fig. 6e.

Supplementary Video 16 3D surface reconstruction of a live-cell nucleus from an *ATAT1* KO cell stained for DNA (Hoechst) and expressing 53BP1–RFP 2 days after cisplatin washout. A single video frame has been extracted and is presented in Fig. 6e.

Supplementary Video 17 3D surface reconstruction of a live-cell nucleus from an *ATAT1* KO cell stained for DNA (Hoechst) and expressing 53BP1–RFP 3 days after cisplatin washout. A single video frame has been extracted and is presented in Fig. 6e.

Supplementary Video 18 3D surface reconstruction of a live-cell nucleus from an *ATAT1* KO cell stained for DNA (Hoechst) and expressing 53BP1–RFP 24 h after cisplatin treatment. A single video frame has been extracted and is presented in Fig. 6g.

Supplementary Video 19 3D surface reconstruction of a live-cell nucleus from an *ATAT1* KO cell stained for DNA (Hoechst) and expressing 53BP1–RFP treated with latrunculin A after cisplatin treatment for 24 h. A single video frame has been extracted and is presented in Fig. 6g.

Supplementary Video 20 3D surface reconstruction of a live-cell nucleus from an *ATAT1* KO cell stained for DNA (Hoechst) and expressing 53BP1–RFP 3 days after cisplatin washout. A single video frame has been extracted and is presented in Fig. 6g.

Supplementary Video 21 3D surface reconstruction of a live-cell nucleus from an *ATAT1* KO cell stained for DNA (Hoechst) and expressing 53BP1–RFP treated with latrunculin A and 3 days after cisplatin washout. A single video frame has been extracted and is presented in Fig. 6g.

## Source data

Source Data Fig. 1 Statistical source data.

Source Data Fig. 2 Statistical source data.

Source Data Fig. 3 Statistical source data.

Source Data Fig. 4 Statistical source data.

Source Data Fig. 5 Statistical source data.

Source Data Fig. 6 Statistical source data.

Source Data Fig. 7 Statistical source data.

Source Data Extended Data Fig. 1 Statistical source data.

Source Data Extended Data Fig. 2 Statistical source data.

Source Data Extended Data Fig. 2 Unprocessed western blots.

Source Data Extended Data Fig. 3 Statistical source data.

Source Data Extended Data Fig. 4 Statistical source data.

Source Data Extended Data Fig. 4 Unprocessed western blots.

Source Data Extended Data Fig. 5 Statistical source data.

Source Data Extended Data Fig. 6 Statistical source data.

Source Data Extended Data Fig. 6 Unprocessed western blots.

Source Data Extended Data Fig. 7 Statistical source data.

Source Data Extended Data Fig. 8 Statistical source data.

Source Data Extended Data Fig. 9 Statistical source data.

Source Data Extended Data Fig. 9 Unprocessed western blots.

Source Data Extended Data Fig. 10 Statistical source data.

## Data Availability

All of the data that support the findings of this study are readily available upon request. Source data are provided with this paper.

## Code availability

A custom script developed by the laboratory of S.A.G. for analysing nuclear fluctuations is available at https://doi.org/10.5281/zenodo.22111658.
