# Supplementary material for: Tubulin acetylation regulates nuclear shape fluctuations and DNA repair
Source: Nat Cell Biol. 2026 Oct 28;28(11):2305–20. doi: 10.1038/s41556-026-02096-3 (PMC13751303; doi:10.1038/s41556-026-02096-3)
Supplement: Supplementary file 1 [file 41556_2026_2096_MOESM1_ESM.pdf]

nature portfolio

Corresponding author(s): Susana Godinho

Last updated by author(s): Sep 30, 2025

Reporting Summary

Nature Portfolio wishes to improve the reproducibility of the work that we publish. This form provides structure for consistency and transparency in reporting. For further information on Nature Portfolio policies, see our Editorial Policies and the Editorial Policy Checklist.

Statistics

For all statistical analyses, confirm that the following items are present in the figure legend, table legend, main text, or Methods section.

n/a	Confirmed	
☐	☒	The exact sample size (*n*) for each experimental group/condition, given as a discrete number and unit of measurement
☐	☒	A statement on whether measurements were taken from distinct samples or whether the same sample was measured repeatedly
☐	☒	The statistical test(s) used AND whether they are one- or two-sided<br>*Only common tests should be described solely by name; describe more complex techniques in the Methods section.*
☐	☒	A description of all covariates tested
☐	☒	A description of any assumptions or corrections, such as tests of normality and adjustment for multiple comparisons
☐	☒	A full description of the statistical parameters including central tendency (e.g. means) or other basic estimates (e.g. regression coefficient) AND variation (e.g. standard deviation) or associated estimates of uncertainty (e.g. confidence intervals)
☐	☒	For null hypothesis testing, the test statistic (e.g. F, t, r) with confidence intervals, effect sizes, degrees of freedom and P value noted<br>*Give P values as exact values whenever suitable.*
☒	☐	For Bayesian analysis, information on the choice of priors and Markov chain Monte Carlo settings
☒	☐	For hierarchical and complex designs, identification of the appropriate level for tests and full reporting of outcomes
☒	☐	Estimates of effect sizes (e.g. Cohen's *d*, Pearson's *r*), indicating how they were calculated

Our web collection on statistics for biologists contains articles on many of the points above.

Software and code

Policy information about availability of computer code

Data collection	*Provide a description of all commercial, open source and custom code used to collect the data in this study, specifying the version used OR state that no software was used.*
Data analysis	GraphPad Prism 10 was used to generate all graphics and perform statistic analyses.<br>Densitometry analyses of western blots were performed using Fiji<br>All image analysis was performed in FIJI. Double strand break (DSB) mobility used the MOSAIC tools for 3D particle tracking, nuclear fluctuations required the Polar Transformer class file in FIJI<br>Quantification of DSB number was performed in CellProfiler 4.2.4 using a custom pipeline of standard module<br>Custom code used deposited on Zenodo (described in the methods): https://doi.org/10.5281/zenodo.22111658<br>CometAnalyser software was used to analyze comet assay.

For manuscripts utilizing custom algorithms or software that are central to the research but not yet described in published literature, software must be made available to editors and reviewers. We strongly encourage code deposition in a community repository (e.g. GitHub). See the Nature Portfolio guidelines for submitting code & software for further information.

Data

Policy information about availability of data

All manuscripts must include a data availability statement. This statement should provide the following information, where applicable:
- Accession codes, unique identifiers, or web links for publicly available datasets
- A description of any restrictions on data availability
- For clinical datasets or third party data, please ensure that the statement adheres to our policy

All data are readily available at request

Research involving human participants, their data, or biological material

Policy information about studies with human participants or human data. See also policy information about sex, gender (identity/presentation), and sexual orientation and race, ethnicity and racism.

Reporting on sex and gender	N/A
Reporting on race, ethnicity, or other socially relevant groupings	N/A
Population characteristics	N/A
Recruitment	N/A
Ethics oversight	N/A

Note that full information on the approval of the study protocol must also be provided in the manuscript.

Field-specific reporting

Please select the one below that is the best fit for your research. If you are not sure, read the appropriate sections before making your selection.

☒ Life sciences ☐ Behavioural & social sciences ☐ Ecological, evolutionary & environmental sciences

For a reference copy of the document with all sections, see nature.com/documents/nr-reporting-summary-flat.pdf

Life sciences study design

All studies must disclose on these points even when the disclosure is negative.

Sample size	Sample size was determined empirically based on our own experience and previous publications. The sample size (per condition and per experiment) are specified within each figure legend and methods section
Data exclusions	No data were excluded from the analysis unless the experiment did not work. we use positive and negative controls in all our experiments to ensure the experiment has been performed correctly.
Replication	All experiments were replicated at least 3 times to ensure they were reproducible, unless otherwise stated in the figure legend
Randomization	Randomization of samples was not preformed in this study.
Blinding	Investigators were not blinded to data collection. Analysis methods were generally performed in batch to remove unconscious bias

Reporting for specific materials, systems and methods

We require information from authors about some types of materials, experimental systems and methods used in many studies. Here, indicate whether each material, system or method listed is relevant to your study. If you are not sure if a list item applies to your research, read the appropriate section before selecting a response.

Materials & experimental systems

n/a	Involved in the study
☐	☒ Antibodies
☐	☒ Eukaryotic cell lines
☐	☐ Palaeontology and archaeology
☐	☐ Animals and other organisms
☐	☐ Clinical data
☐	☐ Dual use research of concern
☐	☐ Plants

Methods

n/a	Involved in the study
☐	☐ ChIP-seq
☐	☒ Flow cytometry
☐	☐ MRI-based neuroimaging

Antibodies

Antibodies used

All antibodies used are described in the materials and are commercially available and fully listed within the methods section with all information:
IF:
Antibody (Clone) Species Catalogue number Manufacturer Dilution Fixation
Vimentin Rabbit 5741 Cell Signaling 1:200 Paraformaldehyde (PFA)
Acetylated
tubulin (6-11B-1) Mouse T6793 Sigma Aldrich 1:2500 Methanol (MeOH)
53BP1 Rabbit A300-273A Bethyl Laboratories 1:750 MeOH/PFA
RAD51 Rabbit Ab63801 Abcam 1:1000 PFA
H2AX (phospho-Ser139) Mouse 05-636 Merck Millipore 1:800 MeOH/PFA
α-tubulin (DM1α) Mouse T9026 Sigma Aldrich 1:1000 MeOH
α-Tubulin (AA345-Hu) Human AA345 ABCD Antibodies 1:800 MeOH/PFA
Cyclin A (B-8 clone) Mouse SC271682 Santa Cruz 1:400 PFA
p21 Rabbit 2947 Cell Signaling 1:1000 MeOH
phospho-DNA-PK(S2056) Rabbit ab18192 Abcam 1:600 PFA
Anti-Mouse Alexa Fluor 488 Goat A11001 Invitrogen 1:1000
Anti-Mouse Alexa Fluor 568 Goat A11004 Invitrogen 1:1000
Anti-Mouse Alexa Fluor 647 Goat A21235 Invitrogen 1:1000
Anti-Rabbit Alexa Fluor 488 Goat A11008 Invitrogen 1:1000
Anti-Rabbit Alexa Fluor 568 Goat A11011 Invitrogen 1:1000
Anti-Human Alexa Fluor 568 Goat A21090 Invitrogen 1:1000

WB:
Antibody Species Catalogue number Manufacturer Dilution
Kif5b Mouse Ab167429 Abcam 1:1000
Vimentin Rabbit 5741 Cell Signaling 1:1000
Plectin (10F6) Mouse sc-33649 Santa Cruz 1:500
β-actin Rabbit 4970 Cell Signaling 1:5000553437
α-tubulin (DM1α) Mouse T9026 Sigma Aldrich 1:3000
Acetylated tubulin Mouse T6793 Sigma Aldrich 1:3000
Histone H3 Acetylation Rabbit PA5-116785 Thermo Fisher Scientifics 1:1000
Histone H3 Rabbit 9715S Cell Signaling 1:10000
Vinculin Mouse 14-9777-82 Invitrogen 1:5000
HRP anti rabbit secondary Polyclonal NA934 GE healthcare lifesciences 1:10000
HRP anti mouse secondary Polyclonal NA931 GE healthcare lifesciences 1:10000

FACS:
Antibody Species Catalogue number Manufacturer Dilution
CD138 (281-2) Rat 142504 Biolegend 1:200
B220 (RA3-6B2) Rat 563793 BD Biosciences 1:200
IgG1 (A85-1) Rat 560089 BD Biosciences 1:200
IgM (II/41) Rat 553437 BD Biosciences 1:300
CD19 (1D3) Rat 115543 Biolegend 1:200

Validation

All antibodies used in immunofluorescence have distinct and well established signals/patterns and thus were easily validated by microscopy. Antibodies used in western blots were used in human cell extracts and validated by us using positive and negative controls or siRNA. Antibodies used for FACS are well established and validated by the manufacturers.

Eukaryotic cell lines

Policy information about cell lines and Sex and Gender in Research

Cell line source(s)	RPE-1 cells were obtained from Pellman Lab (HMS, USA).<br>RPE-1 expressing 53BP1-RFP and H2B-GFP were a kind gift from the Stojic group (BCI) and originally made by the Basto group (Institut Curie)<br>HEK-293M were obtained from Pellman Lab (HMS, USA).<br>U2OS were obtained at our institute from the lab of Sarah Martin (BCI-QMUL, UK).<br>HeLa were obtained at our institute from Roberto Bellelli's lab (BCI-QMUL, UK).
Authentication	RPE-1 cells have been previously authenticated by us by STR profiling.
Mycoplasma contamination	Mycoplasma testing is routinely tested in the lab (every 1-2 months). All cells lines used in this study were negative for mycoplasma
Commonly misidentified lines (See ICLAC register)	No common misindentified cells were used in this study.

Palaeontology and Archaeology

Specimen provenance	N/A
Specimen deposition	N/A
Dating methods	N/A

☐ Tick this box to confirm that the raw and calibrated dates are available in the paper or in Supplementary Information.

Ethics oversight	N/A

Note that full information on the approval of the study protocol must also be provided in the manuscript.

Animals and other research organisms

Policy information about studies involving animals; ARRIVE guidelines recommended for reporting animal research, and Sex and Gender in Research

Laboratory animals	Mice (Mus musculus, strain C57BL6) were used in this study to collect B cells from the spleen of either WT or ATAT1 KO mice. Animals 8-9 weeks old were used for WB while 21 weeks old animals were used for CSR.
Wild animals	not used in this study.
Reporting on sex	Sex was not considered as part for the study design but for consistency only males were used. Animal were used to collect B cells for further analyses in vitro.
Field-collected samples	not used in this study.
Ethics oversight	All mice were bred and maintained under specific-pathogen-free conditions at the Francis Crick Institute Biological Research Facility, in accordance with UK Home Office guidelines and approved by the Francis Crick Institute Ethical Review Panel.

Note that full information on the approval of the study protocol must also be provided in the manuscript.

Clinical data

Policy information about clinical studies

All manuscripts should comply with the ICMJE guidelines for publication of clinical research and a completed CONSORT checklist must be included with all submissions.

Clinical trial registration	not used in this study.
Study protocol	*Note where the full trial protocol can be accessed OR if not available, explain why.*
Data collection	*Describe the settings and locales of data collection, noting the time periods of recruitment and data collection.*
Outcomes	*Describe how you pre-defined primary and secondary outcome measures and how you assessed these measures.*

Dual use research of concern

Policy information about dual use research of concern

Hazards

Could the accidental, deliberate or reckless misuse of agents or technologies generated in the work, or the application of information presented in the manuscript, pose a threat to:

No	Yes	
☒	☐	Public health
☒	☐	National security
☒	☐	Crops and/or livestock
☒	☐	Ecosystems
☒	☐	Any other significant area

Experiments of concern

Does the work involve any of these experiments of concern:

No	Yes	
☒	☐	Demonstrate how to render a vaccine ineffective
☒	☐	Confer resistance to therapeutically useful antibiotics or antiviral agents
☒	☐	Enhance the virulence of a pathogen or render a nonpathogen virulent
☒	☐	Increase transmissibility of a pathogen
☒	☐	Alter the host range of a pathogen
☒	☐	Enable evasion of diagnostic/detection modalities
☒	☐	Enable the weaponization of a biological agent or toxin
☒	☐	Any other potentially harmful combination of experiments and agents

Plants

Seed stocks	N/A
Novel plant genotypes	N/A
Authentication	N/A

ChIP-seq

Data deposition

☐ Confirm that both raw and final processed data have been deposited in a public database such as GEO.

☐ Confirm that you have deposited or provided access to graph files (e.g. BED files) for the called peaks.

Data access links<br>*May remain private before publication.*	N/A
Files in database submission	N/A
Genome browser session<br>(e.g. UCSC)	N/A

Methodology

Replicates	N/A

Sequencing depth	N/A
Antibodies	N/A
Peak calling parameters	N/A
Data quality	N/A
Software	N/A

Flow Cytometry

Plots

Confirm that:

☒ The axis labels state the marker and fluorochrome used (e.g. CD4-FITC).

☒ The axis scales are clearly visible. Include numbers along axes only for bottom left plot of group (a 'group' is an analysis of identical markers).

☒ All plots are contour plots with outliers or pseudocolor plots.

☒ A numerical value for number of cells or percentage (with statistics) is provided.

Methodology

Sample preparation	All sample preparation details for flow cytometry are listed in the methods section under 'In vitro class switch recombination assay' and 'cell cycle analysis'
Instrument	Cell cycle analysis was performed using a Thermo Fisher Attune NXT and class switch recombination was performed using a BD Fortessa
Software	BD FACSDiva software and Attune NxT software were used to collect FACS data, and FlowJo v10 was used to analyze the FACS data.
Cell population abundance	For cell cycle analysis, at least 10 000 cells were analysed per condition.<br>For class-switch recombination, 150k live B cell were analysed per condition.
Gating strategy	For cell cycle analysis, Initial scatter-based gating was performed to remove cell debris: FSC-A versus SSC-A and doublets: FSC-A versus FSC-H. DNA content was based on the propidium iodide (PI) fluorescence signal: first high peak on the histogram represents G1 phase, second high peak, two times of the signal of the first peak, as G2/M phase, events between G1 and G2/M are classified as S phase.<br>For class-switch recombination, Gating strategy was set up as: lymphocytes->singlets->singlets->live (Zombie NIR negative)->non-PC (CD138-)->B cells (CD19+B220+) -> switched cells (IgG1+). The gates separating positive and negative cell populations were set at the midpoint on IgG1 expression between the two populations, with the positive population defined by high IgG1 expression. Gates were determined separately for each stimulation condition and timepoint and applied consistently across genotypes.<br>FFor DR-GFP assay, the analyses were performed with FlowJo software. First, I performed scatter-based gating to remove cell debris. I set a dot plot of Forward Scatter Area (FSC-A) versus Side Scatter Area (SSC-A) (FSC-A on the X-axis and SSC-A on the Y-axis) to visualize the general population of cells and exclude cell debris by selecting the population based on cell size. Next, I performed the other scatter-based gating to remove doublets by setting a dot plot of FSC-A versus FSC Height (FSC-H) (FSC-A on the X-axis and FSC-H on the Y-axis) and excluded the events that are with larger area from previous gated population. Then, I set a dot plot SSC-A versus mCherry Area (mCherry-A) (mCherry-A on the X-axis and SSC-A on the Y-axis) from the singlet population to gate mCherry positive population and exclude the mCherry negative population. From the mCherry positive population, I set a dot plot SSC-A versus GFP Area (GFP-A) (GFP-A on the X-axis and SSC-A on the Y-axis) to measure the percentage of GFP positive population in the mCherry positive population.

☐ Tick this box to confirm that a figure exemplifying the gating strategy is provided in the Supplementary Information.

Magnetic resonance imaging

Experimental design

Design type	N/A
Design specifications	N/A
Behavioral performance measures	N/A

Acquisition

Imaging type(s) | N/A

Field strength | N/A

Sequence & imaging parameters | N/A

Area of acquisition | N/A

Diffusion MRI ☐ Used ☐ Not used

Preprocessing

Preprocessing software | N/A

Normalization | N/A

Normalization template | N/A

Noise and artifact removal | N/A

Volume censoring | N/A

Statistical modeling & inference

Model type and settings | N/A

Effect(s) tested | N/A

Specify type of analysis: ☐ Whole brain ☐ ROI-based ☐ Both

Statistic type for inference | N/A

(See Eklund et al. 2016)

Correction | N/A

Models & analysis

n/a	Involved in the study
☐	☐ Functional and/or effective connectivity
☐	☐ Graph analysis
☐	☐ Multivariate modeling or predictive analysis

Functional and/or effective connectivity | N/A

Graph analysis | N/A

Multivariate modeling and predictive analysis | N/A
