# Supplementary material for: Tubulin acetylation regulates nuclear shape fluctuations and DNA repair
Source: Nat Cell Biol. 2026 Oct 28;28(11):2305–20. doi: 10.1038/s41556-026-02096-3 (PMC13751303; doi:10.1038/s41556-026-02096-3)
Supplement: Supplementary file 2 [file 41556_2026_2096_MOESM2_ESM.pdf]

nature portfolio

Peer Review File

Tubulin acetylation regulates nuclear shape fluctuations and DNA repair

Corresponding Author: Professor Susana Godinho

Version 0:

Decision Letter:

*Please delete the link to your author homepage if you wish to forward this email to co-authors.

Dear Professor Godinho,

Please accept our sincerest apologies for the length of time your manuscript has been under consideration at our journal. This is because we have been persistently chasing one referee (Reviewer #3, with expertise in microtubues) for their report but unfortunately we not yet received it. We will forward the report if we receive it in the future.

Your manuscript, "Nuclear shape fluctuations promote efficient DNA repair", has now been seen by 2 referees, who are experts in genome organization and nuclear envelope (referee 1); DNA repair (referee 2). As you will see from their comments (attached below) they find this work of potential interest, but have raised substantial concerns, which in our view would need to be addressed with considerable revisions before we can consider publication in Nature Cell Biology.

Nature Cell Biology editors discuss the referee reports in detail within the editorial team, including the chief editor, to identify key referee points that should be addressed with priority, and requests that are overruled as being beyond the scope of the current study. To guide the scope of the revisions, I have listed these points below. We are committed to providing a fair and constructive peer-review process, so please feel free to contact me if you would like to discuss any of the referee comments further.

In particular, it would be essential to:

A) Provide further experimental evidence regarding role of DSB mobility and repair efficiency downstream of MT acetylation (both Reviewers). Although Reviewer #1 recommends toning down of your claims, we consider these as central for consideration at our journal and would therefore require further experimental data.

B) Provide further experimental data to clarify mechanisms of DSB repair in your system (Reviewer #2)

C) All other referee concerns pertaining to strengthening existing data, providing controls, methodological details, clarifications and textual changes, should also be addressed.

D) Finally please pay close attention to our guidelines on statistical and methodological reporting (listed below) as failure to do so may delay the reconsideration of the revised manuscript. In particular please provide:

- a Supplementary Figure including unprocessed images of all gels/blots in the form of a multi-page pdf file. Please ensure that blots/gels are labeled and the sections presented in the figures are clearly indicated.

- a Supplementary Table including all numerical source data in Excel format, with data for different figures provided as different sheets within a single Excel file. The file should include source data giving rise to graphical representations and statistical descriptions in the paper and for all instances where the figures present representative experiments of multiple independent repeats, the source data of all repeats should be provided.

We would be happy to consider a revised manuscript that would satisfactorily address these points, unless a similar paper is published elsewhere, or is accepted for publication in Nature Cell Biology in the meantime.

When revising the manuscript please:

- ensure that it conforms to our format instructions and publication policies (see below and www.nature.com/nature/authors/).

- provide a point-by-point rebuttal to the full referee reports verbatim, as provided at the end of this letter.

- provide the completed Editorial Policy Checklist (found here https://www.nature.com/authors/policies/Policy.pdf), and Reporting Summary (found here https://www.nature.com/authors/policies/ReportingSummary.pdf). This is essential for reconsideration of the manuscript and these documents will be available to editors and referees in the event of peer

review. For more information see http://www.nature.com/authors/policies/availability.html or contact me.

Nature Cell Biology is committed to improving transparency in authorship. As part of our efforts in this direction, we are now requesting that all authors identified as 'corresponding author' on published papers create and link their Open Researcher and Contributor Identifier (ORCID) with their account on the Manuscript Tracking System (MTS), prior to acceptance. ORCID helps the scientific community achieve unambiguous attribution of all scholarly contributions. You can create and link your ORCID from the home page of the MTS by clicking on 'Modify my Springer Nature account'. For more information please visit please visit www.springernature.com/orcid.

Please submit the revised manuscript files and the point-by-point rebuttal to the referee comments using this link:

Link Redacted

*This url links to your confidential home page and associated information about manuscripts you may have submitted or be reviewing for us. If you wish to forward this email to co-authors, please delete the link to your homepage.

We would like to receive a revised submission within six months. We would be happy to consider a revision even after this timeframe, however if the resubmission deadline is missed and the paper is eventually published, the submission date will be the date when the revised manuscript was received.

We hope that you will find our referees' comments, and editorial guidance helpful. Please do not hesitate to contact me if there is anything you would like to discuss.

Best wishes,

Daryl

Daryl Jason Verzosa David, PhD

Senior Editor, Nature Cell Biology
Advisory Editor, npj Biological Physics and Mechanics
Nature Portfolio

Heidelberger Platz 3, 14197 Berlin, Germany
Email: daryl.david@nature.com
ORCID: https://orcid.org/0000-0002-9253-4805

Reviewers' Comments:

Reviewer #1 (Remarks to the Author):

Wallis et al. outline a mechanism by which DNA damage induces acetylation of microtubules (MT), which in turn alters vimentin localization around the nucleus, leading to nuclear deformation and increased mobility of double strand breaks (DSBs). The authors propose that the increased mobility of DSBs enhances their repair efficiency. The authors present a set of experiments, many using knockout or overexpression of the microtubule acetyltransferase aTAT1, to support these various steps including demonstration of increased MT acetylation, mislocaliztion of vimentin, increased DSB mobility. They also present data to suggest that MT acetylation promotes repair by homologous recombination but not by non-homologous end joining (NHEJ).

This is a perfectly plausible model and the data generally supports it. The presented data is of good technical quality and the study is carefully designed with many important controls included. While many conclusions rely on correlations, they all seem to hold when cross-compared, which is reassuring. The demonstration of relevance for HR but not for NHEJ is also an important point.

There are some general points that need clarification:
The acetylation of MT is rather slow and appears to peak around 48-72 hours after DNA damage. However, repair would be expected to occur faster or at least the homologue partners search for which DSB mobility is important would be expected to occur earlier. What is the point of maximizing acMT at a time when most repair events have occurred? The apparent discrepancy between repair kinetics and acMT puts in questions the functional relevance of acMT for repair efficiency. The authors partially address this issue (lines 270-297) but a more direct demonstration of the repair dynamics of breaks via HR is required to reconcile the slow time course of MT acetylation and its proposed role in HR. Can the authors provide additional data to support their claim?
It is difficult to know whether the observed change of DSB mobility is merely a bystander effect or represents an active defense mechanism. I would not expect the authors to resolve this but toning down statements regarding directed causality and a frank discussion of these two scenarios should be included.

Although the authors observe a correlation between nuclear shape fluctuations, DSB mobility and repair efficiency, they do not directly show that it is the change in nuclear shape fluctuations or altered mobility that contributes to delays in repair. These are merely correlations. In the absence of more direct evidence for a causal effect, for example, by altering mobility of DSBs via tethering or alike, this claim should be toned down.

Related to the above point, I found the title of the manuscript not entirely appropriate as it highlights one of the weakest conclusions of the study. My recommendation would be to highlight in the title the role of MT acetylation in DNA repair since this is a stronger conclusion

and make the changes in cellular and nuclear morphology a secondary conclusion.

Additional technical points:
The authors show in figure 1 increased MT acetylation after DNA damage using imaging. These data should be validated by Western blotting.

I did not understand the rationale for the experiment in which aTAT is co-depleted with vimentin. Why would loss of vimentin restore DSB mobility and repair efficiency? Please clarify.

Reviewer #1 (Remarks on Protocol(s)):

they seem appropriate

Reviewer #2 (Remarks to the Author):

Wallis et al: Nuclear shape fluctuations promote efficient DNA repair

This is a new submission from the Godinho lab that presents a range of novel observations regarding how changes in nuclear shape impact DNA repair and DNA damage foci outcomes. They focus on acetylated tubulin, which is mediated by TAT1. Overall, this is a beautiful data set that warrants publication in NCB with some additional clarifying experiments.
In Figure 1, it is demonstrated that in response to cisplatin, doxorubicin, or IR, 53BP1 foci forms and the initial kinetics to some degree correlate with the appearance of Ac-tubulin. Interestingly, however, while 53BP1 foci are eventually resolved largely by 24 hours post IR, AC-tubulin continues to dramatically increase up to 72 hours post IR, and doesn't start to decrease until 96 hours. Nice videos are provided that demonstrate 53bp1 foci that 'wiggle', and this movement is abrogated by loss of TAT1, ie Ac-tubulin, or nocodazole.
Figure 2 shows that the nuclear periphery also moves and correlates with foci movements and are dependent on centriole proximity. Moreover, TAT1 siRNA abrogates this.
Figure 3 shows that the function of Ac-tubulin is to shift vimentin away from the nuclear periphery so to reduce the rigidity of the nuclear envelope. The effects of TAT1 siRNA are abrogated by combining with vimentin siRNA, supporting the role of Ac-Tublin in shifting vimentin.
Figure 4 begins to assess the consequences of disrupting TAT1 on DNA repair outcomes. They show that 53bp1 foci are not resolved using cisplatin or doxorubicin with TAT1 siRNA.
Figure 5 explores additional aspects of DNA repair, including H2AX and RAD51 foci. Confusingly, most of the data is with cisplatin, but then when they come to assess RAD51 foci they switch to IR, and the results don't correlate as well compared with 53bp1 and h2ax foci in terms of the degree of effect by ATAT1 KO. RAD51 foci need to be assessed in response to cisplatin in this context. While there is a bigger difference when assessing puncta versus filaments, the significance of these are unclear.
Figure 6 reports and interesting phenotype of larger foci begining to appear longer term that fuse and travel to the nuclear periphery and induce senescence in ATAT1 KO cells.

Issues that should be addressed are mostly in regard to the DNA repair aspect of the work.

Specifically:
1. what is the DNA repair defect when ac-tubulin is absent? The defect in RAD51 foci appear minor, the phenotypes appear to be more dramatic with cisplatin, and it would be important to assess RAD51 foci in the same manner as H2AX in Figure 5b.
2. The conclusion made in regard to the impact of Ac-tubulin on DSB pathway choice are not sufficiently supported. The authors should take note that 53BP1 foci do not correspond to NHEJ repair efficiency. This is a common misconception. Rather, 53BP1 foci simply mark H2AX-ubiquitin and DSBs. They will also find BRCA1 and RAD51 foci co-localizing here. Therefore, statements about 53bp1 foci and NHEJ need to be modified.
3. Overall, it is not clear what Ac-tubulin is doing to promote DSB repair. If HR is affected, the authors need to prove this, for example, PARPi sensitivity assays would be useful, HR reporters could be used.
4. If the 'wiggle' is specific to HR but not NHEJ it could be assessed by synchronizing cells using the CDK4 inhibitor palbociclib and subjecting to IR in G1 (NHEJ only) vs S (HR mostly) phase released cells to observe repair specific differences. One would predict no wiggle in G1 cells post IR?
5. A confusing result is the time course that it takes for ac-tubulin to return to basal levels compared with the time it takes for 53bp1 foci to return. One would expect that cells have completed one or two mitoses by 96 hours. Could the authors confirm this and consider the impact of ac-tubulin and cell cycle progression.
6. Figure 6D, why are most foci able to resolve but only a small number not resolving?
7. The project uses one cell line throughout. The primary findings should be confirmed in at least two other cell lines.
8. Colony formation assays using WT and TAT1 KO cells for cisplatin, IR, doxorubicin, PARPi sensitivity should be included.

Reviewer #3 (Remarks to the Author):

In this manuscript the authors advance a model whereby tubulin acetylation is directly responsible for effects on DNA damage repair through a mechanism that is not very clear but might involve an effect on the mechanical forces experienced by the nucleus. The hypothesis presented is interesting and there is an increased interest into the role of force in DNA damage, but the study feels preliminary and needs additional orthogonal investigations to support the model presented. The authors use primarily imaging to address the question by documenting an increase in microtubule acetylation after treatment with cisplatin or irradiation, concomitant with a change in the mobility of DNA damage foci. One concern I have is that the effects that they see on tubulin acetylation are in fact a more general stress response due to cisplatin exposure or irradiation. Likewise, I am not convinced that the mechanical forces exerted by the microtubules do in fact affect DNA repair directly. Cisplatin is highly reactive and has been shown to affect many proteins and RNA. Similarly, radiation exposure will damage other components and not DNA alone.

As far as presentation, the manuscript is well written and the figures are high quality and well presented. Specific comments below that I hope the authors will take into consideration.

In Fig. 2h the response to nocodazole treatment is very close to that for Tat depletion. This is surprising since acetylated MTs are known to be resistant to nocodazole (one of the ways they were classified as stable). Are the acetylated microtubules lost during nocodazole treatment?

Taxol will stabilize microtubules and induce acetylation. What happens if you add taxol which promotes MT acetylation? Is there a suppression of oscillations? With downstream effects on DNA repair?

Doe a kinesin inhibitor have a similar effect as the kif5b depletion? In other words is the role of kinesin in transport or structural for the effects that they are seeing? Small point but since this is stated in the introduction, it has now been shown by several labs that kinesin is not sensitive to acetylation but that the increased activity of kinesin on acetylated

microtubules is due to the fact that bundled microtubules are also acetylated and bundled microtubule promote increased motility.

Figure 1i – the MSD plot. Please show MSD versus time and not on a log scale. What is the confidence interval/SD for these MSD traces?

The section on lines 213-220 is highly questionable. Of course, AlphaFold3 will predict a strong interaction with a-tubulin! Structures of TAT with a-tubulin are in the literature for years and so AlphaFold is heavily biased towards these as they are in the training database. AlphaFold cannot be used to conclude that an enzyme does not modify other targets. If the authors are concerned about other aTAT targets, as one should always be with an enzyme, this is not going to convince many. A better way is to have more data for the mechanism presented.

Figure 3d and e – right most graph. What is the y axis? Is it really fluctuations that are plotted here?

How were the cisplatin incubation time and washout chosen? are the kinetics on MT acetylation and vimentin displacement slower at lower concentrations? are they visible at all? cisplatin makes covalent interactions with its target and has been known to crosslink proteins, so even after the washout cisplatin adducts would remain. What is the rationale for the time windows used? Did the authors perform ICP-MS to analyze platinum in their samples?

The primary measure of DNA damage presented is through 53BP1 foci and H2AX imaging. Is for example DDB recruitment to damage sites also changed in the TAT KD?

Is vimentin displacement due to the direct effects of the cytotoxic treatment on this filament?

Can the authors confirm their findings in another cell line? How is this response in cells that have a large amount of acetylated tubulin to begin with? Do they have different rates of repair? This would seem relevant in terms of in vivo sensitivity to DNA damage.

Figure 5c- how were Rad51 filaments quantified? The images presented are not convincing that they can distinguish between filaments versus puncta. Higher resolution images will be required to make this assessment and an objective, quantitative criteria needed on how these two states are classified.

=============

GUIDELINES FOR MANUSCRIPT SUBMISSION TO NATURE CELL BIOLOGY

READABILITY OF MANUSCRIPTS – Nature Cell Biology is read by cell biologists from diverse backgrounds, many of whom are not native English speakers. Authors should aim to communicate their findings clearly, explaining technical jargon that might be unfamiliar to

non-specialists, and avoiding non-standard abbreviations. Titles and abstracts should concisely communicate the main findings of the study, and the background, rationale, results and conclusions should be clearly explained in the manuscript in a manner accessible to a broad cell biology audience. Nature Cell Biology uses British spelling.

MANUSCRIPT FORMAT – please follow the guidelines listed in our Guide to Authors regarding manuscript formats at Nature Cell Biology.

TITLE – should be no more than 100 characters including spaces, without punctuation and avoiding technical terms, abbreviations, and active verbs..

AUTHOR NAMES – should be given in full.

AUTHOR AFFILIATIONS – should be denoted with numerical superscripts (not symbols) preceding the names. Full addresses should be included, with US states in full and providing zip/post codes. The corresponding author is denoted by: "Correspondence should be addressed to [initials]."

ABSTRACT AND MAIN TEXT – please follow the guidelines that are specific to the format of your manuscript, as listed in our Guide to Authors (http://www.nature.com/ncb/pdf/ncb_gta.pdf) Briefly, Nature Cell Biology Articles, Resources and Technical Reports have 3500 words, including a 150 word abstract, and the main text is subdivided in Introduction, Results, and Discussion sections. Nature Cell Biology Letters have up to 2500 words, including a 180 word introductory paragraph (abstract), and the text is not subdivided in sections.

ACKNOWLEDGEMENTS – should be kept brief. Professional titles and affiliations are unnecessary. Grant numbers can be listed.

AUTHOR CONTRIBUTIONS – must be included after the Acknowledgements, detailing the contributions of each author to the paper (e.g. experimental work, project planning, data analysis etc.). Each author should be listed by his/her initials.

FINANCIAL AND NON-FINANCIAL COMPETING INTERESTS – the authors must include one of three declarations: (1) that they have no financial and non-financial competing interests; (2) that they have financial and non-financial competing interests; or (3) that they decline to respond, after the Author Contributions section. This statement will be published with the article, and in cases where financial and non-financial competing interests are declared, these will be itemized in a web supplement to the article. For further details please see https://www.nature.com/licenceforms/nrg/competing-interests.pdf.

REFERENCES – are limited to a total of 70 for Articles, Resources, Technical Reports; and 40 for Letters. This includes references in the main text and Methods combined. References must be numbered sequentially as they appear in the main text, tables and figure legends and Methods and must follow the precise style of Nature Cell Biology references. References only cited in the Methods should be numbered consecutively following the last reference cited in the main text. References only associated with Supplementary Information (e.g. in supplementary legends) do not count toward the total reference limit and do not need to be cited in numerical continuity with references in the main text. Only published papers can be cited, and each publication cited should be included in the numbered reference list, which should include the manuscript titles. Footnotes are not permitted.

METHODS – Nature Cell Biology publishes methods online. The methods section should be provided as a separate Word document, which will be copyedited and appended to the manuscript PDF, and incorporated within the HTML format of the paper.

Methods should be written concisely, but should contain all elements necessary to allow interpretation and replication of the results. As a guideline, Methods sections typically do not exceed 3,000 words. The Methods should be divided into subsections listing reagents and techniques. When citing previous methods, accurate references should be provided and any alterations should be noted. Information must be provided about: antibody dilutions, company names, catalogue numbers and clone numbers for monoclonal antibodies; sequences of RNAi and cDNA probes/primers or company names and catalogue numbers if reagents are commercial; cell line names, sources and information on cell line identity and authentication. Animal studies and experiments involving human subjects must be reported in detail, identifying the committees approving the protocols. For studies involving human subjects/samples, a statement must be included confirming that informed consent was obtained. Statistical analyses and information on the reproducibility of experimental results should be provided in a section titled "Statistics and Reproducibility".

All Nature Cell Biology manuscripts submitted on or after March 21 2016 must include a Data availability statement at the end of the Methods section. For Springer Nature policies on data availability see http://www.nature.com/authors/policies/availability.html; for more information on this particular policy see http://www.nature.com/authors/policies/data/data-availability-statements-data-citations.pdf. The Data availability statement should include:

• Accession codes for primary datasets (generated during the study under consideration and designated as "primary accessions") and secondary datasets (published datasets reanalysed during the study under consideration, designated as "referenced accessions"). For primary accessions data should be made public to coincide with publication of the manuscript. A list of data types for which submission to community-endorsed public repositories is mandated (including sequence, structure, microarray, deep sequencing data) can be found here http://www.nature.com/authors/policies/availability.html#data.

• Unique identifiers (accession codes, DOIs or other unique persistent identifier) and hyperlinks for datasets deposited in an approved repository, but for which data deposition is not mandated (see here for details http://www.nature.com/sdata/data-policies/repositories).

• At a minimum, please include a statement confirming that all relevant data are available from the authors, and/or are included with the manuscript (e.g. as source data or supplementary information), listing which data are included (e.g. by figure panels and data types) and mentioning any restrictions on availability.

• If a dataset has a Digital Object Identifier (DOI) as its unique identifier, we strongly encourage including this in the Reference list and citing the dataset in the Methods.

We recommend that you upload the step-by-step protocols used in this manuscript to protocols.io. More details can found at https://www.protocols.io/help/publish-articles.

DISPLAY ITEMS – main display items are limited to 6-8 main figures and/or main tables for Articles, Resources, Technical Reports; and 5 main figures and/or main tables for Letters. For Supplementary Information see below.

FIGURES – Colour figure publication costs $600 for the first, and $300 for each subsequent colour figure. All panels of a multi-panel figure must be logically connected and arranged as they would appear in the final version. Unnecessary figures and figure panels should be avoided (e.g. data presented in small tables could be stated briefly in the text instead).

All imaging data should be accompanied by scale bars, which should be defined in the legend.
Cropped images of gels/blots are acceptable, but need to be accompanied by size markers, and to retain visible background signal within the linear range (i.e. should not be saturated). The boundaries of panels with low background have to be demarked with black lines. Splicing of panels should only be considered if unavoidable, and must be clearly marked on the figure, and noted in the legend with a statement on whether the samples were obtained and processed simultaneously. Quantitative comparisons between samples on different gels/blots are discouraged; if this is unavoidable, it should only be performed for samples derived from the same experiment with gels/blots were processed in parallel, which needs to be stated in the legend.

Figures should be provided at approximately the size that they are to be printed at (single column is 86 mm, double column is 170 mm) and should not exceed an A4 page (8.5 x 11"). Reduction to the scale that will be used on the page is not necessary, but multi-panel figures should be sized so that the whole figure can be reduced by the same amount at the smallest size at which essential details in each panel are visible. In the interest of our colour-blind readers we ask that you avoid using red and green for contrast in figures. Replacing red with magenta and green with turquoise are two possible colour-safe alternatives. Lines with widths of less than 1 point should be avoided. Sans serif typefaces, such as Helvetica (preferred) or Arial should be used. All text that forms part of a figure should be rewritable and removable.

We accept files from the following graphics packages in either PC or Macintosh format:

- For line art, graphs, charts and schematics we prefer Adobe Illustrator (.AI), Encapsulated PostScript (.EPS) or Portable Document Format (.PDF). Files should be saved or exported as such directly from the application in which they were made, to allow us to restyle them according to our journal house style.

- We accept PowerPoint (.PPT) files if they are fully editable. However, please refrain from adding PowerPoint graphical effects to objects, as this results in them outputting poor quality raster art. Text used for PowerPoint figures should be Helvetica (preferred) or Arial.

- We do not recommend using Adobe Photoshop for designing figures, but we can accept Photoshop generated (.PSD or .TIFF) files only if each element included in the figure (text, labels, pictures, graphs, arrows and scale bars) are on separate layers. All text should be editable in 'type layers' and line-art such as graphs and other simple schematics should be preserved and embedded within 'vector smart objects' - not flattened raster/bitmap graphics.

- Some programs can generate Postscript by 'printing to file' (found in the Print dialogue). If using an application not listed above, save the file in PostScript format or email our Art Editor, Allen Beattie for advice (a.beattie@nature.com).

Regardless of format, all figures must be vector graphic compatible files, not supplied in a flattened raster/bitmap graphics format, but should be fully editable, allowing us to highlight/copy/paste all text and move individual parts of the figures (i.e. arrows, lines, x and y axes, graphs, tick marks, scale bars etc.). The only parts of the figure that should be in pixel raster/bitmap format are photographic images or 3D rendered graphics/complex technical illustrations.

All placed images (i.e. a photo incorporated into a figure) should be on a separate layer and independent from any superimposed scale bars or text. Individual photographic images must be a minimum of 300+ DPI (at actual size) or kept constant from the original picture acquisition and not decreased in resolution post image acquisition. All colour artwork should be RGB format.

FIGURE LEGENDS – must not exceed 350 words for each figure to allow fit on a single printed NCB page together with the figure. They must include a brief title for the whole figure, and short descriptions of each panel with definitions of the symbols used, but without detailing methodology.

TABLES – main tables should be provided as individual Word files, together with a brief title and legend. For supplementary tables see below.

SUPPLEMENTARY INFORMATION – Supplementary information is material directly relevant to the conclusion of a paper, but which cannot be included in the printed version in order to keep the manuscript concise and accessible to the general reader. Supplementary information is an integral part of a Nature Cell Biology publication and should be prepared and presented with as much care as the main display item, but it must not include non-essential data or text, which may be removed at the editor's discretion. All supplementary material is fully peer-reviewed and published online as part of the HTML version of the manuscript. Supplementary Figures and Supplementary Notes are appended at the end of the main PDF of the published manuscript.

Supplementary items should relate to a main text figure, wherever possible, and should be mentioned sequentially in the main manuscript, designated as Supplementary Figure, Table, Video, or Note, and numbered continuously (e.g. Supplementary Figure 1, Supplementary Figure 2, Supplementary Table 1, Supplementary Table 2 etc.).

Unprocessed scans of all key data generated through electrophoretic separation techniques need to be presented in a supplementary figure that should be labelled and numbered as the final supplementary figure, and should be mentioned in every relevant figure legend.

This figure does not count towards the total number of figures and is the only figure that can be displayed over multiple pages, but should be provided as a single file, in PDF or TIFF format. Data in this figure can be displayed in a relatively informal style, but size markers and the figures panels corresponding to the presented data must be indicated.

The total number of Supplementary Figures (not including the “unprocessed scans” Supplementary Figure) should not exceed the number of main display items (figures and/or tables (see our Guide to Authors and March 2012 editorial http://www.nature.com/ncb/authors/submit/index.html#suppinfo; http://www.nature.com/ncb/journal/v14/n3/index.html#ed). No restrictions apply to Supplementary Tables or Videos, but we advise authors to be selective in including supplemental data.

Each Supplementary Figure should be provided as a single page and as an individual file in one of our accepted figure formats and should be presented according to our figure guidelines (see above). Supplementary Tables should be provided as individual Excel files. Supplementary Videos should be provided as .avi or .mov files up to 50 MB in size. Supplementary Figures, Tables and Videos much be accompanied by a separate Word document including titles and legends.

GUIDELINES FOR EXPERIMENTAL AND STATISTICAL REPORTING

REPORTING REQUIREMENTS – To improve the quality of methods and statistics reporting in our papers we have recently revised the reporting checklist we introduced in 2013. We are now asking all life sciences authors to complete two items: an Editorial Policy Checklist (found here https://www.nature.com/authors/policies/Policy.pdf) that verifies compliance with all required editorial policies and a reporting summary (found here https://www.nature.com/authors/policies/ReportingSummary.pdf) that collects information on experimental design and reagents. These documents are available to referees to aid the evaluation of the manuscript. Please note that these forms are dynamic ‘smart pdfs’ and must therefore be downloaded and completed in Adobe Reader. We will then flatten them for ease of use by the reviewers. If you would like to reference the guidance text as you complete the template, please access these flattened versions at http://www.nature.com/authors/policies/availability.html.

STATISTICS – Wherever statistics have been derived the legend needs to provide the n number (i.e. the sample size used to derive statistics) as a precise value (not a range), and define what this value represents. Error bars need to be defined in the legends (e.g. SD, SEM) together with a measure of centre (e.g. mean, median). Box plots need to be defined in terms of minima, maxima, centre, and percentiles. Ranges are more appropriate than standard errors for small data sets. Wherever statistical significance has been derived, precise p values need to be provided and the statistical test used needs to be stated in the legend. Statistics such as error bars must not be derived from n<3. For sample sizes of n<5 please plot the individual data points rather than providing bar graphs. Deriving statistics from technical replicate samples, rather than biological replicates is strongly discouraged. Wherever statistical significance has been derived, precise p values need to be provided and the statistical test stated in the legend.

Information on how many times each experiment was repeated independently with similar results needs to be provided in the legends and/or Methods for all experiments, and in particular wherever representative experiments are shown.

We strongly recommend the presentation of source data for graphical and statistical analyses as a separate Supplementary Table, and request that source data for all independent repeats are provided when representative experiments of multiple independent repeats, or averages of two independent experiments are presented. This supplementary table should be in Excel format, with data for different figures provided as different sheets within a single Excel file. It should be labelled and numbered as one of the supplementary tables, titled “Statistics Source Data”, and mentioned in all relevant figure legends.

--------- Please don't hesitate to contact NCB@nature.com should you have queries about any of the above requirements ---------

Version 1:

Decision Letter:

Our ref: NCB-A59743A

30th July 2026

Dear Dr. Godinho,

I once again apologize for the delay. As mentioned previously, we had asked for Reviewer #2 to comment on Reviewer #1's current report.

Thank you for submitting your revised manuscript "Tubulin acetylation regulates nuclear shape fluctuations to promote efficient DNA repair" (NCB-A59743A). It has now been seen by the original referees and their comments are below. The reviewers find that the paper has improved in revision, and therefore we'll be happy in principle to publish it in Nature Cell Biology, pending minor revisions to satisfy the referees' final requests and to comply with our editorial and formatting guidelines.

In particular, we would require further toning down of the conclusions in light with Reviewer #1 and #2's current comments as well as those of Reviewer #3; which may include edits to your title and text throughout.

We are now performing detailed checks on your paper and will send you a checklist detailing our editorial and formatting requirements in a couple of weeks. Please do not upload the final materials and make any revisions until you receive this additional information from us.

Thank you again for your interest in Nature Cell Biology Please do not hesitate to contact me if you have any questions.

Sincerely,
Daryl

Daryl Jason Verzosa David, PhD

Senior Editor, Nature Cell Biology
Advisory Editor, npj Biological Physics and Mechanics
Nature Portfolio

Heidelberger Platz 3, 14197 Berlin, Germany
Email: daryl.david@nature.com
ORCID: https://orcid.org/0000-0002-9253-4805

Reviewer #1 (Remarks to the Author):

Wallis et al. in this revised manuscript have addressed most of my technical points to a satisfactory degree.

However, the most important issue of the mechanistic basis for the effect of MT acetylation on DSB mobility has not been resolved. It remains unclear how MT-acetylation communicates with chromatin to alter DSB mobility; this is a central element of the proposed model but is not demonstrated. The authors' suggestion that this occurs via changes to nuclear shape is not convincing nor very plausible. It is difficult to envision how large scale changes in nuclear shape promote changes in the mobility of specific loci, especially given that the authors propose that this mechanism is of relevance for persistent unrepaired DNA breaks. The model in figure 8 does not illustrate a mechanism and no experiments to directly test this major conclusion and key step in the model (as mentioned in the title) are presented. One way to address this would be to manipulate nuclear shape by mechanical means and demonstrated resolution of persistent DSBs. In the absence of such experiments, the study remains purely phenomenological and the major central mechanistic conclusion of a role of nuclear shape in promoting DSB mobility is not supported.

Reviewer #2 (Remarks to the Author):

This is an outstanding study and much improved.

Reviewer #2's additional comments on Reviewer #1's current concerns:
I agree to some extent with Reviewer 1's comments. However, these points do not detract from the quality of the work or the important new insights presented in this study. Although the manuscript does not directly address the mechanistic impact of nuclear shape on DSB repair, I think this would be a challenging—and perhaps not currently feasible—question to address experimentally, as manipulating nuclear shape is likely to have numerous unintended consequences.

I believe the issues raised by Reviewer 1 could be acknowledged in the Discussion and proposed as important directions for future studies. Nevertheless, the authors convincingly demonstrate a novel and compelling connection between these phenomena, providing biologically relevant insights into the events that occur during DSB repair. Therefore, I strongly urge Nature Cell Biology to accept this manuscript in its current form.

Overall, in my opinion, the approaches used are appropriate and sufficient to support the conclusions. The authors just need to acknowledge the limitation mentioned by R1.

Reviewer #3 (Remarks to the Author):

The authors have overall addressed my concerns.

As they revise their manuscript, I hope that the authors will also consider the fact that cisplatin (Horibata et al /PMID42241284), IR (Zamansky et al./PMID1576000, Goldblum et al/PMID34343476) and doxorubicin (Rabkin et al. PMID3325651), all agents used in this study to induce DNA damage also act directly on microtubules by destabilizing them and inducing damage. These microtubule abnormalities persist long after the damaging agent has been removed, with kinetics not dissimilar to what the authors describe as the kinetics of tubulin acetylation. The authors should mention this in their discussion as it could provide another axis that connects the damage agents to MT effects, changes in acetylation followed by feedback on repair (and possibly add confounding factors to the data interpretation).

Fig 5c-f data are a nice addition

Small point: In Discussion it is stated: "This mechanism reveals an unexpected role for cytoplasmic microtubules in reshaping nuclear mechanics and broadens current models of how spatial dynamics contribute to genome maintenance" The authors should consider rephrasing this – there is nothing unexpected about the role of cytoplasmic MTs in affecting nuclear mechanics. Here is a recent review on this very topic https://www.sciencedirect.com/science/article/pii/S0962892421000702. This statement needs to be more focused to cover what is novel in their study.

Reviewer #3 (Remarks on Protocol(s)):

Methods are sufficiently well described.

INTERNAL USE ONLY AIP modules required: ["Clinical","Life Science"]

Version 2:

Decision Letter:

Dear Dr Godinho,

I am pleased to inform you that your manuscript, "Tubulin acetylation regulates nuclear shape fluctuations and DNA repair", has now been accepted for publication in Nature Cell Biology.

Thank you for sending us the final manuscript files to be processed for print and online production, and for returning the manuscript checklists and other forms. Your manuscript will now be passed to our production team who will be in contact with you if there are any questions with the production quality of supplied figures and text.

Over the next few weeks, your paper will be copyedited to ensure that it conforms to Nature Cell Biology style. Once your paper is typeset, you will receive an email with a link to choose the appropriate publishing options for your paper and our Author Services team will be in touch regarding any additional information that may be required.

After the grant of rights is completed, you will receive a link to your electronic proof via email with a request to make any corrections within 48 hours. If, when you receive your proof, you cannot meet this deadline, please inform us at rjsproduction@springernature.com immediately.

You will not receive your proofs until the publishing agreement has been received through our system.

Due to the importance of these deadlines, we ask that you please let us know now whether you will be difficult to contact over the next month. If this is the case, we ask you provide us with the contact information (email, phone and fax) of someone who will be able to check the proofs on your behalf, and who will be available to address any last-minute problems.

If you have any questions about our publishing options, costs, Open Access requirements, or our legal forms, please contact ASJournals@springernature.com

Once your paper has been scheduled for online publication, the Nature press office will be in touch to confirm the details. An online order form for reprints of your paper is available at https://www.nature.com/reprints/author-reprints.html. All co-authors, authors' institutions and authors' funding agencies can order reprints using the form appropriate to their geographical region.

Publication is conditional on the manuscript not being published elsewhere and on there being no announcement of this work to any media outlet until the online publication date in Nature Cell Biology.

You may wish to make your media relations office aware of your accepted publication, in case they consider it appropriate to organize some internal or external publicity. Once your paper has been scheduled you will receive an email confirming the publication details. This is normally 3-4 working days in advance of publication. If you need additional notice of the date and time of publication, please let the production team know when you receive the proof of your article to ensure there is sufficient time to coordinate. Further information on our embargo policies can be found here: https://www.nature.com/authors/policies/embargo.html

Please note that *Nature Cell Biology* is a Transformative Journal (TJ). Authors may publish their research with us through the traditional subscription access route or make their paper immediately open access through payment of an article-processing charge (APC). Authors will not be required to make a final decision about access to their article until it has been accepted. Find out more about Transformative Journals

Authors may need to take specific actions to achieve compliance with funder and institutional open access mandates. If your research is supported by a funder that requires immediate open access (e.g. according to Plan S principles or the NIH public access policy) then you should select the gold OA route, and we will direct you to the compliant route where possible. Because authors warrant under our subscription licensing terms that they haven't committed to licensing any version of their article under a licence inconsistent with the terms of our agreement – including the applicable embargo period – publication under the subscription model isn't suitable for authors whose funders require no embargo.

To assist our authors in disseminating their research to the broader community, our SharedIt initiative provides you with a unique shareable link that will allow anyone (with or without a subscription) to read the published article. Recipients of the link with a subscription will also be able to download and print the PDF.

If your paper includes color figures, please be aware that in order to help cover some of the additional cost of four-color reproduction,

Nature Portfolio charges our authors a fee for the printing of their color figures. Please contact our offices for exact pricing and details.

As soon as your article is published, you will receive an automated email with your shareable link.

If you have not already done so, we strongly recommend that you upload the step-by-step protocols used in this manuscript to protocols.io (https://protocols.io), an open online resource that allows researchers to share their detailed experimental know-how. All uploaded protocols are made freely available and are assigned DOIs for ease of citation. Protocols and Nature Portfolio journal papers in which they are used can be linked to one another, and this link is clearly and prominently visible in the online versions of both. Authors who performed the specific experiments can act as primary authors for the Protocol as they will be best placed to share the methodology details, but the Corresponding Author of the present research paper should be included as one of the authors. By uploading your Protocols onto protocols.io, you are enabling researchers to more readily reproduce or adapt the methodology you use, as well as increasing the visibility of your protocols and papers. You can also establish a dedicated workspace to collect your lab Protocols. Further information can be found at https://www.protocols.io/help/publish-articles.

You can use a single sign-on for all your accounts, view the status of all your manuscript submissions and reviews, access usage statistics for your published articles and download a record of your refereeing activity for the Nature Portfolio.

Nature Cell Biology encourages authors presenting evidence for cell, biological, molecular, and genetic interactions to consider communicating these findings using Biofactoid (https://biofactoid.org/). This tool helps users share a searchable representation of interactions (e.g. binding, gene expression, post-translational modification) between genes, gene products, or chemicals. Information added to Biofactoid, with author attribution, is shared on social media and public databases, such as Pathway Commons, where it can be discovered and analyzed in the context of a large and growing corpus of knowledge.

Please feel free to contact us if you have any questions.

With kind regards,

Daryl

Daryl Jason Verzosa David, PhD

Senior Editor, Nature Cell Biology
Advisory Editor, npj Biological Physics and Mechanics
Nature Portfolio

Heidelberger Platz 3, 14197 Berlin, Germany
Email: daryl.david@nature.com
ORCID: https://orcid.org/0000-0002-9253-4805

Click here if you would like to recommend Nature Cell Biology to your librarian
http://www.nature.com/subscriptions/recommend.html#forms

** Visit the Springer Nature Editorial and Publishing website at www.springernature.com/editorial-and-publishing-jobs for more information about our career opportunities. If you have any questions please click here.**

Open Access This Peer Review File is licensed under a Creative Commons Attribution 4.0 International License, which permits use, sharing, adaptation, distribution and reproduction in any medium or format, as long as you give appropriate credit to the original author(s) and the source, provide a link to the Creative Commons license, and indicate if changes were made.
In cases where reviewers are anonymous, credit should be given to 'Anonymous Referee' and the source.
The images or other third party material in this Peer Review File are included in the article's Creative Commons license, unless indicated otherwise in a credit line to the material. If material is not included in the article's Creative Commons license and your intended use is not permitted by statutory regulation or exceeds the permitted use, you will need to obtain permission directly from the copyright holder.
To view a copy of this license, visit https://creativecommons.org/licenses/by/4.0/

We thank the reviewers for their careful evaluation of our manuscript and for their constructive and insightful comments. We have addressed all concerns raised and are confident that the extensive new data and revisions substantially strengthen both the mechanistic depth and the overall conclusions of the manuscript.

REVIEWER #1

Wallis et al. outline a mechanism by which DNA damage induces acetylation of microtubules (MT), which in turn alters vimentin localization around the nucleus, leading to nuclear deformation and increased mobility of double strand breaks (DSBs). The authors propose that the increased mobility of DSBs enhances their repair efficiency. The authors present a set of experiments, many using knockout or overexpression of the microtubule acetyltransferase aTAT1, to support these various steps including demonstration of increased MT acetylation, mislocaliztion of vimentin, increased DSB mobility. They also present data to suggest that MT acetylation promotes repair by homologous recombination but not by non-homologous end joining (NHEJ).

This is a perfectly plausible model and the data generally supports it. The presented data is of good technical quality and the study is carefully designed with many important controls included. While many conclusions rely on correlations, they all seem to hold when cross-compared, which is reassuring. The demonstration of relevance for HR but not for NHEJ is also an important point. There are some general points that need clarification:

1. The acetylation of MT is rather slow and appears to peak around 48-72 hours after DNA damage. However, repair would be expected to occur faster or at least the homologue partners search for which DSB mobility is important would be expected to occur earlier. What is the point of maximizing acMT at a time when most repair events have occurred? The apparent discrepancy between repair kinetics and acMT puts in questions the functional relevance of acMT for repair efficiency.

We thank the reviewer for this comment. We do not believe there is a discrepancy that undermines the functional relevance of tubulin acetylation for repair efficiency since we have already demonstrated that loss of tubulin acetylation impairs efficient repair (**Fig.4a-c**). But this observation prompted us to examine our data more carefully.

Our hypothesis is that the sustained increase in tubulin acetylation between 24–72 hours facilitate repair of 'hard-to-repair' foci, where additional DSB mobility would be particularly beneficial. While the majority of DSBs are repaired within 24 hours (**Fig. 1e**), consistent with the initial significant rise in tubulin acetylation, a subset of breaks persists until 72

hours. This was not clearly evident in the original **Fig.1e**. We have therefore provided additional analyses in **Extended Data Fig.2a**, showing that a population of cells retaining 1–4 53BP1 foci remains elevated at 72 hours compared with both the untreated control and the 96-hour time point. Consistent with these being persistent foci, their size increases significantly after 72 hours (**Extended Data Fig.2d**). To determine whether increased foci size negatively impacts mobility, we assessed DSB mobility at 8, 48, and 72 hours post-IR. Smaller foci (<0.2 μm^2) move faster, with similar mobility observed at all three time points. However, as foci size increases over time, we observe a correlation between high tubulin acetylation levels and increased mobility, even in foci >0.8 μm^2 (**Extended Data Fig.2e,f**). These data suggest that the purpose of maximising tubulin acetylation at later time points is to facilitate the mobility of persistent, larger foci (page 6, line 160).

We were further intrigued by the complete reset of tubulin acetylation levels at 96 hours, which suggested that an active DDR response persists at later time points and could help explain this incremental increase over time. To test this, we examined nuclear p21 levels under the same conditions, since p21 is a key DDR mediator that can remain elevated for several days post IR to induce cell cycle arrest (Di Leonardo et al., 1994, Genes Dev/PMID 7958916; Reyes et al., 2018, Mol Cell/PMID 30057196). Nuclear p21 levels mirror the tubulin acetylation pattern: elevated at 72 hours and returning to control levels at 96-120 hours (**Fig.1f and Extended Data Fig.1f,g**). Together, these data indicate that cells efficiently resolve the DDR between 72 and 96 hours, consistent with completion of DNA damage repair, at which point tubulin acetylation levels also return to baseline (page 5, line 138).

2. The authors partially address this issue (lines 270-297) but a more direct demonstration of the repair dynamics of breaks via HR is required to reconcile the slow time course of MT acetylation and its proposed role in HR. Can the authors provide additional data to support their claim?

We appreciate the reviewer's comment and have performed several new experiments to strengthen our conclusions. In doing so, we have also been able to further interrogate how DSB mobility facilitates HR and propose a model for the regulation of efficient homology search.

First, we want to emphasise that DSB mobility is increased as early as 8 hours post-irradiation, consistent with the early rise in tubulin acetylation and with the timing of HR. Our new data (described in response to Point 1) further demonstrate that at 8 hours, when most foci are small (<0.2 μm^2), even modest increases in tubulin acetylation are sufficient to drive DSB mobility.

We have now examined RAD51 foci resolution after cisplatin treatment in gCtrl and *ATAT1* KO cells (**Extended Data Fig.8d**) and observed a significant delay in foci resolution one day after washout (page 12, line 321). Furthermore, as suggested by Reviewer #2, we examined more specific markers of NHEJ, including p-DNA-PK(S2056), and found no delays in the resolution of these foci after IR (**Fig.5b and Extended Data Fig.8c**) (page 11, line 314).

Also as suggested by Reviewer #2, we used the well-established direct-repeat HR reporter assay to quantify HR efficiency. Experiments were performed in HEK293 cells to ensure efficient transfection, and we have now confirmed that αTAT1 loss also prevents efficient repair in this cell line (**Extended Data Fig.7**). We found that loss of tubulin acetylation (siαTAT1) leads to an approximately 40% reduction in HR efficiency (GFP-positive cells), while depletion of BRCA2 completely abolishes HR (**Extended Data Fig.9e**). This intermediate phenotype is expected: unlike BRCA2, which is essential for HR initiation, loss of tubulin acetylation does not prevent HR activation, as evidenced by normal RAD51 recruitment, but rather compromises its efficiency (page 12, line 342).

Finally, in the original manuscript we proposed that tubulin acetylation-mediated DSB mobility facilitates homology search, based on RAD51 foci adopting a more filamentous morphology, which associated with delays in homology search, as characterised by the laboratory of our collaborator Prof. René Medema (Friskes A. et al., 2025, BioRxiv). This led us to further consider the mechanistic basis by which DSB mobility promotes homology search. We were further guided by work from Eric Greene's lab (Crickard et al., 2020, Cell/PMID 32502392), who proposed that efficient homology search during HR is achieved by two cooperative mechanisms: random local search and RAD54L-driven active search. We hypothesised that the random local search corresponds to tubulin acetylation-driven DSB mobility, suggesting that tubulin acetylation and RAD54L could synergise to promote efficient homology search. To test this idea, we performed similar experiments to assess RAD51 foci morphology when cells lack both RAD54L and αTAT1. These new data show that loss of αTAT1 leads to an intermediate phenotype relative to RAD54L loss, consistent with the central role of RAD54L in this process. Strikingly, 16 hours after IR in cells that simultaneously lost RAD54L and αTAT1, we observed a significant synergistic increase in branched and coiled RAD51 filaments, indicative of greater defects in homology search (**Fig.5c-f**). Taken together, these data suggest that tubulin acetylation-mediated local DSB mobility and RAD54L-driven active search together contribute to efficient homology search (page 13, line 354).

3. It is difficult to know whether the observed change of DSB mobility is merely a bystander effect or represents an active defense mechanism. I would not expect the

authors to resolve this but toning down statements regarding directed causality and a frank discussion of these two scenarios should be included. Although the authors observe a correlation between nuclear shape fluctuations, DSB mobility and repair efficiency, they do not directly show that it is the change in nuclear shape fluctuations or altered mobility that contributes to delays in repair. These are merely correlations. In the absence of more direct evidence for a causal effect, for example, by altering mobility of DSBs via tethering or alike, this claim should be toned down.

We thank the reviewer for this important point. We apologise that the rationale for the αTAT1 /vimentin co-depletion experiments was not sufficiently clear in the original manuscript; these are in fact the key experiments establishing the mechanistic link between tubulin acetylation, nuclear shape, DSB mobility, and repair efficiency.

Briefly, our data show that DNA damage promotes nuclear softening, a feature previously observed but lacking mechanistic explanation. We demonstrate that tubulin acetylation regulates this process by facilitating vimentin transport away from the nucleus via the microtubule motor kinesin-1. Loss of this perinuclear vimentin cage renders the nucleus softer, consistent with prior work (Patteson et al., 2019, J Cell Biol/PMID 31676718). This in turn permits microtubule-driven nuclear deformation. The critical connection between nuclear shape fluctuations, DSB mobility, and repair is established by the rescue experiments: removing the vimentin cage, either by depleting vimentin directly or by depleting Plectin to promote vimentin displacement, is sufficient to rescue nuclear fluctuations (**Fig.3 and Extended Fig.6**), DSB mobility (**Fig.3 and Extended Fig.6**), and DNA damage repair efficiency (**Fig.4)** in the absence of αTAT1/tubulin acetylation. We therefore propose that the primary role of tubulin acetylation is to promote nuclear softening via vimentin displacement, enabling microtubule-driven fluctuations that support DSB mobility and repair. Restoring nuclear softness alone is sufficient to recapitulate all of these downstream effects, providing functional rather than merely correlative support for this pathway (page 11, line 296).

Directly manipulating DSB mobility without introducing significant confounding effects remains technically challenging, and we were therefore unable to pursue the tethering approach suggested by the reviewer. However, we believe the rescue experiments described above provide a more tractable and interpretable functional demonstration of the pathway. In addition, we have validated the role of kinesin-1 in this process: siRNA-mediated depletion of KIF5B, the predominant kinesin-1 in these cells, phenocopies αTAT1 depletion and prevents efficient DSB repair 24 hours after cisplatin washout (**Extended Data Fig.7c**) (page 11, line 304).

In response to Reviewer #3, we have now measure DSB mobility and nuclear fluctuations in cells treated with Cisplatin and Paclitaxel to inhibit microtubule dynamics. Consistent with data from the de Lange laboratory, we also found that Taxol abrogates DSB mobility (Lottersberger et al., 2015, Cell/PMID 26544937) but more importantly it also prevents nuclear fluctuations (**Extended Data Fig.3b-d**). These data support our original model (**Fig. 8**) and shows that dynamic microtubules are necessary for nuclear deformations and DSB mobility (page 8, line 212).

Furthermore, the new experiments exploring how αTAT1 depletion impacts homology search (Point 2 above) provide additional mechanistic rationale for how DSB mobility facilitates HR efficiency.

We hope these additional experiments that have strengthened the role of DSB mobility in DNA repair will satisfy the reviewer.

4. Related to the above point, I found the title of the manuscript not entirely appropriate as it highlights one of the weakest conclusions of the study. My recommendation would be to highlight in the title the role of MT acetylation in DNA repair since this is a stronger conclusion and make the changes in cellular and nuclear morphology a secondary conclusion.

We appreciate this suggestion. As described above, the rescue experiments demonstrate that defects in DNA repair caused by loss of tubulin acetylation can be reversed by promoting nuclear softness through vimentin loss or displacement, directly linking tubulin acetylation to nuclear shape and repair. In light of this, and in accordance with the reviewer's recommendation, we propose the following revised title: **"Tubulin acetylation regulates nuclear shape fluctuations to promote efficient DNA damage repair"**.

Additional technical points:

5. The authors show in figure 1 increased MT acetylation after DNA damage using imaging. These data should be validated by Western blotting.

We thank the reviewer for this suggestion. Single-cell imaging was our method of choice as it allows direct correlation of tubulin acetylation levels with DNA damage markers at the level of individual cells, a resolution that cannot be achieved by Western blot alone. We attempted Western blot validation of our immunofluorescence data; however, as is well-documented in the field, detection of K40 tubulin acetylation by Western blot is substantially less sensitive than immunofluorescence for reasons intrinsic to this modification. Specifically, the K40 epitope is located on the luminal face of the microtubule and is presented optimally in the context of polymerised microtubules: the conformation

is preserved in fixed cells used for immunofluorescence, as evidenced by the observations from many labs in the field (established in the original paper: Piperno et al., 1987, J Cell Biol/PMID: 2879846) that depolymerization of microtubules with Nocodazole prevents the detection of tubulin acetylation. As a consequence, Western blot detection of physiologically regulated changes in tubulin acetylation, which affect only a subset of the total tubulin pool, produces highly variable results that we do not feel would reliably inform the reader.

Importantly, treatment with HDAC inhibitors tubacin, TSA or by overexpressing αTAT1, which causes near-complete accumulation of acetylated tubulin, produces a robust Western blot signal (**Extended Data Fig.4b**). This confirms that the variability we observe under our experimental conditions reflects the sensitivity limitations of Western blot for detecting modest physiological changes in this modification, rather than a technical failure.

These considerations are well-recognised in the field. Quantification of tubulin acetylation changes by immunofluorescence is the established approach used by the leading laboratories working on this modification, including those of Nogales, Nachury and Roll-Mecak.

I did not understand the rationale for the experiment in which aTAT is co-depleted with vimentin. Why would loss of vimentin restore DSB mobility and repair efficiency? Please clarify.

We apologise that the rationale for these experiments was not sufficiently clear in the original manuscript. As detailed in our response to Point 3, these are central experiments for establishing the causal link between tubulin acetylation, nuclear softness, DSB mobility, and repair efficiency. In brief, because tubulin acetylation promotes nuclear softening by displacing the perinuclear vimentin cage, removing vimentin independently of tubulin acetylation bypasses the requirement for αTAT1 and restores downstream nuclear fluctuations, DSB mobility, and repair. We have revised the relevant sections of the manuscript to make this rationale explicit (page 10, line 261 and page 11, line 296).

REVIEWER #2

1. what is the DNA repair defect when ac-tubulin is absent? The defect in RAD51 foci appear minor, the phenotypes appear to be more dramatic with cisplatin, and it would be important to assess RAD51 foci in the same manner as H2AX in Figure 5b.

We appreciate the reviewer's comment. We want to highlight that we used IR to time repair and assess its dynamics early after DSB formation and observed that the resolution of RAD51 and γH2AX foci are delayed (**Fig. 5b**). As suggested by the reviewer, we have now quantified RAD51 foci in cells after cisplatin treatment where the defects in RAD51 foci resolution are significantly more pronounced (**Extended Data Fig.8d**) (page 12, line 321).

2. The conclusion made in regard to the impact of Ac-tubulin on DSB pathway choice are not sufficiently supported. The authors should take note that 53BP1 foci do not correspond to NHEJ repair efficiency. This is a common misconception. Rather, 53BP1 foci simply mark H2AX-ubiquitin and DSBs. They will also find BRCA1 and RAD51 foci co-localizing here. Therefore, statements about 53bp1 foci and NHEJ need to be modified.

We thank the reviewer for this important clarification and fully agree that 53BP1 foci mark DSBs broadly and their resolution kinetics cannot be used to infer NHEJ efficiency. We have removed that sentence entirely. To directly assess NHEJ efficiency, we used phospho-DNA-PK(S2056), a specific marker of active NHEJ, and found that foci resolution is not delayed in *ATAT1* KO cells after IR (**Fig.5b and Extended Data Fig.8c**). Together with the delayed resolution of RAD51 and γH2AX foci (**Fig.5b**), these data suggest that DSB mobility preferentially benefits HR rather than NHEJ (page 11, line 314). These new data are in line with the data showing that *ATAT1* KO B cells do not display defects in class switch recombination (**Extended data Fig.9**), further supporting that NHEJ efficiency is not impaired upon loss of tubulin acetylation.

3. Overall, it is not clear what Ac-tubulin is doing to promote DSB repair. If HR is affected, the authors need to prove this, for example, PARPi sensitivity assays would be useful, HR reporters could be used.

We have performed several new experiments to address this point and are confident these collectively demonstrate a role for tubulin acetylation-mediated DSB mobility in HR.

We thank the reviewer for suggesting using HR reporters. We have used the well-established DR-GFP system developed by the Jasin lab where the presence of GFP^{+ve} cells after *Sce*I-mediated DSBs indicates HR (Pierce et al., 1999, Genes Dev/PMID 10541549). We found that, while the overall percentage of GFP^{+ve} cells is low, consistent with previous work (Lim et al., 2024, Mol Cell/PMID 138244544), loss of tubulin acetylation (siαTAT1) leads to ~40% reduction in HR efficiency, whereas depletion of BRCA2 completely prevents HR (**Extended Data Fig.9e**). This intermediate phenotype

is to be expected since, unlike BRCA2, which is essential for HR activation, loss of tubulin acetylation does not prevent HR activation (as seen by recruitment of RAD51) but rather compromises its efficiency (page 12, line 342).

As suggested by the reviewer, we have also tested the sensitivity of *ATAT1* KO cells to PARPi (Olaparib and Talazoparib). We found that when compared to gCtrl cells, *ATAT1* KO cells are more sensitive to these inhibitors, with a further reduction in their viability of ~30% at higher drug concentrations (**Fig.7f,g**) (page 15, line 408).

We also sought to understand more directly how tubulin acetylation-mediated DSB mobility promotes HR. Our original data showed that RAD51 foci adopt a filamentous morphology in αTAT1-depleted cells, a hallmark of defective homology search characterised by Prof. René Medema's laboratory (Friskes A. et al., 2025, BioRxiv). Building on published work from Eric Greene's lab (Crickard et al., 2020, Cell/PMID 32502392), who identified two cooperative mechanisms of homology search: random local search and RAD54L-driven active search, we hypothesised that tubulin acetylation-driven DSB mobility constitutes the random local search component and that αTAT1 and RAD54L would therefore act synergistically. Consistent with this, co-depletion of RAD54L and αTAT1 produced a significant synergistic increase in branched and coiled RAD51 filaments at 16 hours post-IR compared with either depletion alone (**Fig. 5c-e**), providing direct evidence that these two mechanisms cooperate to ensure efficient homology search (page 13, line 354).

4. If the 'wiggle' is specific to HR but not NHEJ it could be assessed by synchronizing cells using the CDK4 inhibitor palbociclib and subjecting to IR in G1 (NHEJ only) vs S (HR mostly) phase released cells to observe repair specific differences. One would predict no wiggle in G1 cells post IR?

We thank the reviewer for their suggestion. We want to emphasise that we are not proposing that the 'wiggle' is regulated by cell cycle, nor that it only occurs in cells undergoing HR. Rather, we propose that the timing of DSB movement regulation (after IR) coincides with what is expected for late repair and HR (8–24 hours) and the significant increase in tubulin acetylation levels we observe (**Fig.1e**). Indeed, we do not see a significant cell cycle change during the time course that suggests a cell cycle associated mobility (**Extended Data Fig.1e**). To fully address this point and directly test if cell cycle could impact DSB mobility, we have arrested cells in G1 with Palbociclib for 24 hrs and use IR to induce DSBs. We found that, while 2 hours after IR DSBs move very little, consistent with non-arrested cells' data, 8 hours after IR DSBs acquire significant mobility comparable to non-arrested cells. Importantly, this mobility also coincides with increased tubulin acetylation in G1-arrested cells 8 hours after IR (**Extended Fig.8g-j**). Taken

together, these data demonstrate that DSB mobility and tubulin acetylation are not regulated by cell cycle *per se* but rather by timing after damage. This is consistent with previous work suggesting that increased DSB mobility has been associated with persistent damage or slower repair, hence its association with HR (reviewed in Chiolo et al., 2025, Nat Rev Mol Cell Biol/PMID 40097581) (page 12, line 325).

5. A confusing result is the time course that it takes for ac-tubulin to return to basal levels compared with the time it takes for 53bp1 foci to return. One would expect that cells have completed one or two mitoses by 96 hours. Could the authors confirm this and consider the impact of ac-tubulin and cell cycle progression.

We thank the reviewer for raising this important point and we address the underlying biology in full in our response to Reviewer #1, Point 1. Briefly, our data reveal that while most DSBs are resolved within 24 hours, a subset of persistent, larger foci remain until 72 hours (**Extended Data Fig. 2a,d**), and it is precisely for these hard-to-repair breaks that maximising tubulin acetylation appears most beneficial. New mobility analyses (**Extended Data Fig.2e,f**) show that as foci size increases over time, high tubulin acetylation levels correlate with increased mobility even in large foci (>0.8 μm^2), supporting a role for sustained acetylation in facilitating late-stage repair (page 6, line160). Furthermore, nuclear p21 levels mirror the tubulin acetylation time course: elevated at 72 hours and reset by 96 hours, indicating that an active DDR persists until this point and that the return of tubulin acetylation to baseline coincides with completion of repair (**Fig.1f and Extended Data Fig.1f,g**) (page 5, line138).

Regarding the reviewer's question about cell cycle progression, we have assessed whether loss of αTAT1 affects cell cycle distribution in response to IR and found no significant difference compared to control cells (**Extended Data Fig.8e**). This indicates that the differences in repair kinetics observed between *ATAT1* KO and control cells are not attributable to alterations in cell cycle progression (page 12, line 324).

6. Figure 6D, why are most foci able to resolve but only a small number not resolving?

This is an excellent question and one that fascinates us as well. Our hypothesis is that the DSBs that are not resolved when mobility is impaired arise in genomic regions that are inherently more difficult to repair, likely due to local chromatin organisation or DNA context rather than heterochromatin enrichment *per se*. Consistent with this idea, we plotted damage foci positioning within the nucleus over time in *ATAT1* KO cells and found that the overall distribution of foci does not differ from controls one day after washout (when no repair is observed in the *ATAT1* KO cells). However, a significant shift occurs after two days, at which point a subset of foci have been repaired and the remaining

persistent foci are located further from the nuclear envelope (**Fig. 6e,f**). The basis for this positional shift is not yet clear, but it raises the intriguing possibility that the genomic context of these breaks influences both their repairability and their subnuclear distribution. One possibility is that DSB position can directly impact repair pathway choice since DSBs associated with lamina-associated domains are repaired by alternative end-joining rather than HR (Lemaitre et al., 2014, Gen & Dev/PMID: 25366693). Chromatin conformation and local compaction state, which are increasingly recognised as important determinants of repair kinetics and pathway choice (Clouaire et al. 2018, Mol Cell/PMID: 30270107), could also explain this positional shift, with more open euchromatin associated with nuclear interior preferentially being repaired by HR and therefore likely to require DSB mobility (Bickmore, 2013, Annu Rev Gen Hum Genet/PMID: 23875797; Aymard et al., 2014, Nat Struct Mol Biol/PMID: 24658350). Alternatively, DSBs that are not resolved when mobility is impaired could arise in genomic regions that are inherently more difficult to repair, such as highly transcribed loci. These loci tend to occupy more central nuclear positions and can present challenges for HR due to the need to displace the transcriptional machinery and resolve R-loops (Marnef and Legube, 2021, Nat Cell Biol/PMID: 33837288). We have now included a brief discussion of these possibilities in the manuscript (page 18, line 493).

7. The project uses one cell line throughout. The primary findings should be confirmed in at least two other cell lines.

We appreciate this comment and the importance of validating these data in additional cell lines. We have now assessed the levels of tubulin acetylation in response to Cisplatin and the role of αTAT1 in DDR in HeLa, HEK293 and U2OS cells. Consistently with the RPE-1 data, we found that tubulin acetylation is increased in response to DNA damage and that loss of αTAT1 prevents efficient repair in these new cell lines (**Extended Fig.7a,b**) (page 10, line 287).

8. Colony formation assays using WT and TAT1 KO cells for cisplatin, IR, doxorubicin, PARPi sensitivity should be included.

We thank the reviewer for this suggestion. The widely used metabolic assays (e.g. MTT) or colony formation as these are not well-suited to experimental contexts where the expected outcome is senescence rather than cell death (**Fig.7c**). MTT measures mitochondrial metabolic activity, which is substantially elevated in senescent cells, leading to overestimation of cell number. Colony formation assays measure colony number/growth and the presence of large senescent cells can mask more subtle differences. Therefore, based on recent publications addressing this challenge (Foy et al,. 2024, NPJ Breast Cancer/PMID 38438376), we used CyQuant to directly measure

DNA content as a proxy for cell number, which is independent of metabolic activity and cell size, and is therefore a more reliable readout of proliferation arrest in this setting. We found that Cisplatin/Olaparib/Talazoparib treatment followed by washout lead to significant loss of viability (~30-40%) of *ATAT1* KO cells when compared to gCtrl (**Fig.7e-g and Extended Data Fig.10c**). More subtle effects were observed with Doxorubicin, which is generally very toxic due to its multiple targets and whose DSBs can be repaired by both HR and NHEJ, and with IR we only observed a trend that is not significant, likely due to fast repair via NHEJ (**Extended Data Fig.10d,e**). Importantly, these effects were observed 7 days after treatment, and not 4 days, consistent with cell cycle arrest and senescence (**Fig.7c**) (page 15, line 408).

REVIEWER #3

In this manuscript the authors advance a model whereby tubulin acetylation is directly responsible for effects on DNA damage repair through a mechanism that is not very clear but might involve an effect on the mechanical forces experienced by the nucleus. The hypothesis presented is interesting and there is an increased interest into the role of force in DNA damage, but the study feels preliminary and needs additional orthogonal investigations to support the model presented.

The authors use primarily imaging to address the question by documenting an increase in microtubule acetylation after treatment with cisplatin or irradiation, concomitant with a change in the mobility of DNA damage foci. One concern I have is that the effects that they see on tubulin acetylation are in fact a more general stress response due to cisplatin exposure or irradiation. Likewise, I am not convinced that the mechanical forces exerted by the microtubules do in fact affect DNA repair directly. Cisplatin is highly reactive and has been shown to affect many proteins and RNA. Similarly, radiation exposure will damage other components and not DNA alone.

As far as presentation, the manuscript is well written and the figures are high quality and well presented. Specific comments below that I hope the authors will take into consideration.

We thank the reviewer for their comments. We are confident that the substantial new data presented in this revision fully addresses the reviewer's concerns regarding the scope of the study. The revision adds further depth by providing additional orthogonal evidence and extending findings to new cell lines and conditions: 1) HR reporter assays, additional damage markers, and PARPi sensitivity data demonstrate the role of αTAT1 in HR; 2)

synergy between RAD54L and αTAT1 loss further supports a role for DSB mobility in homology search during HR; 3) Taxol-mediated stabilisation of microtubules, which prevents nuclear fluctuations and DSB mobility, further validates our model that dynamic microtubules are required to apply force at the nuclear envelope.

We also wish to address directly the concern that the observed effects may reflect a general stress response, which we think is fascinating. We would like to highlight two main points: 1) Increased tubulin acetylation has indeed been observed downstream of a variety of stresses (as we recently reviewed this subject: Donker and Godinho, 2025, Curr Opin Cell Biol/PMID 40220734), suggesting that regulation of nuclear mechanics by tubulin acetylation may have broader implications beyond the DNA damage response. In fact, as demonstrated in this work, overexpression of αTAT1 to induce tubulin acetylation independently of DNA damage is sufficient to drive nuclear deformations (**Fig.2j**); 2) Nevertheless, all phenotypes are specific to αTAT1 loss, are rescued by restoring nuclear softness independently of tubulin acetylation (e.g. vimentin or plectin depletion), and are reproduced by IR and doxorubicin, which have an entirely different mechanism of action to cisplatin. Furthermore, new rescue experiments using αTAT1 mutants that cannot bind α-tubulin or are catalytically inactive confirm that α-tubulin acetylation is the relevant output.

In Fig. 2h the response to nocodazole treatment is very close to that for Tat depletion. This is surprising since acetylated MTs are known to be resistant to nocodazole (one of the ways they were classified as stable). Are the acetylated microtubules lost during nocodazole treatment?

While acetylated microtubules are associated with increased stability and resistance to short treatments with nocodazole (e.g. 30 min/ Monteiro et al., 2023, EMBO J/PMID 37403793), prolonged nocodazole treatment (4 hours at 2μM) depolymerises all microtubules including acetylated ones, as we confirm by immunofluorescence (**Extended Data Fig.3**a).

Taxol will stabilize microtubules and induce acetylation. What happens if you add taxol which promotes MT acetylation? Is there a suppression of oscillations? With downstream effects on DNA repair?

We thank the reviewer for this interesting suggestion and have now performed the experiment. Consistent with the known role of Taxol in suppressing microtubule dynamics and in line with data from the de Lange laboratory showing that Taxol abrogates DSB mobility (Lottersberger et al., 2015, Cell/PMID 26544937), we found that Paclitaxel treatment prevents nuclear fluctuations and decreases DSB mobility (**Extended Data Fig.**

3b-d). Paclitaxel's dominant effect on microtubule dynamics demonstrates that microtubule dynamics are required alongside tubulin acetylation to generate the forces that drive nuclear deformations (supporting our model on **Fig.8**). In the original manuscript we used αTAT1 overexpression to directly test whether tubulin acetylation alone is sufficient to drive nuclear fluctuations in the absence of DNA damage, and found that it is (**Fig.2j**), but only when microtubule dynamics are preserved, as Taxol treatment now confirms (page 8, line 212).

We have not looked at how Taxol impacts DNA repair, mostly due to the long-time frames of treatments and the impact of Taxol on cells over time (toxicity, mitotic arrest). Taxol would also induce changes in cell cycle (G2/M arrest), which can impact the choice of repair pathway. Thus, these data would be difficult to interpret.

Doe a kinesin inhibitor have a similar effect as the kif5b depletion? In other words is the role of kinesin in transport or structural for the effects that they are seeing? Small point but since this is stated in the introduction, it has now been shown by several labs that kinesin is not sensitive to acetylation but that the increased activity of kinesin on acetylated microtubules is due to the fact that bundled microtubules are also acetylated and bundled microtubule promote increased motility.

We thank the reviewer for raising this point. We agree that the precise mechanism by which tubulin acetylation promotes kinesin-1 transport may involve indirect effects such as microtubule bundling (Balabanian et al., 2017, Biophys J/PMID 28978447) or change in protofilament interaction (Eshun-Wilson et al., 2019, PNAS/PMID 31072936), rather than a direct interaction between acetylated tubulin and the kinesin motor, and we have updated the relevant text accordingly (page 4, line 91). Importantly, this does not alter our conclusions: the key finding in our model is that tubulin acetylation promotes kinesin-1-mediated transport in cells, as demonstrated for organelle transport by us and others (Reed et al., 2006, Curr Biol/PMID 17084703; Monteiro et al., 2023, EMBO J/PMID 37403793; Hsu et al., 2026, BioRxiv – Sarah Cohen's lab), and that KIF5B is the downstream effector responsible for vimentin displacement.

Regarding a kinesin inhibitor, we used siRNA-mediated depletion of KIF5B, the predominant kinesin-1 in these cells, as the most specific perturbation available. KIF5B depletion phenocopies αTAT1 loss for both vimentin displacement, DSB mobility (**Extended Data Fig.6a,b and Fig.1j**) and we have now also shown that it impacts DNA repair (**Extended Data Fig.7c**) No kinesin-1-specific inhibitor that leaves microtubule binding intact is currently available to be able to distinguish between a structural from active role.

Figure 1i – the MSD plot. Please show MSD versus time and not on a log scale. What is the confidence interval/SD for these MSD traces?

We thank the reviewer for this comment. To clarify, **Fig.1i** shows the anomalous diffusion coefficient (α) derived from fitting a power law to the ensemble MSD data, which summarises whether foci exhibit sub-diffusive (α<1), Brownian (α=1), or super-diffusive (α>1) behaviour. The full MSD versus time plots, including standard deviation, are provided in **Extended Data Fig.2b**. We have now referenced this data better in the text (page 6, lines 150 and 157).

The section on lines 213-220 is highly questionable. Of course, AlphaFold3 will predict a strong interaction with a-tubulin! Structures of TAT with a-tubulin are in the literature for years and so AlphaFold is heavily biased towards these as they are in the training database. AlphaFold cannot be used to conclude that an enzyme does not modify other targets. If the authors are concerned about other aTAT targets, as one should always be with an enzyme, this is not going to convince many. A better way is to have more data for the mechanism presented.

We agree with the reviewer that AlphaFold3 predictions cannot be used to conclude that an enzyme does not modify other targets, and we have toned down these claims in the revised manuscript (page 9, line 236). The specific concern motivating the AlphaFold analysis was whether αTAT1 might acetylate histones, which are established DDR regulators and could confound our conclusions. To directly address this with functional data, as suggested by the reviewer, we have now performed rescue experiments using αTAT1 mutants established based on structure analyses that should not bind α-tubulin or are catalytically inactive (described in Friedmann et al., 2012, PNAS/PMID 23071314; Szyk et al., 2014, Cell/PMID 24906155). Neither mutant rescues DDR defects in αTAT1-depleted cells, while wild-type αTAT1 does (**Fig. 4f,g**), further supporting the importance of α-tubulin acetylation as the relevant output. This is further supported by previous work demonstrating that αTAT1 does not acetylate histones (Shida et al., 2010, PNAS/PMID 21068373), and by our HDAC inhibitor data showing that MS-275, which promotes histone acetylation without affecting tubulin acetylation, has no impact on nuclear fluctuations, whereas Tubacin, which selectively promotes tubulin acetylation, is sufficient to drive them (**Extended Data Fig.4b–e**).

Figure 3d and e – right most graph. What is the y axis? Is it really fluctuations that are plotted here?

We appreciate this is not clear in the main text and only fully described in the method sections. y-axis of these graphs corresponds to cumulative fluctuations, which are the raw fluctuation values converted into absolute values (as fluctuations can have both positive and negative integers) and summed for each frame. These summed frames were then added cumulatively to see the total cumulative fluctuation over time. We have now added more detail in the main text, which we agree will add clarity (page 7, line 192).

How were the cisplatin incubation time and washout chosen? are the kinetics on MT acetylation and vimentin displacement slower at lower concentrations? are they visible at all? cisplatin makes covalent interactions with its target and has been known to crosslink proteins, so even after the washout cisplatin adducts would remain. What is the rationale for the time windows used?

We treat cells with 2 μM cisplatin for 24 hours, which is at the lower end of concentrations used in the literature and was specifically chosen to generate a quantifiable DDR (Adeyemi et al., 2022, Mol Cell/PMID: 34597596; Schubert et al., 2022, EMBO Rep/PMID: 35156773; Paulsen et al., 2021/PMID: 34718766). This concentration reliably produces a reproducible number of DSBs, as confirmed by 53BP1 and γH2AX foci quantification (**Figs.1a and 5a**), while still permitting efficient repair in control cells, which is an important criterion for studying repair dynamics. Regarding kinetics at lower concentrations, we have not performed a full dose-response for all phenotypes, but we note that even at 2 μM the increase in tubulin acetylation and vimentin displacement are robust and reproducible across multiple experiments. With respect to the time windows, these were chosen empirically by monitoring repair in control cells: in control/untreated cells, 53BP1 and γH2AX foci resolve substantially within 24 hours of washout and are largely absent by 48 hours, also shown in the neutral comet assay which measures DSBs directly, confirming that the timepoints used capture active repair rather than residual unresolvable damage (**Fig. 4a-e and Fig. 5a**). The washout itself is performed by washing cells four times with fresh medium to minimise residual extracellular drug. Importantly, we observe identical phenotypes using doxorubicin and IR, which have entirely different mechanisms of action and do not involve platinum adducts, providing strong evidence that the effects we describe reflect a general DDR rather than cisplatin-specific chemistry.

Did the authors perform ICP-MS to analyze platinum in their samples?

We have not performed ICP-MS to analyse platinum in our samples, but we believe the concern underlying this question is fully addressed by our experimental design. The reviewer's implicit concern is that residual platinum adducts persisting after cisplatin washout could drive the phenotypes we observe independently of a bona fide DDR. We would make two points in response. First, cisplatin-DNA adducts are not a confounding

variable but rather the source of the DSBs we monitor: their persistence is precisely what sustains the DDR signal at later timepoints, and the kinetics of repair we observe with 53BP1, γH2AX, RAD51, and the neutral comet assay directly report on the resolution of these lesions rather than on residual drug levels. Second, and most critically, IR produces no chemical adducts or reactive platinum species whatsoever, yet we observe identical phenotypes after IR: temporal dynamics of tubulin acetylation, vimentin displacement, nuclear fluctuations, and DSB mobility, with the same dependence on αTAT1 (**Figs.1 and 3**). If the effects we describe were driven by residual cisplatin toxicity rather than the DNA damage response per se, they would not be reproduced by IR. The fact that they are reproduced argues strongly against a platinum-specific mechanism and in favour of a general DDR-dependent response.

The primary measure of DNA damage presented is through 53BP1 foci and H2AX imaging. Is for example DDB recruitment to damage sites also changed in the TAT KD?

We appreciate this comment. DDB (damage-specific DNA binding protein) is primarily associated with nucleotide excision repair (NER) rather than DSB repair, and cisplatin-induced intrastrand crosslinks are processed by NER. The primary lesions relevant to the repair defects we describe are DSBs, which are monitored by our DSB markers. We have now expanded our panel to include RAD51 (specific HR marker) and p-DNA-PK(S2056) (specific NHEJ marker), in addition to γH2AX and 53BP1 (**Fig.5b and Extended Data Fig.8c,d**). No defects reported in marker recruitment but rather a delay in resolution specifically for HR-associated foci, consistent with a role for tubulin acetylation in HR efficiency (page 11, line 314).

Is vimentin displacement due to the direct effects of the cytotoxic treatment on this filament?

Our data strongly demonstrates that vimentin displacement is not due to cytotoxic effects of any treatment. Indeed, in cells depleted of αTAT1 or KIF5B, vimentin displacement can be inhibited even in the presence of Cisplatin (**Extended Data Fig.6a,b**). Conversely, vimentin displacement can be induced independently of DNA damage by depletion of Plectin, as also shown by others (Jiu et al., 2015, Cell Rep/PMID 26027931) (**Extended Data Fig.6e,f**).

Can the authors confirm their findings in another cell line? How is this response in cells that have a large amount of acetylated tubulin to begin with? Do they have different rates of repair? This would seem relevant in terms of in vivo sensitivity to DNA damage.

This important point was also raised by Reviewer #2, and as described in detail in our response to Reviewer #2, Point 7, we have now validated our primary findings in HEK293, HeLa and U2OS cells. In all cell lines, Cisplatin treatment led to a significant increase in tubulin acetylation and αTAT1 depletion compromised efficient DNA repair (**Extended Data Fig.7a,b**).

While we agree that different levels of tubulin acetylation could impact repair dynamics, this is difficult to assess since, for example in the case of cancer cells other confounding mutations could impact DNA repair. What is really interesting is that loss of αTAT1 can impair repair even in cancer cell lines, with potential implications for cancer treatments. We have now tested whether *ATAT1* KO cells are more sensitive DNA damage agents and found that Cisplatin/Olaparib/Talazoparib treatment followed by washout lead to significant loss of viability (~30-40%) of *ATAT1* KO cells when compared to gCtrl (**Fig.7e-g**). More subtle effects were observed with Doxorubicin, which is generally very toxic due to its multiple targets and whose DSBs can be repaired by both HR and NHEJ, and IR, likely due to fast repair via NHEJ (**Extended Data Fig. 10d,e**). Viability loss was only observed 7 days after treatment, and not 4 days, consistent with increase in cell cycle arrest and senescence (**Fig.7c**). The sensitivity of *ATAT1* KO cells to the PARPi Olaparib and Talazoparib is particularly exciting as it further supports a role for tubulin acetylation in HR (page 15, line 408).

Figure 5c- how were Rad51 filaments quantified? The images presented are not convincing that they can distinguish between filaments versus puncta. Higher resolution images will be required to make this assessment and an objective, quantitative criteria needed on how these two states are classified.

Quantification of RAD51 foci morphology is done by manually inspection of high-resolution images as described by the laboratory of our collaborator Prof. René Medema (Friskes A. et a., 2025, BioRxiv). We have now repeated this experiment with additional conditions to further test the role tubulin acetylation and DSB mobility in the homology search, which not only adds more mechanistic detail but also addresses this reviewer's concern. Guided by work from Eric Greene and colleagues (Crickard et al., 2020, Cell/PMID 32502392), who proposed that efficient homology search is achieved through two complementary mechanisms: random local search and RAD54L-driven active search, we hypothesised that tubulin acetylation-driven DSB mobility corresponds to the random local search component. We tested this by assessing RAD51 foci morphology in cells lacking both RAD54L and αTAT1, as described in full in our response to Reviewer #1, Point 2. Briefly, loss of αTAT1 alone produced an intermediate phenotype relative to RAD54L loss, and simultaneous depletion of both produced a significant synergistic increase in branched and coiled RAD51 filaments at 16 hours post-IR (**Fig. 5c-f**),

indicative of greater homology search defects than either perturbation alone. We believe these new data, together with the higher-resolution images now included, provide clearer and more objective evidence for the phenotypic distinctions between RAD51 filament classes (page 13, line 354).

We thank all reviewers for their constructive comments during the revision of this work, which allowed us to improve it significantly.

REVIEWER #1

Wallis et al. in this revised manuscript have addressed most of my technical points to a satisfactory degree.

We thank the reviewer for the positive comments.

However, the most important issue of the mechanistic basis for the effect of MT acetylation on DSB mobility has not been resolved. It remains unclear how MT-acetylation communicates with chromatin to alter DSB mobility; this is a central element of the proposed model but is not demonstrated. The authors' suggestion that this occurs via changes to nuclear shape is not convincing nor very plausible. It is difficult to envision how large scale changes in nuclear shape promote changes in the mobility of specific loci, especially given that the authors propose that this mechanism is of relevance for persistent unrepaired DNA breaks. The model in figure 8 does not illustrate a mechanism and no experiments to directly test this major conclusion and key step in the model (as mentioned in the title) are presented. One way to address this would be to manipulate nuclear shape by mechanical means and demonstrated resolution of persistent DSBs. In the absence of such experiments, the study remains purely phenomenological and the major central mechanistic conclusion of a role of nuclear shape in promoting DSB mobility is not supported.

We appreciate this comment and concern. However, our experiment where we, manipulated nuclear mechanics via depletion of vimentin or plectin does exactly this: changes nuclear stiffness thereby enabling microtubules to deform the nucleus in the absence of tubulin acetylation. While we understand that manipulating nuclear mechanics more directly by mechanical means would have been great, we currently lack the ability to do so.

In this regard, we have now changed the title, as suggested, to avoid implying a causal link between nuclear fluctuations and DNA repair: "Tubulin acetylation regulates nuclear fluctuations and DNA repair". We are confident that this title reflects the main findings in this work: that tubulin acetylation regulates nuclear fluctuations upon DNA damage and that it promotes efficient DNA repair.

We have also toned down these conclusions in the main manuscript, as listed below:

Page 4, line 115
Page 10, line 279
Page 11, line 303
Page 15, lines 426-434

Specifically, and as suggested by reviewer #2, we added the following sentence at the end of Discussion' 1st paragraph to highlight this limitation in our study: "*While our data define a mechanism by which microtubule forces drive nuclear envelope deformation, how these forces propagate to chromatin and modulate DSB mobility remains an important open question*."

REVIEWER #2

This is an outstanding study and much improved.

We thank the reviewer for the positive comments which were very much appreciated by the team.

Reviewer #2's additional comments on Reviewer #1's current concerns:
I agree to some extent with Reviewer 1's comments. However, these points do not detract from the quality of the work or the important new insights presented in this study. Although the manuscript does not directly address the mechanistic impact of nuclear shape on DSB repair, I think this would be a challenging—and perhaps not currently feasible—question to address experimentally, as manipulating nuclear shape is likely to have numerous unintended consequences.

I believe the issues raised by Reviewer 1 could be acknowledged in the Discussion and proposed as important directions for future studies. Nevertheless, the authors convincingly demonstrate a novel and compelling connection between these phenomena, providing biologically relevant insights into the events that occur during DSB repair. Therefore, I strongly urge Nature Cell Biology to accept this manuscript in its current form.

Overall, in my opinion, the approaches used are appropriate and sufficient to support the conclusions. The authors just need to acknowledge the limitation mentioned by R1.

We thank the reviewer for these comments and support of our work. We agree with highlighting this limitation of our study in the discussion. We have now added the following sentence at the end of Discussion' 1st paragraph: "*While our data define a mechanism by which microtubule forces drive nuclear envelope deformation, how these forces propagate to chromatin and modulate DSB mobility remains an important open question*."

We have toned down this part of our work throughout the manuscript and changed the title (see response to reviewer #1 above).

REVIEWER #3

The authors have overall addressed my concerns.

We thank the reviewer for the positive comments.

As they revise their manuscript, I hope that the authors will also consider the fact that cisplatin (Horibata et al /PMID42241284), IR (Zamansky et al./PMID 1576000, Goldblum et al/PMID34343476) and doxorubicin (Rabkin et al. PMID3325651), all agents used in this study to induce DNA damage also act directly on microtubules by destabilizing them and inducing damage. These microtubule abnormalities persist long after the damaging agent has been removed, with kinetics not dissimilar to what the authors describe as the kinetics of tubulin acetylation. The authors should mention this in their discussion as it could provide another axis that connects the damage agents to MT effects, changes in acetylation followed by feedback on repair (and possibly add confounding factors to the data interpretation).

We thank this reviewer for the suggestion and references. While our data show no evidence of microtubule depolymerization in the treatments performed here, we cannot rule out that other changes, such as lattice damage and microtubule dynamics, could indeed impact tubulin acetylation more directly. We have now added this important point to the discussion (Page 16, line 454).

Fig 5c-f data are a nice addition

We thank the reviewer for this comment. The new data helps place DSB mobility in the context of HR.

Small point: In Discussion it is stated: “This mechanism reveals an unexpected role for cytoplasmic microtubules in reshaping nuclear mechanics and broadens current models of how spatial dynamics contribute to genome maintenance” The authors should consider rephrasing this – there is nothing unexpected about the role of cytoplasmic MTs in affecting nuclear mechanics. Here is a recent review on this very topic https://www.sciencedirect.com/science/article/pii/S0962892421000702.

This statement needs to be more focused to cover what is novel in their study.

We have now removed this sentence and amended this entire paragraph to focus on our

findings and tone down the causal link between nuclear fluctuations and DNA repair, as requested by reviewer 1 (Page 15, lines 426-434).

Reviewer #3 (Remarks on Protocol(s)):

Methods are sufficiently well described.
